# Phylogenomic analyses of KCNA gene clusters in vertebrates: why do gene clusters stay intact?

**DOI:** 10.1186/1471-2148-7-139

**Published:** 2007-08-15

**Authors:** Simone Hoegg, Axel Meyer

**Affiliations:** 1 Lehrstuhl für Zoologie und Evolutionsbiologie, Department of Biology, University of Konstanz, 78457 Konstanz, Germany

## Abstract

**Background:**

Gene clusters are of interest for the understanding of genome evolution since they provide insight in large-scale duplications events as well as patterns of individual gene losses. Vertebrates tend to have multiple copies of gene clusters that typically are only single clusters or are not present at all in genomes of invertebrates. We investigated the genomic architecture and conserved non-coding sequences of vertebrate *KCNA *gene clusters. *KCNA *genes encode shaker-related voltage-gated potassium channels and are arranged in two three-gene clusters in tetrapods. Teleost fish are found to possess four clusters. The two tetrapod *KNCA *clusters are of approximately the same age as the *Hox *gene clusters that arose through duplications early in vertebrate evolution. For some genes, their conserved retention and arrangement in clusters are thought to be related to regulatory elements in the intergenic regions, which might prevent rearrangements and gene loss. Interestingly, this hypothesis does not appear to apply to the *KCNA *clusters, as too few conserved putative regulatory elements are retained.

**Results:**

We obtained *KCNA *coding sequences from basal ray-finned fishes (sturgeon, gar, bowfin) and confirmed that the duplication of these genes is specific to teleosts and therefore consistent with the fish-specific genome duplication (FSGD). Phylogenetic analyses of the genes suggest a basal position of the only intron containing *KCNA *gene in vertebrates (*KCNA7*). Sistergroup relationships of *KCNA1/2 *and *KCNA3/6 *support that a large-scale duplication gave rise to the two clusters found in the genome of tetrapods. We analyzed the intergenic regions of *KCNA *clusters in vertebrates and found that there are only a few conserved sequences shared between tetrapods and teleosts or between paralogous clusters. The orthologous teleost clusters, however, show sequence conservation in these regions.

**Conclusion:**

The lack of overall conserved sequences in intergenic regions suggests that there are either other processes than regulatory evolution leading to cluster conservation or that the ancestral regulatory relationships among genes in *KCNA *clusters have been changed together with their regulatory sites.

## Background

Higher phenotypic complexity of vertebrates has been often associated with a higher number of genes produced through whole genome duplications [1]. Genome projects and a deluge of sequence data showed that vertebrates often possess more than one copy of a gene or gene clusters [2-5] where invertebrates have only one. This observation together with synteny data, led to the formulation of the 2R hypothesis, which proposes two rounds of genome duplication in early vertebrate evolution [6-9]. An additional duplication event occurred in the lineage of ray-finned fish, the so-called fish-specific genome duplication (FSGD, 3R) [10-17]. While the duplicated genes are expected to be redundant in their function immediately following the duplication, their functions often diversify later [18,19]. Possible scenarios are that one copy evolves a new function (neofunctionalization) or the ancestral functions get subdivided between the paralogs (subfunctionalization) [20,21]. In most cases, however, one copy is expected to accumulate mutations that lead to a non-functional gene and finally to gene loss [20-23].

Clusters of genes belonging to the same gene family can give important insights into the evolutionary history of a genomic region, both in terms of gene loss events as well as for the evolution of the regulatory sequences surrounding it. The most prominent examples for this type of approach are the Hox gene clusters [24-28], a family of transcription factors that are not only arranged in uninterrupted clusters on the chromosome but are even expressed during embryogenesis according to their chromosomal order – a phenomenon called colinearity [reviewed in [29]. But also other gene clusters have been studied in this regard such as the ParaHox cluster [30](Siegel et al. submitted) and Fox clusters [31]. Both belong to other families of transcription factors with multiple cluster copies in vertebrate genomes. Less research in this respect has been performed on non-developmental genes.

We were interested if the patterns of molecular evolution that are found in *Hox *clusters can also be identified in other gene clusters and if it is also possible to identify conserved non-coding regions in them. *KCNA *genes are arranged in two uninterrupted clusters of three genes each which are located on chromosomes three and six in mouse and on chromosomes one and twelve in humans [32,33]. *KCNA *genes code for the Kv1 family of shaker-related voltage-gated potassium (K^+^) channels, those consist of six transmembrane (TM) segments and, the most important part, the pore loop (P-region), which ensures ion selectivity [34,35]. The Kv channels are active as tetramers, usually heterotetramers. Sodium (Na^+^) and calcium (Ca2^+^) channels, on the other hand, are monomers that consist of four linked domains, each of which is homologous to a single 6-TM K^+ ^channel [36]. Studies on the genomic organization of these genes so far have been limited to mammals. Upstream regulatory factors and potential regulatory elements have not been described previously. The number of KCNA genes and their genomic arrangement in ray-finned fish has not been studied before. Here we extend these comparative genomic approaches to other lineages of vertebrates and compare them to the situation in the genome of invertebrates.

We conducted an analysis of complete genome sequences of tetrapods and ray-finned fish *KCNA *genes and investigated the entire genome content when possible. In an effort to increase the database on basal fish, for which such data were not available prior to this study, we added new data using a PCR approach with universal primers and cloned the PCR products. We also included data from the non-vertebrate chordates *Branchiostoma floridae *and *Ciona intestinalis *for a better estimate of the age of this gene family. We constructed gene trees to permit inferences about the timing of the gene duplications and the cluster duplications. Furthermore, we aimed to test the hypothesis that the conservation of the genomic architecture of a gene cluster is linked to the content of conserved elements within the intergenic regions. To this end we investigated the 3-gene-cluster of *KCNA *genes (Kv1, shaker-related potassium channels) in several species of vertebrates. In tetrapods, two clusters exist (*KCNA3-KCNA2-KCNA10, KCNA6-KCNA1-KCNA5*), while teleosts were found to have four clusters.

## Results

**Figure 1:**
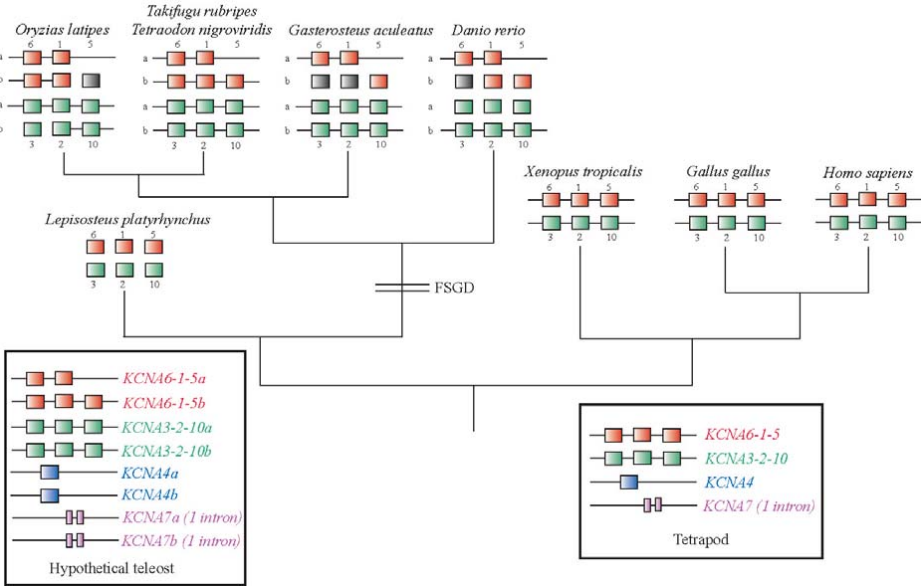
**Phylogenetic scheme of *KCNA *cluster evolution**. Non-connected genes indicate missing linkage/genomic data. Grey squares show hypothetical genes that most likely exist, but are still missing from the current versions of genomic databases. The boxes include genes that are not part of KNCA clusters. The teleost state is hypothetical since we found duplicated *KCNA4 *genes in *Gnathonemus petersi *and duplicated *KCNA7 *genes in *Oryzias latipes*, but no teleost studied so far showed the full set of duplicated genes.

Tetrapods, such as human, chicken and frog, have two three-gene-clusters (3-2-10, 6-1-5) and two additional genes, *KCNA4 *and *KCNA7*, which are located elsewhere in the genome (Figure 1). Teleost fish such as pufferfish, medaka, stickleback and zebrafish were found to have four clusters of *KCNA *genes. According to available results from genome sequencing projects, *KCNA5a *was lost. For *KCNA7*, duplicates were found in medaka and two copies of *KCNA4 *are present in the osteoglossomorph elephantnose fish (*Gnathonemus petersi*). All of these genes are conserved in their transmembrane domains and in the pore-loop region, but the other parts of these genes are highly variable and impossible to align between different members of this gene family. In the tunicate *Ciona intestinalis *only one KCNA gene was found while we received several BLAST hits for the amphioxus (*Branchiostoma floridae*) genome.

**Figure 2:**
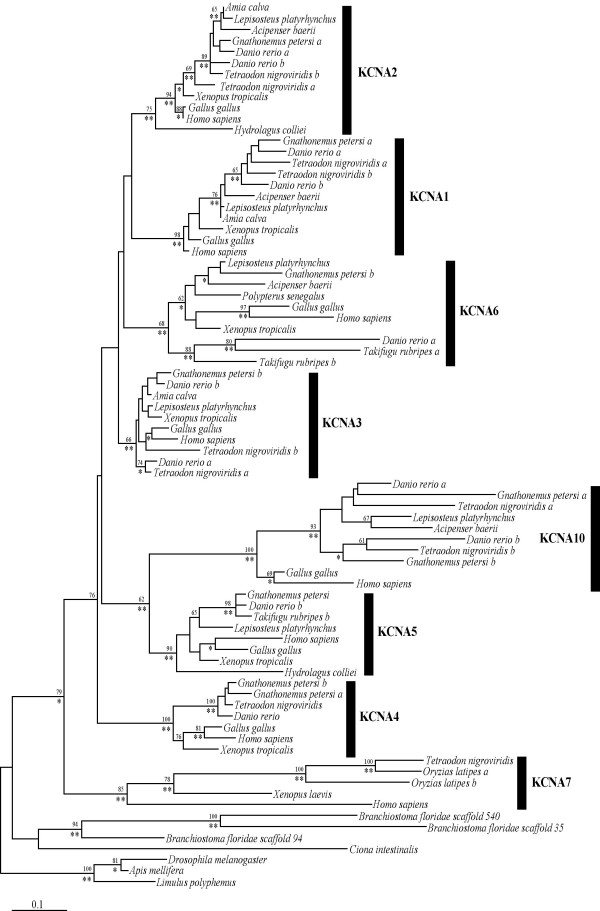
**Maximum likelihood tree of *KCNA *gene family **based on 80 sequences and 364 amino acid positions. The tree was obtained using PhyML [5], with 500 bootstrap replicates, values are shown by the first numbers. Posterior probabilities as obtained by MrBayes 3.1.1 [59] (100 000 generations) are indicated with asterisks. (** = 100% PP, * = 99–95% PP)

**Figure 3:**
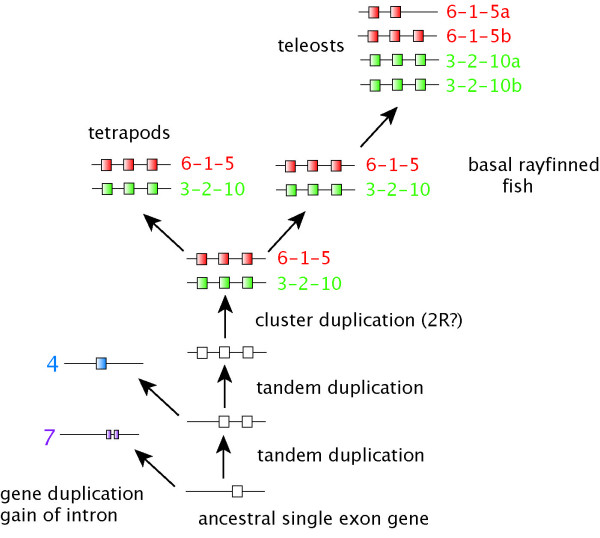
**Proposed scenario for the evolution of *KCNA *genes and clusters in vertebrates**. Based on our analyses we suggest that all KCNA genes are derived from an ancestral intronless gene, as all genes included from *Branchiostoma floridae *are intronless and that *KCNA7 *in vertebrates independently gained an intron. Two tandem duplications led to the three gene clusters found in today's genomes, which was probably duplicated initially before the origin of the gnathostomes. Probably this is linked to the second genome duplication (2R) during vertebrate evolution. The four clusters in teleost fish originated through the fish-specific genome duplication (FSGD, 3R).

A phylogenetic analysis of all *KCNA *genes suggests a diversification of KCNA genes in the vertebrates and basal position of *KCNA7 *among them (Figure 2); it is the only vertebrate *KCNA *gene that contains a single intron while all others are intronless. In invertebrates, such as *Ciona intestinalis *and *Drosophila melanogaster*, the *KCNA/shaker *gene has multiple introns, but the position of the introns is not conserved between invertebrates and vertebrates (data not shown). Genes identified from the *Branchiostoma floridae *genome, however, consisted of a single exon indicating that the vertebrate *KCNA7 *gene acquired its intron independently (Figure 3). The three best BLAST hits from *Branchiostoma floridae *with vertebrate KCNA genes were included in the phylogenetic analysis. All three genes are positioned on different scaffolds and no clusters were found. We obtained additional hits with the *Branchiostoma *genome, some of which also received KCNA best hits in BLAST searches against the GenBank database. However, their high sequence divergence made reliable estimation of their phylogenetic position relative to the outgroup in the *KCNA *gene tree impossible. The three *Branchiostoma KCNA *genes included in the analysis form a monophyletic group basal to the vertebrate genes, indicating a series of independent gene duplication within the amphioxus lineage.

Within the vertebrate part of the tree, *KCNA5 *and *KCNA10 *form a monophyletic group, as do *KCNA1 *and *KCNA2 *(Figure 2). This finding supports the hypothesis that the two clusters are the result of a complete duplication of the original cluster rather than of independent tandem duplications, which would have phylogenetically grouped neighboring genes on chromosomes in the tree. Only the *KCNA3/KCNA6 *gene pair does not reflect a pattern of whole genome duplication(s).

Our PCR based approach yielded two *KCNA *genes from *Hydrolagus colliei *(spotted ratfish, *KCNA2, 5*), four genes from *Acipenser baerii *(sturgeon, *KCNA1, 2, 6, 10*), six genes from *Lepisosteus platyrhynchus *(gar, *KCNA1, 2, 3, 5, 6, 10*), and nine genes from *Gnathonemus petersii *(elephantnose fish, *KCNA1b, 2a?, 3b, 4a, 4b, 5b, 6b, 10a, 10b*) [for accession numbers see Additional file 4]. We performed phylogenetic analyses of the ancient duplicates (*KCNA3-6*, *KCNA2-1 *and *KCNA10-5*) and used *KCNA4 *as outgroup [see Additional files 1, 2, 3]. *KCNA4 *is not part of the clusters, but phylogenetically closely related without obvious acceleration of evolutionary rates (Figure 2). In this way, we avoid reconstruction artifacts due to a too divergent outgroup as would expected be with the ancestral *KCNA7 *gene.

Our *KCNA3/6 *dataset encompassed 42 sequences and included sequences from human, chick and frog (378 amino acid positions) and resulted in Maximum Likelihood and Bayesian inference trees which were congruent for the well supported nodes and showed only minor differences within the not strongly resolved parts of the tree [see Additional file 1]. While the *KCNA3 *genes were phylogenetically separate from the *KCNA6 *genes, the resolution within each of these sets of orthologous genes is poor and the relationships, especially among the fish paralogous groups, could not be identified with confidence. The assignment into "a" and "b" paralogs was done based on the position in the clusters when genomic data was available, and the newly determined orthologous KCNA genes for the fish were assigned names accordingly. The evolutionary rates differ clearly between the orthologous groups (*KCNA3 *vs. *KCNA6*) as well as between the fish-specific a- and b-paralogs.

The *KCNA2/1 *analysis shows also a clear division between these two set of genes, but there are no obvious rate differences between them, only within the teleosts somewhat increased rates are apparent [see Additional file 2]. For both genes, the non-teleost fish sequences (*Acipenser, Amia, Lepisosteus*) are pro-orthologous of the FSGD as had been proposed in previous studies [10,37,38]. Studies based on *Hox *genes as well as other nuclear genes (*sox11, tyrosinase, fzd8, POMC*) found a phylogenetic timing of the FSGD after the divergence of Polypteriformes (bichir), Acipenseriformes (sturgeons), Lepistosteidae (gar) and Amiidae (bowfin), but before the teleost radiation including the most basal group of the Osteoglossiformes [10,30]. The *Gnathonemus KCNA2 *sequence is positioned basal to the duplication in the gene tree. The *Gnathonemus KCNA1 *sequence is grouped with the b-paralog in the *KCNA1 *gene tree. But also in this analysis, statistical support for most of the nodes is lacking.

The *KCNA5/10 *tree shows a similar pattern as the *KCNA3/6 *analysis with a clear acceleration of evolutionary rates in the *KCNA10 *genes, a trend that is even more pronounced in ray-finned fish [see Additional file 3]. The *Hydrolagus KCNA5 *sequence is phylogenetically grouped with the other KCNA5 genes with good phylogenetic support (94%BP, 100%PP), indicating that the duplication of the clusters leading to *KCNA5 *and *KCNA10 *occurred before the divergence of cartilaginous fish as previously proposed [39].

Even though the phylogenetic analyses of *KCNA *genes cannot pinpoint the duplication event in the fish phylogeny with a high degree of certainty, the numbers of identified genes implies that the phylogenetic split of basal lineages that include *Acipenser*, *Lepisosteus *and *Amia *from the fish stem lineage precedes the duplication event, while duplicated *KCNA4 *and *KCNA10 *genes suggest that Osteoglossomorphs (*Gnathonemus petersi*) diverged after the 3R event. This interpretation is in agreement with previous analyses on the phylogenetic timing of the FSGD [10].

We analyzed the non-coding regions of the complete clusters using the Tracker software [40], which detects clusters of such phylogenetic footprints (putative transcription factor binding sites) termed cliques (FC). Following the definition of phylogenetic footprints as in Tagle et al. [41], we only compared sequences with an additive evolutionary time of at least 250 million years, i.e. species that diverged at least 125 million years ago, and therefore we excluded comparisons of orthologous pufferfish clusters [42,43]. For the *KCNA 6-1-5 *comparisons, we included the partial *Tetraodon *KCNA 5-1b cluster, since it was currently the only available genomic sequence for this paralogon. Because of a large sequence gap in the intergenic region of the medaka *KCNA 6-1b*, we omitted this sequence from the analysis. We also could not include the *Danio *KCNA 3-2-10a cluster, since no linkage information for KCNA2a was available in the current assembly. For these analyses we were able to include a total of 20 clusters (11*KCNA 3-2-10*, 9 *KCNA 6-1-5 *and we obtained 670 FC of which 182 are shared by more than two species. The alignments and positions of those are given in the Additional file 5. Since an analysis using untreated sequences resulted an unusual high number of cliques shared only between the two paralogous human clusters as well as between the two *Xenopus *clusters, an effect probably due to the abundance of repetitive elements, we used RepeatMasker to remove those elements and repeated the analyses [44]. This strategy reduced the number of intra-species hits successfully.

We also analyzed the number of FCs and the length of conserved sequences between orthologous and paralogous clusters. Between orthologous clusters, the number of conserved elements follows the expected patterns, at least within tetrapods and the orthologous fish clusters respectively (Tables 1, 2). Paralogous fish *KCNA*-gene clusters share surprisingly few conserved elements.

**Table 1:** Pairwise comparison of *KCNA 6-1-5 *clusters Above the diagonal are the numbers of shared cliques (clusters of phylogenetic footprints) based on Tracker analyses; below are the complete lengths of shared elements. Excluded direct comparisons between pufferfish clusters are printed in bold. "a" and "b" refer to the duplicated fish clusters.

615	*Hs*	*Gg*	*Xt*	*Tra*	*Tna*	*Ola*	*Gaa*	*Dra*	*Tnb*
*Hs*	-	45	25	7	7	7	5	5	5
*Gg*	2959	-	30	10	5	8	5	5	5
*Xt*	1871	2100	-	6	5	6	4	4	3
*Tra*	528	622	345	-	**17**	19	17	7	2
*Tna*	517	378	311	**2340**	-	16	11	6	1
*Ola*	571	513	370	2354	1808	-	16	7	0
*Gaa*	214	200	218	1717	1553	1815	-	7	0
*Dra*	244	173	205	1245	1212	1358	1039	-	0
*Tnb*	195	221	140	102	40	0	0	0	-

Abbreviations are standing for human (Hs), chicken (Gg), frog (Xt), zebrafish (Dr), stickleback (Ga), medaka (Ola), and the two pufferfishes (Tn, Tr).

**Table 2:** Pairwise comparison of *KCNA 3-2-10 *clusters Above the diagonal are the numbers of shared cliques (clusters of phylogenetic footprints) based on Tracker analyses; below are the complete lengths of shared elements. Excluded direct comparisons between pufferfish clusters are printed in bold. "a" and "b" refer to the duplicated fish clusters.

*3210*	*Hs*	*Gg*	*Xt*	*Gaa*	*Ola*	*Tra*	*Tna*	*Trb*	*Tnb*	*Gab*	*Drb*
*Hs*	-	28	24	5	6	5	5	8	6	13	10
*Gg*	1788	-	16	9	7	3	6	8	4	6	6
*Xt*	1375	1077	-	10	13	6	10	11	8	11	12
*Gaa*	450	514	507	-	47	41	39	8	8	7	9
*Ola*	445	462	618	4560	-	37	34	4	5	4	7
*Tra*	484	282	395	4129	3599	-	**33**	3	6	4	5
*Tna*	412	452	497	4270	3520	**3052**	-	6	8	9	6
*Trb*	559	408	553	827	460	444	505	-	**55**	75	10
*Tnb*	439	303	414	1005	586	703	741	**4966**	-	70	8
*Gab*	730	370	550	803	460	493	647	5952	5527	-	8
*Drb*	788	469	602	805	586	600	574	980	813	906	-

Abbreviations are standing for human (Hs), chicken (Gg), frog (Xt), zebrafish (Dr), stickleback (Ga), medaka (Ola), and the two pufferfishes (Tn, Tr).

Interesting in this regard is the comparison between paralogous clusters (e.g. *Homo *3-2-10 vs. *Homo *6-1-5). While one might expect to find a reduced number of conserved sequences compared to the orthologous comparisons (e.g. *Homo *3-2-10 vs. *Xenopus *3-2-10), this is not the case for comparisons among tetrapods. The human *KCNA3-2-10 *cluster shares more FCs with its paralog *KCNA6-1-5 *than with the more closely related frog *KCNA3-2-10 *cluster, even after the elimination of repetitive sequences (Table 3). This effect is mainly due to relatively short FCs that are found only in the human sequences and not in any other species.

**Table 3:** Pairwise comparisons between paralogous clusters and the number of shared PFCs (phylogenetic footprint cliques) and their complete lengths

	*KCNA 3-2-10 clusters*											
KCNA 6-1-5 clusters		*Hs*	*Gg*	*Xt*	*Gaa*	*Ola*	*Tra*	*Tna*	*Trb*	*Tnb*	*Gab*	*Drb*
	
	Number of cliques											
	
	*Hs*	24	17	21	13	13	9	7	8	7	13	13
	*Gg*	20	12	20	9	18	9	6	15	9	15	8
	*Xt*	17	11	39	8	18	11	10	12	6	12	11
	*Tra*	3	2	3	1	2	2	1	3	1	3	2
	*Tna*	3	3	3	1	2	1	1	3	2	2	2
	*Ola*	1	2	6	1	2	1	1	3	1	2	2
	*Gaa*	3	1	2	1	2	1	1	2	1	1	1
	*Dra*	2	1	0	1	1	1	1	2	1	0	2
	*Tnb*	2	2	2	2	1	2	2	2	1	2	3
												
	
	Total length											
	
	*Hs*	1248	774	1177	564	584	407	340	644	338	830	556
	*Gg*	980	508	946	420	815	382	293	869	381	987	330
	*Xt*	801	450	3014	332	742	521	438	633	302	639	406
	*Tra*	155	444	512	33	397	89	33	214	38	163	454
	*Tna*	155	510	486	43	421	43	43	139	69	89	359
	*Ola*	136	477	667	66	441	66	66	289	60	196	427
	*Gaa*	100	58	98	99	132	99	99	114	31	31	291
	*Dra*	83	21	0	52	52	52	52	127	42	0	105
	*Tnb*	50	57	64	68	72	74	67	54	23	79	98

## Discussion

Up to now, shaker-related voltage-gated potassium channels have been mainly studied in tetrapods with a strong emphasis on functional and structural aspects [34,35,45], but not within a larger phylogenomic framework. Neither the number of genes within ray-finned fish nor the phylogenetic relationships of these genes have been studied previously. Yet, this information clearly provides useful insights for the comparison of experimental and functional studies. With prior knowledge of the existence of two 3-gene-clusters (3-2-10 and 6-1-5) in mammals [32,33], we were able identify two clusters in chicken and the frog *Xenopus tropicalis *as well. The human clusters are positioned on chromosomes (chr 1, chr 12) that have been reported to contain a number of genes duplicated during large scale duplication events [7]. The addition of new data from fish reveals the existence of four clusters in teleosts as a result of the fish-specific genome duplication (FSGD). *KCNA3-2-10 *cluster of *Tetraodon *are on chromosomes 11 and 9, in *Oryzias *on chromosomes 7 and 5. The origin of those chromosomes through the FSGD has been proposed previously [16,46]. Because of missing linkage data, similar conclusions for the *KCNA6-1-5 *clusters cannot be drawn. Due to a lack of data from lampreys and hagfish, the timing of the first cluster duplication (leading to the two-cluster situation in tetrapods) is unknown (Figure 1). Most likely their origin is the result of one of the genome duplication events during chordate/vertebrate evolution (2R) [7,47]. The two genes we identified from *Hydrolagus colliei *(spotted ratfish, Chondrichthyes) so far can unambiguously be assigned to their tetrapod orthologs (*KCNA2*, *KCNA5*). This finding implies that sharks already possesses the two KCNA clusters [39], the timing of the duplication with respect to the cyclostomes remains unclear. The *KCNA *complement of the cephalochordate *Branchiostoma floridae *shows an independent series of gene duplication, with all genes being intronless. Thus cluster formation in the vertebrate lineage occurred after the divergence with the cephalochordates. In *Ciona intestinalis*, a tunicate, only one *KCNA *sequence was found, which consisted of at least five exons, of which only four were identified unambiguously. The amino-terminus was found to be highly variable among different vertebrate genes and thus BLAST searches with invertebrate sequences yielded no hits for the amino terminus.

The phylogenetic analysis suggested *KCNA7 *as the most basal vertebrate *KCNA *gene. This gene has two exons, while the rest of the vertebrate genes are intronless (Figure 1). The phylogenetic analyses could not resolve all sistergroup relationships between the intronless *KCNA *genes (*KCNA1-6*, *10*) with high confidence. This might be due to the extreme rate difference among the various members of this gene family as well as, the rapid succession of duplication events. We propose an evolutionary scenario of two consecutive tandem duplications that formed a first cluster. Mostly likely during the 2R genome duplication, the entire cluster was duplicated leading to the situation found in tetrapods (Figure 3). This hypothesis is also supported by the sistergroup relationship found between *KCNA5 *and *10*, as well as between *KCNA1 *and *2 *genes, which are now parts of different *KCNA *clusters. For the other gene pairs, the data are not as clear, but the alternative scenario of independent tandem duplications on different chromosomes is not supported by the topology of the tree. We suppose that the reconstruction problems are caused by the fast evolution of *KCNA6 *as well as by the short basal branches that render phylogenetic reconstructions difficult. This also implies that the tandem duplications must have happened, evolutionarily speaking, only shortly before the entire cluster duplications. The origin of the *KCNA4 *gene from the *KCNA7 *gene progenitor could be the result of a large-scale duplication (1R) event followed by the loss of several genes but currently there are no synteny data available to add support this hypothesis.

The phylogenetic analyses, also with smaller datasets [see Additional files 1, 2, 3], are hampered by pronounced rate differences between paralogs and increased rates of evolution of *KCNA *paralogs in the teleost fish. Accelerated rates of evolution in some fish genes have been observed before [48-50]. Pronounced rate differences can lead to wrong topologies [51]. An accelerated rate of evolution might be due to reduced selective pressure because of gene redundancy after duplication, or, alternatively, might be due to positive Darwinian selection associated with a change in function. Yet, currently, a link between expression and the formation of heterodimers between different subtypes and their evolutionary rates is not obvious. More functional data with respect to gene expression and its regulation, as well as the formation of heteromers from more species, especially teleost fishes, could provide further insights. To date, expression data are available only for few tetrapod species such as mouse, human and chicken. Furthermore, these studies only examined expression differences in a subset of tissue types [52].

For a better understanding of the characteristics of the gene clusters, we first performed an alignment based VISTA plot analysis [53]. However, no clear conserved regions across all species included were apparent, only between the orthologous fish clusters (results not shown). For a more detailed analysis, we ran the Tracker program, which is based on an initial BlastZ algorithm [40]. We performed a complete analysis using 20 sequences and obtained 670 FC, of which 182 consist of more than two sequences. Alignments and sequence positions of the FCs are provided in the supplementary data. The percentage of FCs of the complete sequences was very low in tetrapods but the teleost clusters showed comparable values (11–34%) to the Hox clusters (10–38%, Hoegg et al, submitted) (Table 4). The lack of signal observed in Vista plots is rather a pronounced lack of elements conserved between tetrapods and fish. *Hox *genes as developmental key factors, on the other hand, are involved in many different pathways and, therefore, have more regulatory elements that are conserved over long evolutionary distances. Expression of *KCNA *genes, on the other hand, was studied so far only in adult tissues, but a more continuous expression pattern in specific tissues (e.g. brain, heart, muscle) might be expected. Since *KCNA *genes are active as homo- and heterotetramers, expressional "fine tuning" might also be accomplished through the expression of a combination of different *KCNA *genes at different stages during development and in different tissue types. The analyses of orthologous clusters revealed the expected pattern for the tetrapods and within orthologous fish clusters, i.e., more closely related organisms share more cliques, while the paralogous fish clusters share less conserved elements (Tables 1, 2). This finding again is most likely caused by faster evolution in duplicated fish clusters.

**Table 4:** Length of the sequences in different organisms, counted from the start codon of the 5'most gene to the stop of the 3'most gene or until the next gene The second column contains the complete length of FCs from on sequence, and its percentage of the complete length of the cluster.

Sequence	Total length	Bp in cliques	% of bp cliques
*Homo *3-2-10	157401	5555	3.5
*Gallus *3-2-10	54669	4149	7.6
*Xenopus *3-2-10	154969	7587	5.9
*Gasterosteus *3-2-10 a	30201	6675	22.1
*Oryzias *3-2-10 a	48863	6405	13.1
*Tetraodon *3-2-10 a	24581	4403	17.9
*Takifugu *3-2-10 a	26792	4680	17.5
*Tetraodon *3-2-10 b	23872	6137	25.7
*Takifugu *3-2-10 b	26629	6815	25.6
*Gasterosteus *3-2-10 b	30044	8186	27.2
*Danio *3-2-10 b	88550	3091	3.5

*Homo *6-1-5	237607	7497	3.2
*Gallus *6-1-5	173038	7279	4.2
*Xenopus *6-1-5	144682	7518	5.2
*Tetraodon *6-1 a	8433	2044	24.2
*Takifugu *6-1a	10347	2920	28.2
*Oryzias *6-1a	8252	2835	34.4
*Gasterosteus *6-1 a	7038	1892	26.9
*Danio *6-1 a	9756	1119	11.5
*Tetraodon *1-5 b	10689	1176	11.0

However, the comparisons between paralogous clusters of the same species showed an unexpected pattern for the tetrapods included in this study (Table 3). The phylogenetic analyses show that the clusters were duplicated before the tetrapod – ray-finned fish split (+/- 450 million years ago [54]). Using sequences that were not "masked" for repetitive sequences in the Tracker analyses as proposed in the original publication [40], leads to high numbers of FCs shared only between paralogous sequences of one tetrapod species. The teleost KCNA clusters are more compact, contain less repetitive sequences and are therefore less susceptible to false hits. For gnathostome Hox clusters, where selection is acting against repetitive sequences [55], masking can be neglected, but for other gene clusters it can be useful.

This finding might imply that the clustered structure of KCNA genes is to some extent due to common expression domains of the genes within each cluster [56], or at least that the regulatory elements are not conserved over larger evolutionary distances. Since expression data is not widely available especially not for non-mammal species, it is difficult to draw further conclusions about this testable hypothesis.

## Conclusion

The KCNA gene family underwent a series of duplications within the vertebrates leading to eight genes in tetrapods and more than 13 in teleosts. The initial gene underwent at least two tandem duplications forming two three-gene-clusters, that were most likely duplicated during a genome duplication before the divergence of the Chondrichthyes. The molecular evolutionary analysis of the *KCNA *gene clusters showed only few footprint cliques (FC) that are conserved over large evolutionary distances, while among the teleost clusters more conservation was evident. Shared regulatory elements do not seem to be the major force that keep these clusters intact and therefore do not pose a general rule for gene cluster retention.

## Methods

### Database searches

Complete clusters of *KCNA *genes were downloaded from public databases such as Genbank (*Homo sapiens*), Ensemble (*Gallus gallus, Gasterosteus aculeatus, Oryzias latipes, Danio rerio*), JGI (*Branchiostoma floridae, Xenopus tropicalis, Takifugu rubripes*), and Genoscope (*Tetraodon nigroviridis*).

### Amplification of KCNA genes

Universal primers (KCNA.uni.F270.super ATY YTN TAY TAY TAY CAR TCI GGI GG, KCNA.uni.R583.super ACN GTN GTC ATN GRI ACI GCC ACC A) were designed based on known sequences. These primers amplified all intronless *KCNA *genes (*KCNA 1, 2, 3, 4, 5, 6, 10*) in vertebrates. PCRs were performed in 25 μl reactions using 0.5 μl (1 Unit/μL) of REDTaq DNA polymerase (Sigma), 2.5 μl 10×REDTaq PCR reaction buffer, 1.5 μl dNTPs (2.5 mM each), 1.0 μl MgCl_2 _(25 mM), 1.0 μl of each primer (10 μM) and 20 ng of genomic DNA. 35 PCR cycles with an annealing temperature of 50°C and an extension time of two minutes were conducted in each experiment. PCRs were performed with DNA from *Hydrolagus colliei *(spotted ratfish, Chondrichthyes) *Polypterus senegalus *(bichir), *Acipenser baerii *(Siberian sturgeon), *Lepisosteus platyrhynchus *(Florida gar), *Amia calva *(bowfin), *Gnathonemus petersi *(elephant nose fish) and *Oreochromis niloticus *(tilapia).

PCR fragments were subcloned using the TOPO-TA cloning kit (Invitrogen). Colony PCRs were performed followed by sequencing on an ABI-Hitachi 3100 capillary sequencer following the manufacturer's instructions using the BigDye Terminator cycle-sequencing ready reaction kit (Applied Biosystems Inc.).

Sequences were assembled and checked using Sequence Navigator™1.0 (Applied Biosystems). Accession numbers and genomic locations of sequences used in this study are listed in Additional file 4.

### Phylogenetic analyses

Deduced amino acid sequences were aligned with ClustalW and manually refined in BioEdit [57]. Amino- and carboxy-terminal sequences were not alignable and therefore were excluded from further analyses. Models of sequence evolution were chosen with ProtTest [58] using the AIK criterion. Maximum likelihood analyses were performed with PhyML [59], running 500 bootstrap replicates. Bayesian inference were performed in MrBayes3.1 [60] for 100 000 generations, running 4 chains, sampling every 10^th ^tree and a burnin value of 5000.

### Cluster analyses

Complete gene clusters from *Homo sapiens*, *Gallus gallus*, *Xenopus tropicalis*, *Takifugu rubripes*, *Tetraodon nigroviridis, Gasterosteus aculeatus, Oryzias latipes *and *Danio rerio *were analyzed using the Tracker program [40] for the identification of conserved sequences. Since suspicious amounts of paralogous hit from one species were found for human and frog, we removed repetitive sequences using RepeatMasker [44]. We excluded direct comparisons between the two pufferfish sequences to avoid biased results due to their recent common ancestry. Footprint cliques with more than two sequences are given in Additional file 5. We also performed VISTA plots using LAGAN multiple alignments [53].

## Authors' contributions

SH participated in the design of the study, carried out lab work and drafted the manuscript. AM participated in the design of the study and the writing of the manuscript.

## Supplementary Material

Additional file 1 **Maximum likelihood tree of *KCNA3/6***. The dataset included 42 species of which ten were outgroup sequences (*KCNA4*) and had a total length of 378 amino acid positions. The model applied was JTT + I + G (pinv = 0.35, a = 0.61). Values in the front are bootstrap percentages as obtained from 500 bootstrap replicates. Posterior probabilities as obtained by MrBayes 3.1.1 [59](100 000 generations) are indicated with asterisks. (** = 100% PP, * = 99–95% PP) Click here for file

Additional file 2 **Maximum Likelihood tree of *KCNA1/2***. The dataset included 49 species of which ten were outgroup sequences (*KCNA4*) and had a total length of 449 amino acid positions. The model applied was JTT + I + G (pinv = 0.37, a = 0.61). Values in the front are bootstrap percentages as obtained from 500 bootstrap replicates. Posterior probabilities as obtained by MrBayes 3.1.1 [59](100 000 generations) are indicated with asterisks. (** = 100% PP, * = 99–95% PP) Click here for file

Additional file 3 **Maximum likelihood tree of *KCNA5/10***. The dataset included 39 species of which ten were outgroup sequences (*KCNA4*) and had a total length of 360 amino acid positions. The model applied was JTT + I + G (pinv = 0.42, a = 0.81). Values in the front are bootstrap percentages as obtained from 500 bootstrap replicates. Posterior probabilities as obtained by MrBayes 3.1.1 [59](100 000 generations) are indicated with asterisks. (** = 100% PP, * = 99–95% PP) Click here for file

Additional file 4 Accession numbers of nucleotide sequences, including those newly determined for this study that were analyzed in this study. Click here for file

Additional file 5 **List of all footprint cliques (FCs) with more than two sequences as obtained by Tracker**. For each clique, the relative position in regards to genes is given as well as the nucleotide position within the sequences. Click here for file
