# Supplementary figures and images for: Phylogenomic analyses of KCNA gene clusters in vertebrates: why do gene clusters stay intact?
Source: BMC Evol Biol. 2007 Aug 15;7:139. doi: 10.1186/1471-2148-7-139 (PMC1978502; doi:10.1186/1471-2148-7-139)

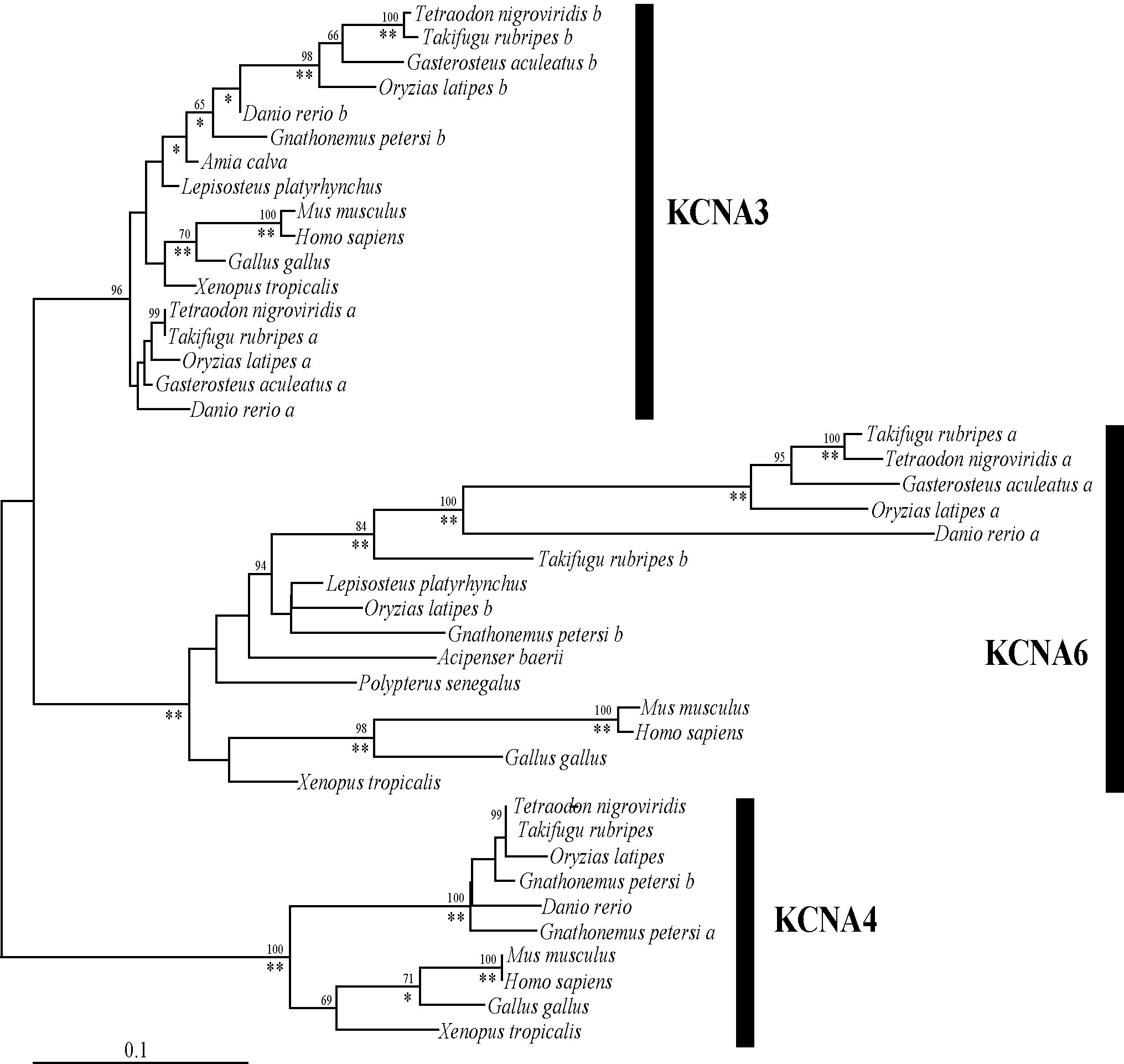

Supplement: Additional file 1 — Maximum likelihood tree of KCNA3/6. The dataset included 42 species of which ten were outgroup sequences (KCNA4) and had a total length of 378 amino acid positions. The model applied was JTT + I + G (pinv = 0.35, a = 0.61). Values in the front are bootstrap percentages as obtained from 500 bootstrap replicates. Posterior probabilities as obtained by MrBayes 3.1.1 [59](100 000 generations) are indicated with asterisks. (** = 100% PP, * = 99–95% PP) [file 1471-2148-7-139-S1.jpeg]

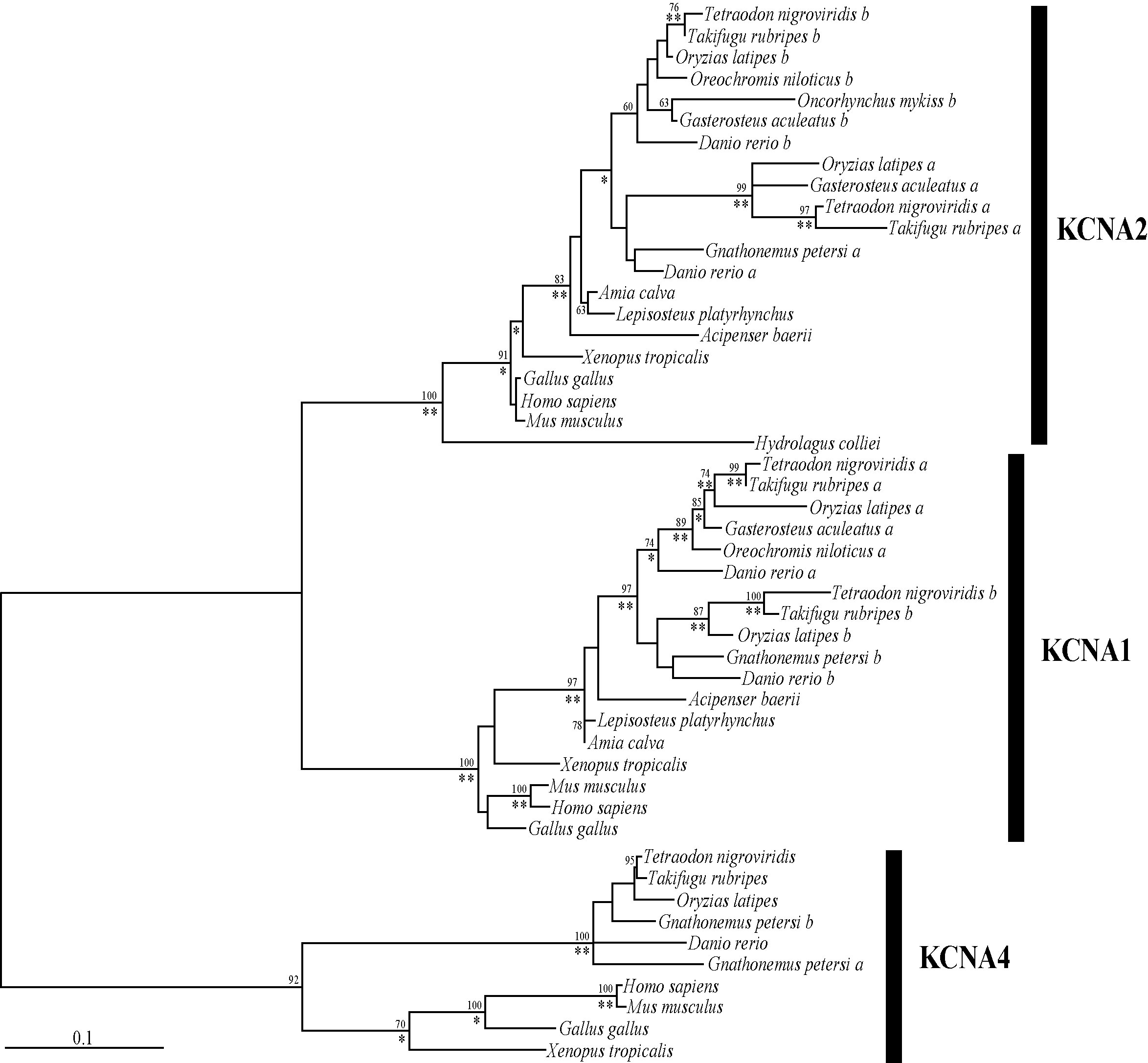

Supplement: Additional file 2 — Maximum Likelihood tree of KCNA1/2. The dataset included 49 species of which ten were outgroup sequences (KCNA4) and had a total length of 449 amino acid positions. The model applied was JTT + I + G (pinv = 0.37, a = 0.61). Values in the front are bootstrap percentages as obtained from 500 bootstrap replicates. Posterior probabilities as obtained by MrBayes 3.1.1 [59](100 000 generations) are indicated with asterisks. (** = 100% PP, * = 99–95% PP) [file 1471-2148-7-139-S2.jpeg]

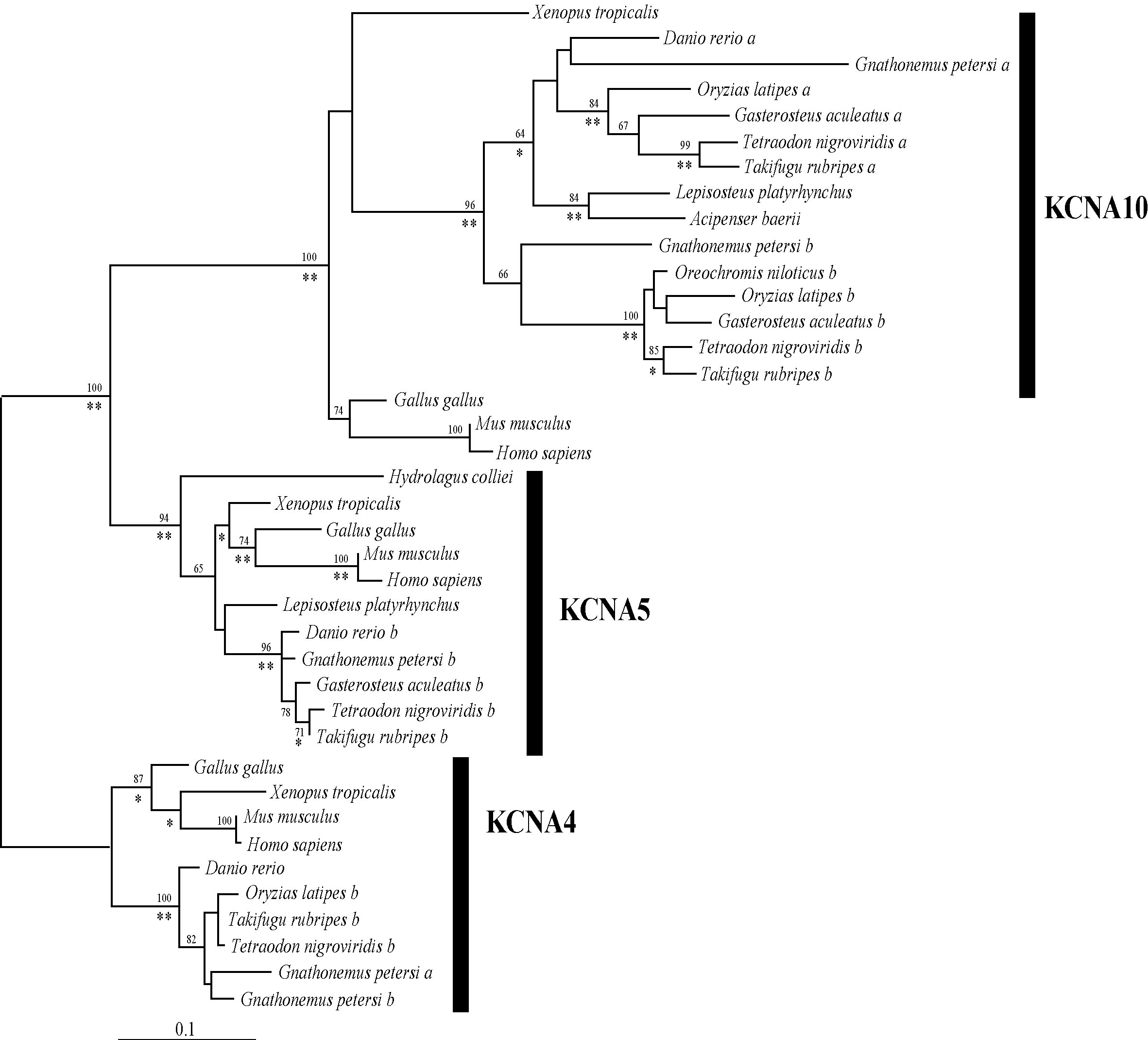

Supplement: Additional file 3 — Maximum likelihood tree of KCNA5/10. The dataset included 39 species of which ten were outgroup sequences (KCNA4) and had a total length of 360 amino acid positions. The model applied was JTT + I + G (pinv = 0.42, a = 0.81). Values in the front are bootstrap percentages as obtained from 500 bootstrap replicates. Posterior probabilities as obtained by MrBayes 3.1.1 [59](100 000 generations) are indicated with asterisks. (** = 100% PP, * = 99–95% PP) [file 1471-2148-7-139-S3.jpeg]
