# Supplementary material for: Phylogenomic analyses of KCNA gene clusters in vertebrates: why do gene clusters stay intact?
Source: BMC Evol Biol. 2007 Aug 15;7:139. doi: 10.1186/1471-2148-7-139 (PMC1978502; doi:10.1186/1471-2148-7-139)
Supplement: Additional file 4 — Accession numbers of nucleotide sequences, including those newly determined for this study that were analyzed in this study. [file 1471-2148-7-139-S4.doc]

Table S1: Accession numbers of nucleotide sequences that were analyzed in this study.

|  | ***Homo sapiens*** | ***Mus musculus*** | ***Gallus gallus (v34)*** | ***Xenopus tropicalis (v3.0)*** |
| --- | --- | --- | --- | --- |
| **KCNA6** | gi|25952089 | gi|32452041 | chr1: 185587080-185585539 | scaffold10: 1215167-1216612 |
| **KCNA1** | gi|4557684 | gi|31560569 | chr1: 185492855-185491380 | scaffold10: 1288101-1289570 |
| **KCNA5** | gi|25952086 | gi|22122428 | chr1: 185406436-185404541 | scaffold10: 1358457-1359849 |
| **KCNA3** | gi|25952081 | gi|6680515 | chr26: 164200-163003 | scaffold393: 402314-403786 |
| **KCNA2** | gi|25952079 | gi|31543023 | chr26: 142347-140848 | scaffold393: 458881-460380 |
| **KCNA10** | gi|27436997 | gi|38076943 | chr26: 111058-109530 | scaffold393: 555784 557283 |
| **KCNA4** | gi|25952084 | gi|31543025 | chr3: 44030931-44032383 | scaffold331: 184400-187000 |
| **KCNA7** | gi|25952091 | gi|6754413 |  | scaffold832: 78000-102000 (1 Intron) |
|  |  |  |  |  |
|  | ***Hydrolagus colliei*** | ***Acipenser baerii*** | ***Amia calva*** | ***Lepisosteus platyrhynchus*** |
| **KCNA6** |  | EF552391 | EF552393 | EF552392 |
| **KCNA1** |  | EF552377 | EF552378 | EF552380 |
| **KCNA5** | EF552396 |  |  | EF552397 |
| **KCNA3** |  |  | EF552388 | EF552389 |
| **KCNA2** | EF552387 | EF552382 | EF552384 | EF552383 |
| **KCNA10** |  | EF552399 |  | EF552401 |
| **KCNA4** |  |  |  |  |
| **KCNA7** |  |  |  |  |

*Polypterus senegalus* KCNA6 EF552390

|  | ***Tetraodon nigroviridis*** | ***Takifugu rubripes v3.0*** | ***Gasterosteus aculeatus (v1.0)*** | ***Oryzias latipes (v1.0)*** |
| --- | --- | --- | --- | --- |
| **KCNA6a** | chr13: 9752300-9758799 | scaffold4318: 1-2000 | groupXIX: 6035602-6037038 | chr6:ENSORLG00000003738 |
| **KCNA6b** |  | scaffold5974: 900-2500 |  | Ultracontig279: ENSORLT00000025729 |
| **KCNA1a** | chr13: 9752000-9753999 | scaffold4318: 4880-6700 | groupXIX: 6030303-6031778 | chr6:ENSORLG00000003735 |
| **KCNA1b** | chrUn: 19138000-19139849 |  |  | Ultracontig279: ENSORLT00000025728 |
| **KCNA5a** |  |  |  |  |
| **KCNA5b** | chrUn: 19146900-19148753 | scaffold3280: 16000-17900 | scaffold_439:7575:9338 |  |
| **KCNA3a** | chr9: 4744106-4745683 | scaffold54: 206200-208000 | groupXII: 12262938-12264526 | chr7:ENSORLG00000008487 |
| **KCNA3b** | chr11: 7777500-7779299 | scaffold785: 75150-76950 | groupXVII: 3447975-3449423 | HdrR_200510_scaffold1830_contig117233: 16770-18187 |
| **KCNA2a** | chr9: 4732317-4735322 | scaffold54: 194300-196150 | groupXII: 12251007-12252509 | chr7:ENSORLG00000008486 |
| **KCNA2b** | chr11: 7785600-7787799 | scaffold785: 84000-86000 | groupXVII: 3437445-3438929 | chr5:ENSORLG00000006419 |
| **KCNA10a** | chr9: 4721648-4720997 | scaffold54: 180400-182150 | groupXII: 12234307-12236019 | chr7:ENSORLG00000008479 |
| **KCNA10b** | chr11: 7799900-7801645 | scaffold785: 100200-102400 | groupXVII: 3419379-3421067 | chr5:ENSORLG00000006413 |
| **KCNA4a** | chr5: 11139300-11141799 | scaffold290: 72369-74675 |  | GENSCAN00000033005 |
| **KCNA4b** |  |  |  |  |
| **KCNA7a** |  |  |  | HdrR_200510_scaffold19 contig16462: 2922-2027  contig16463: 2374-1687  (1 Intron) |
| **KCNA7b** | chr2: 5339100-5342249 (1 Intron) | scaffold824: 33500-37000 |  | HdrR_200510_scaffold89 contig45354:  2272-6929 (1 Intron) |
|  |  |  |  |  |
|  | ***Danio rerio (Zv5)*** | ***Oreochromis niloticus*** |  | ***Gnathonemus petersii*** |
| **KCNA6a** | scaffold1486: 31895-30468 |  |  |  |
| **KCNA6b** |  |  |  | EF562458 |
| **KCNA1a** | scaffold1486: 23617-22139 | EF552379 |  |  |
| **KCNA1b** | NA8390: 11630-10149 |  |  | EF552381 |
| **KCNA5a** |  |  |  |  |
| **KCNA5b** | Chr6 10133090-10134709 |  |  | EF552398 |
| **KCNA3a** | Chr19: 64644158-64645182 |  |  |  |
| **KCNA3b** | Chr6: 21807665-21806214 |  |  | EF562460 |
| **KCNA2a** | NA4674: 94468-95955 |  |  | EF552386 |
| **KCNA2b** | Chr6: 31810420-31811876 | EF552385 |  |  |
| **KCNA10a** | Chr19: 61270244-61271950 |  |  | EF562459 |
| **KCNA10b** | Chr6: 31735930-31737615 | EF552400 |  | EF552402 |
| **KCNA4a** | Chr7: 22628554-22630032 |  |  | EF552394 |
| **KCNA4b** |  |  |  | EF552395 |
| **KCNA7a** |  |  |  |  |
| **KCNA7b** |  |  |  |  |
