# Supplementary material for: Phylogenomic analyses of KCNA gene clusters in vertebrates: why do gene clusters stay intact?
Source: BMC Evol Biol. 2007 Aug 15;7:139. doi: 10.1186/1471-2148-7-139 (PMC1978502; doi:10.1186/1471-2148-7-139)
Supplement: Additional file 5 — List of all footprint cliques (FCs) with more than two sequences as obtained by Tracker. For each clique, the relative position in regards to genes is given as well as the nucleotide position within the sequences. [file 1471-2148-7-139-S5.doc]

Footprint cliques with more than 2 sequences as obtained by Tracker

**Clique 36**:

| Oryzias latipes | KCNA3a-2a | 5925 | (42) |
| --- | --- | --- | --- |
| Takifugu rubripes | KCNA3a-2a | 1777 | (41) |
| Tetraodon nigroviridis | KCNA3a-2a | 4725 | (21) |

Ol_KCNA3210a atACAGCTGGCTCTGGCGGTGCACACCAGCGTGTGGAAGCAc

Tr_KCNA3210a gcACAGCTGGCTCCGTCGTTGCGCACCAGCGTGTGGAAGag

Tn_KCNA3210a CGCACCAGCGTGCGGAAGCAg

+++++++++++ + ++ ++* ********** *****

16 of 20 positions (0.800) totally conserved.

32 of 42 positions (0.762) conserved in aligned fragments.

**Clique 37**:

| Gasterosteus aculeatus | KCNA3a-2a | 2347 | (281) |
| --- | --- | --- | --- |
| Oryzias latipes | KCNA3a-2a | 6113 | (287) |
| Takifugu rubripes | KCNA3a-2a | 2033 | (256) |
| Tetraodon nigroviridis | KCNA3a-2a | 4923 | (259) |

GaKCNA3210a ACCTCATGTGCATTGTGCAATAGCCAATAgTTTATCACGAC------

OlKCNA3210a TCTGAGGGgctGATCCTGTTGTGCGTt-TCCAGTAGCAAG---TGTATAATCACAACAGg

TrKCNA3210a a----------GAACCTGTTGTGCGTCGTTCAATAGCCAG---TTTATCGCCACAGCAGT

TnKCNA3210a TCTGAGGG-----ACCTGTTGTGCGTCGTGCAATAGCCAATAt----------CAACAGT

~~~~~~~ ~~ *** ***** * ~* ** **** * ~~ ~ ~~~ ~*~ ~~~

GaKCNA3210a -----GTAGAAGCGTTCTAGGTGTGAAAGGTTAGTACAAATgtatGTTTTACTCGAAGAA

OlKCNA3210a tagaAATAGAAGCTTTTTTACTGTGAAGGGTGCGTAAAACTgc--GTGTTCCTTGAAGGA

TrKCNA3210a ----AGTAGAAGCTTAAAAACTGTGAAAAATTAGTGAAAAT------TCTACTCGAACTA

TnKCNA3210a ----ATTAGACGCTTAATTACTGTGAAAAATGAGTGC-AAG------TCTACTCGAAATA

~ **** ** * ****** * ** ~* ~ ~~ * ** *** *

GaKCNA3210a AACTCACATTaaa--ATGTGTTGTGAGTCGTGATCCGTCAGCCATATAGCCTGCATTCAA

OlKCNA3210a CACTTACACTgtttcATGCGTTATGCGTCGTGATCAGTCATG--CTTAGACAATGTATAA

TrKCNA3210a GACCCATTGACA-------------TGTCGTGGTTGGTCAGTCATATAGCCTGTGTA---

TnKCNA3210a GACCCATTGACA-------------CGTCGTGATTCGTTAGT--TATAGCCTGTGTATAA

** * ~~~ ~~~ ~~ ****** * ** * ~~ *** * * ~~

GaKCNA3210a TATTCAGAATCATTTCAAa---TGTGTGCGTTTAAATAC--CACATGTATtgtctgAAAA

OlKCNA3210a TAACCAG--TCATTTAAATatgTGTCTGTGTTCAAACACACGT-ATGTCTGA----AAAA

TrKCNA3210a -------GACCATTTAAATC--TGTCTGTGTTTAAACACATGTGATGTCTGA----AAAA

TnKCNA3210a TATCCAGGATCATTTAAACC--TGTCTGTGTTTTAACAC--GTGATGTCTGA----AAAA

~~ ~~~ ~ ***** ** *** ** *** ** **~ **** * ****

GaKCNA3210a CACAGTCCTC----GATATATTATCTAAATTAGATTGTTCTGTGATATGCCTTTATCGTG

OlKCNA3210a AAAAacggttcgaaGATATAAAATGTCAACCAGACCGTTCCGTGTTATGCCTTCATTATG

TrKCNA3210a CAAAGTTCTG----CATATACTAT-TCAATCAGACCTTTCTGTGCTATGCCTTTAGTGTG

TnKCNA3210a CAAAGTTCCT----CATATACTAT-CCAATTGGACCTATCTGTGCTATGCCTTTAGTGTG

* * ***** ** ** ** ** *** ******** * **

GaKCNA3210a CGTTAAtactgtcgtt

OlKCNA3210a CG

TrKCNA3210a CGTTAAAGTCgat

TnKCNA3210a CGTTCAAGTCtat

**++ +

119 of 215 positions (0.553) totally conserved.

122 of 316 positions (0.386) conserved in aligned fragments.

**Clique 39**:

| Gasterosteus aculeatus | KCNA3a-2a | 2965 | (64) |
| --- | --- | --- | --- |
| Oryzias latipes | KCNA3a-2a | 6751 | (60) |
| Takifugu rubripes | KCNA3a-2a | 2618 | (22) |
| Tetraodon nigroviridis | KCNA3a-2a | 5481 | (44) |

GaKCNA3210a GATTGCCCTtgGTCTT---AAAAAGACTGTTCGCCCACCACGCTtTGCGTGCGTAA

OlKCNA3210a gcgtGACTTCACTctc---T---AATGAGGCAGTTTTATCACCAAGCTgctc-TGCGTAA

TrKCNA3210a TCTac-AATAAAGACTGTTCGG-----------T

TnKCNA3210a TCA----GTTTTtaAAAAAAGACTGTTCGG-----------TGCGTGCGTAA

++ + + ~~ ~ ~ ~* ** * *** ~~~~~ ~~~ +~+++++++

GaKCNA3210a AA--ACAaCACCA

OlKCNA3210a Ag------CACCA

TrKCNA3210a

TnKCNA3210a AAttACA

+ ~~~ +++++

7 of 17 positions (0.412) totally conserved.

25 of 73 positions (0.342) conserved in aligned fragments.

**Clique 42**:

| Takifugu rubripes | KCNA3b-2b | 2087 | (117) |
| --- | --- | --- | --- |
| Tetraodon nigroviridis | KCNA3b-2b | 4429 | (110) |
| Gasterosteus aculeatus | KCNA3b-2b | 4331 | (116) |

TrKCNA3210b AGTTAGTGGTTGGTGGAAAATTGTATTTTTTACAGCTTTCAGTGCCACACGATGCC-TAC

TnKCNA3210b GTGGCTGGTTAAAAAGGAATATTTTTACAGCTTGTAGTGCCACACCATGCA-TAt

GaKCNA3210b AGTGAGCAGCTTGTT-AAAAGCAATATTTGTGCAGtacacg--GCCACTCGATGCtaTAC

+++ +* * * ** **** *** * *** ~~***** * **** **

TrKCNA3210b ATATCTTGACAA--AAGctacaatcTGTTGGTGTTGATGCTTTGGTTCCCCTTGTTGCAT

TnKCNA3210b ctgtccatgCGA--AAGT-------TGTTGGTGTTGATGCTTTGGTGCCC-TTGCTGtct

GaKCNA3210b ATATCTTGAtgtttAAGT-------TGTTGGTGTTGATGATTGGGATACACGTGTAGCAT

* ** *** ************** ** ** * ~ ** * *

TrKCNA3210b

TnKCNA3210b gctatc

GaKCNA3210b attgtc

+ ++

57 of 101 positions (0.564) totally conserved.

64 of 126 positions (0.508) conserved in aligned fragments.

**Clique 43**:

| Takifugu rubripes | KCNA3b-2b | 2230 | (25) |
| --- | --- | --- | --- |
| Tetraodon nigroviridis | KCNA3b-2b | 4556 | (27) |
| Gasterosteus aculeatus | KCNA3b-2b | 4464 | (26) |

TrKCNA3210b ACAAATAGATG-GTGTTTCGTGGAGA

TnKCNA3210b tgACAGATAAATG-GTGTTTCGTGGAGA

GaKCNA3210b tt--AAAAAGATGtGTGCTTCGTGGAaa

+ ~~* * * *** *** ******** *

18 of 23 positions (0.783) totally conserved.

19 of 28 positions (0.679) conserved in aligned fragments.

**Clique 45**:

| Takifugu rubripes | KCNA3b-2b | 3135 | (58) |
| --- | --- | --- | --- |
| Tetraodon nigroviridis | KCNA3b-2b | 5576 | (66) |
| Gasterosteus aculeatus | KCNA3b-2b | 5180 | (74) |

TrKCNA3210b ctccattATTTGAGTTGGCATGCCAACTAGGCCTGACGTCACCTt--CCTTTCGTCCTCT

TnKCNA3210b ATTTGAGTTGGCATGCCAACTAGGTCTGACGTCAGCc--ACCTTTAGTCCTCC

GaKCNA3210b cctcggtgtcTGAGTTGGCATGCCAACT-GGTCTGACGTCAGCTatACCTTACATCCCCT

+ + + * ******************~** ********* * ~**** *** *

TrKCNA3210b

TnKCNA3210b ACTGCACACACTTGC

GaKCNA3210b CCAGCACACATTCGC

+ +++++++ + ++

39 of 49 positions (0.796) totally conserved.

53 of 75 positions (0.707) conserved in aligned fragments.

**Clique 47**:

| Takifugu rubripes | KCNA3b-2b | 4331 | (55) |
| --- | --- | --- | --- |
| Tetraodon nigroviridis | KCNA3b-2b | 6479 | (27) |
| Gasterosteus aculeatus | KCNA3b-2b | 7712 | (55) |

TrKCNA3210b cattccaCTCCGatggcaTTTCCATGCAGTACAGTATTTGCCCTAAAACTGCAAc

TnKCNA3210b GGTACAGTATTTTCCCTGAAACTGCAA

GaKCNA3210b cgccctgCTCCGccgctcTTTTCATGTGGTACAGTATTTGCCCTAAAACTGTAAa

+ + +++++ + +++ ++++ *********** **** ****** **

23 of 27 positions (0.852) totally conserved.

38 of 55 positions (0.691) conserved in aligned fragments.

**Clique 49**:

| Takifugu rubripes | KCNA3b-2b | 5225 | (68) |
| --- | --- | --- | --- |
| Tetraodon nigroviridis | KCNA3b-2b | 7154 | (56) |
| Gasterosteus aculeatus | KCNA3b-2b | 8843 | (74) |

TrKCNA3210b TGGCATAGCTGCAATAC-ACCG--ATTGTTCTTaaAGCAAGCACTTACCTTGACTTGAA

TnKCNA3210b GTGACATCGCTGTAACACAACCG--AGTGTTCTaga-----------ACCTTGACTTGAA

GaKCNA3210b GTGACCTTGGTGTAATACAACCtgaAGTGTTCCTgcAGCAAGCACTTACCTCGACTTGAA

+** * * * ** ** **~*** * ***** ~~~~~~~~~~~**** ********

TrKCNA3210b TTTCACTCAcat

TnKCNA3210b TTTCACACA

GaKCNA3210b TTTCACTCAgccat

****** **

40 of 54 positions (0.741) totally conserved.

41 of 74 positions (0.554) conserved in aligned fragments.

**Clique 52**:

| Takifugu rubripes | KCNA3b-2b | 6104 | (49) |
| --- | --- | --- | --- |
| Tetraodon nigroviridis | KCNA3b-2b | 7967 | (53) |
| Gasterosteus aculeatus | KCNA3b-2b | 9548 | (57) |

TrKCNA3210b TTTATGGAAACCTG-TGGCTGCTGCAATGCAGTATT--GAGGTAAGGGGGTA

TnKCNA3210b tGTGTGTG-TGAAAACCTG-TGGCTGCTGCAATGCAGTAGT--GAAGTATGGGGGTA

GaKCNA3210b cGTGTTTGCTGAAGACCTatTGGCTGCTGCAATGCAGTgtcaaGGAGTAAAGAGGTt

++++ * ** * **** ****************** * *** * ***

34 of 48 positions (0.708) totally conserved.

38 of 57 positions (0.667) conserved in aligned fragments.

**Clique 53**:

| Tetraodon nigroviridis | KCNA3a-2a | 6261 | (30) |
| --- | --- | --- | --- |
| Takifugu rubripes | KCNA3b-2b | 6907 | (57) |
| Tetraodon nigroviridis | KCNA3b-2b | 8609 | (119) |
| Gasterosteus aculeatus | KCNA3b-2b | 10461 | (122) |

TnKCNA3210a GACT-TTTGCTGACTTTTGACTGTACATGAc

TrKCNA3210b GAGATT-CTCGCTGACCTTTGGCTTCTCATGAGGT

TnKCNA3210b CTATCGCTTCTCCTTTCACTCCTGGGAGATTcCCCGCTGACCTTTGGCTCCTCATGAGGT

GaKCNA3210b CCACCTCCTCCCCTTCGAATGCGAGG-GATT-CCCGCTGACCTTTGGCTCCTCTGGACGT

+ + + + ++ ++++ + + + ++~** * ****** **** ** * ** ++

TnKCNA3210a,

TrKCNA3210b, CATTTCTGtcttcGGCACTTTCa

TnKCNA3210b, CACTTCTGACCATGGCGCTTTCGCTGTCAGCTCt----TTGACAGAGAGaa-GAAGATTT

GaKCNA3210b, CGCTCCTGCCCGCGCTGTTTTGATTCCCAGCCCcggtaTTGATGGCGGGggcGAGGATTT

+ + +++ + + +++ + ++++ + ++++ + + + ++ +++++

TnKCNA3210a

TrKCNA3210b

TnKCNA3210b ATTG

GaKCNA3210b ACTG

+ ++

18 of 30 positions (0.600) totally conserved.

68 of 124 positions (0.548) conserved in aligned fragments.

**Clique 54**:

| Gasterosteus aculeatus | KCNA3a-2a | 3902 | (29) |
| --- | --- | --- | --- |
| Takifugu rubripes | KCNA3a-2a | 3666 | (28) |
| Tetraodon nigroviridis | KCNA3a-2a | 6717 | (29) |

GaKCNA3210a GTCATCAGTTAAATTGCCTACTCATTTCT

TrKCNA3210a GTCACCAGCTAAATAGCCTACTCATTCt

TnKCNA3210a GTCATCTGCTAAATGGTCTGCTCGTTTCT

**** * * ***** * ** *** ** +

19 of 28 positions (0.679) totally conserved.

20 of 29 positions (0.690) conserved in aligned fragments.

**Clique 57**:

| Gasterosteus aculeatus | KCNA3a-2a | 4903 | (35) |
| --- | --- | --- | --- |
| Takifugu rubripes | KCNA3b-2b | 5574 | (125) |
| Tetraodon nigroviridis | KCNA3b-2b | 7462 | (86) |
| Gasterosteus aculeatus | KCNA3b-2b | 9237 | (111) |

GaKCNA3210a a-------------------------------------------CTGTGTCATTG-----

TrKCNA3210b T--GAAATGCAGCTTGGTGGCATACTGCAGTGGACCAGTGTCCTTGTGTTT

TnKCNA3210b GGGTCTAtctgaGAAAGGCAGCTTGGCAGCACGCTGCAGTGGACCCGCGTCCTTGTGTTT

GaKCNA3210b GGGTGTAatT--GAGGTGCAGCTTGCCAGCATACCGCAGTGGACCAGCGTCCTTGTGTCT

~~~ ~~ ~ ~~ ~~~~~~~~ ~~~ ~ ~~~~~~~~~* * *** ***~~~ ~

GaKCNA3210a -----------------------------------------------------ACTGGCA

TrKCNA3210b CTCCTGTAGTCTGGCTCTTTTCCAACttctgccctcatccaactgaggcgggaACTGGCA

TnKCNA3210b CTCCTGTCGTCTGGCTCTTTTCCAAC

GaKCNA3210b CTCCTGGTGTCTGGCTCTTTTCCAg------------------------------TGCCG

~~~~~~ ~~~~~~~~~~~~~~~~ ~ ~~++ +

GaKCNA3210a TTTCTCTATAAAGCTt

TrKCNA3210b TCTCTCTATAATGCTc

TnKCNA3210b

GaKCNA3210b TCTCTCAAcccgctgggcctcaa

+ ++++ +

8 of 11 positions (0.727) totally conserved.

17 of 143 positions (0.119) conserved in aligned fragments.

**Clique 59**:

| Gasterosteus aculeatus | KCNA3a-2a | 4979 | (184) |
| --- | --- | --- | --- |
| Oryzias latipes | KCNA3a-2a | 8889 | (98) |
| Takifugu rubripes | KCNA3a-2a | 4986 | (209) |
| Tetraodon nigroviridis | KCNA3a-2a | 7845 | (98) |

GaKCNA3210a ctttagacaga---TATTTAAATTCTGTTTTcctttTTCTTGGTAATGATATTCATTACG

OlKCNA3210a

TrKCNA3210a ccctggacatggttTTTGTACATTCTGCATTtatagTTCTGGGTAATGATATTCATTACA

TnKCNA3210a

+ + ++++ + + ++ ++++++ ++ + ++++ ++++++++++++++++++

GaKCNA3210a GACA--------AAAACAGCTTTTGCgacaAAGTAAAAAGTTGTGACAACTGAGGCCAAC

OlKCNA3210a

TrKCNA3210a GACAtttactgaAAAACAGCTCTTGCttc-AAGTCCAAAGTGGCAAAGACTGAGATCAAC

TnKCNA3210a

++++ +++++++++ ++++ + ++++ +++++ + + ++++++ ++++

GaKCNA3210a C-GTGATTTTCTGATATGAAACTGTGATAC--AAAgCAAAGACTGTTAGCCATATGCCTT

OlKCNA3210a CTGTGATTAACTGATATGAAACTGTGATTC--AAAACAAAGACTGTTAGCCACATGCCTT

TrKCNA3210a CTGTGGTTTTCTGATATAAAACTGTGA--C--AAATGAAAGACTGTTAGCCATATGCCTT

TnKCNA3210a CTGTGATTTGCTAAAAAAAAACTGTGA--CatAAATGAAAGACTGTTAGCCATATGCCTT

*~*** ** ** * * *********~ * *** *************** *******

GaKCNA3210a TTGTTAGCTCTGTGTGCT

OlKCNA3210a TgTT-AGTTCTTTGTGCTCCGTGTTGTTGATATGTAAAACg

TrKCNA3210a TTTT-AGCTGTGTGTGTTCTGTATTACTAATATGT

TnKCNA3210a TTTTTAGCGCTGTGTATTTTGTTTTACTAATATGTAAGAC

* *~** * *** * ++ ++ + ++++++++ ++

54 of 72 positions (0.750) totally conserved.

149 of 221 positions (0.674) conserved in aligned fragments.

**Clique 61**:

| Gasterosteus aculeatus | KCNA3a-2a | 5206 | (60) |
| --- | --- | --- | --- |
| Oryzias latipes | KCNA3a-2a | 8998 | (60) |
| Takifugu rubripes | KCNA3a-2a | 5217 | (12) |

GaKCNA3210a atGTGTGCCAGTTGTAGCTTTTCCTCTGAtctgcACATGTAGAACAATAGAGAAACATGc

OlKCNA3210a acGTGTGCTAGTTGCTGCTATTCCCTTGACttatACTTTAAGAACAATACAAAGGCACGt

TrKCNA3210a TTGCCTTGACat

+ ++++++ +++++ +++ ** * *** * ++ + +++++++++ + + ++ +

7 of 12 positions (0.583) totally conserved.

39 of 60 positions (0.650) conserved in aligned fragments.

**Clique 77**:

| Gasterosteus aculeatus | KCNA3a-2a | 8317 | (124) |
| --- | --- | --- | --- |
| Oryzias latipes | KCNA3a-2a | 16002 | (90) |
| Takifugu rubripes | KCNA3a-2a | 7776 | (109) |
| Tetraodon nigroviridis | KCNA3a-2a | 10411 | (35) |

GaKCNA3210a GAGGACATATcaaatCATGAAAGCAGGGATCCTCATCAGTCTGGCTGCTCTCAaCAG-GC

OlKCNA3210a actg----------------------------------GCCTTGCTGacttttgCAA-GC

TrKCNA3210a GTGGGCATATttcttg-TGAAAGCAGCGATTATAATCAGCATCATCACTCTCAgtatTGC

TnKCNA3210a c--------------CATG--------------------------------------TGC

~ ~~~~~ ~ ~~~~~~~~~~ ~~~ ~ ~~~~~ ~ ~ ~ ~**

GaKCNA3210a AGGTAGACAACTACCAAGGTCAACCTCTAAATATAGAGGGACCCCAGAGGGAGACATGCA

OlKCNA3210a AGGTAGAGAACTTCCAAGGTCAGCCTCTAAtTACACTGGGGTCCCACAGGGAAATGTGAA

TrKCNA3210a AGTTAAATAACTCCCAAGGGCAACTgtc--ATATAAGGGGAGCCCAGcatga

TnKCNA3210a GGTTAAATAACTTCCAAGGGCAACCtt

* ** * **** ****** ** * ~~ ++ + +++ ++++ ++ + ++ +

GaKCNA3210a GAGAG

OlKCNA3210a GGGAG

TrKCNA3210a

TnKCNA3210a

+ +++

19 of 30 positions (0.633) totally conserved.

39 of 125 positions (0.312) conserved in aligned fragments.

**Clique 80**:

| Gasterosteus aculeatus | KCNA3a-2a | 10657 | (29) |
| --- | --- | --- | --- |
| Takifugu rubripes | KCNA3a-2a | 9898 | (23) |
| Tetraodon nigroviridis | KCNA3a-2a | 12466 | (28) |

GaKCNA3210a GGGTGGACGATGCTTCCCCTTTAACGCgg

TrKCNA3210a tc---GACGGCGCTTCTCCTTTAATG

TnKCNA3210a GGGTCCACGGCGCTTCTCCTTTAATGCg

~~ *** ***** ******* *++

16 of 23 positions (0.696) totally conserved.

18 of 29 positions (0.621) conserved in aligned fragments.

**Clique 81**:

| Gasterosteus aculeatus | KCNA3a-2a | 10742 | (83) |
| --- | --- | --- | --- |
| Takifugu rubripes | KCNA3a-2a | 9964 | (64) |
| Tetraodon nigroviridis | KCNA3a-2a | 12540 | (64) |
| Takifugu rubripes | KCNA3b-2b | 7454 | (57) |
| Tetraodon nigroviridis | KCNA3b-2b | 9202 | (53) |
| Gasterosteus aculeatus | KCNA3b-2b | 11041 | (53) |
| Danio rerio | KCNA3b-2b | 39838 | (55) |

GaKCNA3210a aga------------TCTTAGTATGCGCCGCGATGCCCGGATGCAGCCCACGCAcaTCAA

TrKCNA3210a GATGCTCCTCTGCAGCTCGCACATCTCAA

TnKCNA3210a GATGCTCCACTGTAGCTCGCACATCTCAA

TrKCNA3210b CCAGCAgtaTGCATCTCTTGGTATGCAAAGCAATGCTCTAATGCAGATTTG--------A

TnKCNA3210b TGCGTCTCTTGGTATGCAAAGCCGTGCACTAATGCAGATTTG--------A

GaKCNA3210b ca---------CGGCTCTTGGTATGCGCGGCGGTGCCCGAATGCAGGTTTG--------A

DrKCNA3210b CCAGGA------GTCTTTTGGTATGCAGAGCAGCGATTGACTGCAGATTTC--------A

~~ ~ ~~~ ~+ ++ ++++++ ++ * ** ** ~~ ~~~*

GaKCNA3210a CTATGGTGAATTACGGTGAAACATGAGAGACGTGG

TrKCNA3210a CTGTCGTGAACTATGGTGAAATATGAGAGATATGG

TnKCNA3210a CTATAGTGAACTATGGTGAAATATGAGAGATATGG

TrKCNA3210b CTATA

TnKCNA3210b CTATAAGCTT

GaKCNA3210b CTGTAACCTT

DrKCNA3210b TTTAACGCT

* ++ +++++++ ++++++++ +++

7 of 26 positions (0.269) totally conserved.

38 of 95 positions (0.400) conserved in aligned fragments.

**Clique 82**:

| Gasterosteus aculeatus | KCNA3a-2a | 10843 | (133) |
| --- | --- | --- | --- |
| Oryzias latipes | KCNA3a-2a | 27232 | (90) |
| Takifugu rubripes | KCNA3a-2a | 10041 | (116) |
| Tetraodon nigroviridis | KCNA3a-2a | 12613 | (88) |

GaKCNA3210a AAACAGTTGGTCGTGATGAGGTGACACATCCGTT------TGACAGActaTAGTCAAATC

OlKCNA3210a AAACAGTCAGTCGTGATGAGATGACACAGCCGCAtcagctGCACTGAT--TAGTCAAATT

TrKCNA3210a g----------C-----GAGACGACACACCCGCA------GGACATAT--TCGTTAAAAA

TnKCNA3210a TTGGTC-----GAGACGACACGCCCGCC------GGACAGAT--TCGTCAAAAA

~~~~~~ ~~*~~~~~*** ***** *** ** * * ** ***

GaKCNA3210a AGGGAAGAAGGACAGCTATGAAGGAATGGACTCGCGAGCACAAGGCGCCTTTTAAGAAGA

OlKCNA3210a AGGGGAGCAGGGCTCATAAGACGGAATAGgcg

TrKCNA3210a GGGGGAGACGGACAAACATGAAGCGGAGGATTGACGGCCGCCATGCGCGTTGCCAGGAGA

TnKCNA3210a GGCAGAGATGGACAGATATGAAGGGACGGACTGACGGCCGCCA-GCGC

* ** ** * * ** * * ++ + + + ++++ ++ ++ +++

GaKCNA3210a GACGAACtggataacatta

OlKCNA3210a

TrKCNA3210a GACAAACaaggcagaacta

TnKCNA3210a

+++ +++ + + + ++

32 of 68 positions (0.471) totally conserved.

59 of 139 positions (0.424) conserved in aligned fragments.

**Clique 83**:

| Gasterosteus aculeatus | KCNA3a-2a | 11050 | (99) |
| --- | --- | --- | --- |
| Oryzias latipes | KCNA3a-2a | 27457 | (111) |
| Tetraodon nigroviridis | KCNA3a-2a | 12800 | (85) |

GaKCNA3210a cGCTATGCTGGTGTGTTAAGTGCATGGAGTGTGTTATGGCTcctATCCATGGTCCACACA

OlKCNA3210a tGCTATGCTGCTTTGTTTAGTGCATGGACTGTGTTGTGGCTgca---CATAGTCCACACA

TnKCNA3210a GCTGTCCTGCTTTtc-TAGTG----------------------ATTCGTTGATCGGATC

*** * *** * * ~ ****~~~~~~ ~~~~~~ ~~~~~ ~ ~~ * * * * *

GaKCNA3210a GGAGAGGACCGCAAATGGC---------------ACCTGCa-CTCCAAACTAACg

OlKCNA3210a GTAAGGGGCTGCAAATGGCttgagcatgttTTGCACCTGCTGTTTCAAACTATC

TnKCNA3210a AGCGCGGCGCGTTAAcggacgcgcc-----TGGCACCTGCCGCTCCAAACCAAC

** * ** ** ~ ~ ~~****** ~ * ***** * *

39 of 71 positions (0.549) totally conserved.

39 of 115 positions (0.339) conserved in aligned fragments.

**Clique 85**:

| Homo sapiens | KCNA6-1 | 36339 | (96) |
| --- | --- | --- | --- |
| Gallus gallus | KCNA6-1 | 34844 | (69) |
| Homo sapiens | KCNA3-2 | 3036 | (227) |
| Gallus gallus | KCNA3-2 | 8044 | (218) |
| Xenopus tropicalis | KCNA3-2 | 2964 | (158) |
| Gasterosteus aculeatus | KCNA3a-2a | 2684 | (254) |
| Oryzias latipes | KCNA3a-2a | 6466 | (258) |
| Takifugu rubripes | KCNA3a-2a | 2359 | (235) |
| Tetraodon nigroviridis | KCNA3a-2a | 5288 | (174) |
| Takifugu rubripes | KCNA3b-2b | 2560 | (68) |
| Tetraodon nigroviridis | KCNA3b-2b | 4961 | (83) |
| Gasterosteus aculeatus | KCNA3b-2b | 4893 | (90) |
| Danio rerio | KCNA3b-2b | 7286 | (123) |

HsKCNA615 GGTTTGTGGAGAGC----CCAGCtca-----------------------------

GgKCNA615

HsKCNA3210 t-----------------------------------------TCCTAATGCATCTGAGAG

GgKCNA3210 tttgcacc-----------------------------------------GCGGCTGGGAG

XtKCNA3210 cc----------------------------------------------------------

GaKCNA3210a ACGGCACCATATAAACACGGGATGACGCCGTCTGTCAGTGTCTCGTCATCGGTGCCAGAc

OlKCNA3210a ACGGCGCTTTGTAAACACCGGATCCAGCCAATTGTCAGCATCTCCTTATCTGTGCAAGAA

TrKCNA3210a aaaca--------GAGAGG----CCAGCCGACTGTCGGCGTCTCATTATCGGTGCA----

TnKCNA3210a acag--------------------------------------------------------

TrKCNA3210b

TnKCNA3210b a-----------------------------------------------------------

GaKCNA3210b t-----------------------------------------------------------

DrKCNA3210b atca--------------------------------------------------------

~ ~ ~ ~ ~~~~ ~~ ~~~~ ~ ~~~~ ~ ~~ ~~

HsKCNA615 ------------------------------------------------------------

GgKCNA615 AAG-------------------------------GA

HsKCNA3210 ATAAGCTTCGGC----AGTATCACAAG-------------------------------AA

GgKCNA3210 AGATGCAGCTGCtcatgg-----CAAG-------------------------------GA

XtKCNA3210 -------------------------AG-------------------------------AA

GaKCNA3210a gcaccttaaaagcgggAGAAGC-----GCAGCCTTCTTTCACGCACATGCAGCAATCAAA

OlKCNA3210a ACACGTGAGCGC----AGAAGCACAAaGCTGCCTCCGTACGCACACAAACAATGATCAAA

TrKCNA3210a -CACGTGAGacagagagaca--------CAGCGCTCATTCACGCACAAGCAGCGATCAAA

TnKCNA3210a --------------------------------GCGCATTCACGCACACGCGGTGATCAAA

TrKCNA3210b

TnKCNA3210b ------------------------------------------------------------

GaKCNA3210b ------------------------------------------------------------

DrKCNA3210b ------------------------------------------------------------

~ ~ ~~~~~ ~~ ~~ ~ ~ ~ ~ ~~~~ ~ ~~~~ ~

HsKCNA615 ------------------------------------------------------------

GgKCNA615 t-----------------------------------------------------------

HsKCNA3210 GATTAAAGTGGcagaCACCCCTTCCAGCGGAAGTTACTAATTCGGACCTGACTGATGCAG

GgKCNA3210 GATTGAAGTGGTGAGCACGCCTTCCAGCGGAAGTCACTAATTCGGACCGGAGTGATGCAG

XtKCNA3210 GATTAAACTGCTGGACTTGCTTTTCATCGGAAGTTACTAATTCTGACCCCAGTGATGCAG

GaKCNA3210a GATG--------GAGCT---TCCTCTTTGGATGCCTCAAATATAACCCGCTGTGATGCAG

OlKCNA3210a GATG--------GAGAGTGATTCTCTTTGGATGCCTCAAATATAGCTCTCTGTGATGCAG

TrKCNA3210a GATG--------GAGCGTGCTTCTCCTCGGATTTCTCAAATATAGCCTGCTGTGAGGCAA

TnKCNA3210a GATG--------GAGCGTGCTTCTCTTCGGATGTCTCAAATATAGCCCGCTGTGAGGCAA

TrKCNA3210b GTTTCGCCTCGTG----------AACACGCCCTGTGATTCAA

TnKCNA3210b ----------------T---TCCGCCTCGTG----------AAAACGCCCTGTGATTCAA

GaKCNA3210b -------------------------------------------CACGCCTTGTGATGCAG

DrKCNA3210b ----------------------CGCCTCGagtc------------CGCCTTGTGATGCAG

~~ ~~ ~~ ~ ~ ~ ~~~ ~~~ ~~

HsKCNA615 ---------------------CCAGGGA-----------------------------CTC

GgKCNA615 ------------------------------------------------------------

HsKCNA3210 TTTCCATAGCAACCCATGTTTCCTGGGAAACCC-GAAAAAGGTTGTCATGGCATC-TCTT

GgKCNA3210 TTGCTATGGCGACCCTGGTTTCCTGGGAAACCCC--AAAAGGTTGTCATGGCATC-CCTT

XtKCNA3210 TTTCCATAGCAACAAAGGTTTCCTGGGAAACCCTATT---CGTTGTCATGGTATC-TCTT

GaKCNA3210a TCGTCATGGTGACGGTGGTTTCCACGATAACCCCAGTCACCGTTGCCATGGCAGC-C---

OlKCNA3210a TCACTATGGTGACGGTCGTTTCCTGGGTAACCCCAGTCACCGTTGCCATGGCACC-C---

TrKCNA3210a TCTCCATGGTGACGCCGGTTTCCTGGGTAACCC-GGTCACAGTTGCCATGGCATC-C---

TnKCNA3210a TCTCCATGGTGACGCCGGTTTCCTGGGTAACCCCGGTCACAGTTGCCATGGCATC-C---

TrKCNA3210b GCACCATGACCACGTGGGTTTCCTGGGTAACTGCAT

TnKCNA3210b TCTCCATGACAACGATTGTTGCCTGGGTAACTGCATCATTAGTTAa----------TGTT

GaKCNA3210b TCTCCATGACAACGGTGGTTTCCTGGGCAACCCCCTCTGACGTTACCACGGTCTtgTGTT

DrKCNA3210b TCTCCATGGCGACTACAGTTTCCCCGGTAACCCCCGTTAATGTTACCATGACATC-C---

~~ ~~ ~~~ ~~ ~ ~~~ ~~~ ~~ ~ ~

HsKCNA615 T----------------------------GGTTGCCGTAGCAACCTT-CCAGACTGTTCT

GgKCNA615 ----------------------------CTGTTTCCGTAACAATCTT-GCAGACTGTT-T

HsKCNA3210 gCTCTCTAGCCCCACCTCCCAGCCCCTGCCGTTTCCACAGTAACCTTTCCAGATGGTTCC

GgKCNA3210 -CTCCCATCCCCCCCCCCTCGGCCCTCACCCTTCCCTCcaaAGCCCTTCCAGATGGTTC

XtKCNA3210 T----------------------------AGTTCCCATAGCAACTAT-CCAGATGGCTCC

GaKCNA3210a T----------------------------TGTTTCCACAGTAACCTT-GCAGGTGGCTCG

OlKCNA3210a T----------------------------TGTTTCCACAGTAACCTC-CCAGATGGCTTG

TrKCNA3210a T----------------------------AGTTTCCACAGTAACCTC-CCAGACGGCCTG

TnKCNA3210a T----------------------------AGTTTCCACAGTAACCTC-CCAGATGGCCTG

TrKCNA3210b

TnKCNA3210b T

GaKCNA3210b T----------------------------TATTTTCACAt

DrKCNA3210b Tccc------------------------CCGTTGTCAGAGCAACGCT-CCAGATGGTTCa

~ ~ ~ ~~~~ ~~ ~ ~ ~~~~ ~ ++ + + ~ +++ +

HsKCNA615 CTCTATCTTCTGCTTTGGGCATCAAGGGAGACC

GgKCNA615 CTCTACCCTGTGACTCTGGCTACAAGCAAAAGC

HsKCNA3210 TACTT

GgKCNA3210

XtKCNA3210 AACTT

GaKCNA3210a GAA

OlKCNA3210a GAA

TrKCNA3210a GA

TnKCNA3210a aaa

TrKCNA3210b

TnKCNA3210b

GaKCNA3210b

DrKCNA3210b ca

+ + + ++ + +++ ++++ + + +

No position present in all sequences.

24 of 333 positions (0.072) conserved in aligned fragments.

**Clique 87**:

| Gallus gallus | KCNA3-2 | 9594 | (49) |
| --- | --- | --- | --- |
| Gasterosteus aculeatus | KCNA3a-2a | 8823 | (86) |
| Oryzias latipes | KCNA3a-2a | 17069 | (82) |
| Tetraodon nigroviridis | KCNA3a-2a | 10904 | (71) |

GgKCNA3210 CTCCcc------------------------------CCTCCT-------

GaKCNA3210a tca-ATTATGTCTg----------------------TTTCTCACGCTCATCacccgcctc

OlKCNA3210a CTTCACTCTTTCTCCTTCTtgttccctctctcCATCTTTCTCTCGCTCATGCA-------

TnKCNA3210a CTTCt---TTTCTCCTTCTg------------CATCCTACTCACACACCTCCT-------

~ ~ + +** ~ ~~ ~~~~ ~ ~~~ ~ ~ * *

GgKCNA3210 ---------------C----GCTGAGtttCCTTGCAGT-----ATTTTTGCTGTGgcagc

GaKCNA3210a tcactcccacattttCTGCTGCAGAGAAAT-----GGgtggagACTTTAGGTGT

OlKCNA3210a ---------------CTGTTGCAGAGAAAG-----GGT-----ATTTTGGGTGT

TnKCNA3210a ---------------Ccttca--------CCTTGCGGT-----GTTTCTGCTGTGtcgac

* ~ ~~~ ~~~~~ * ** * ***+ + +

GgKCNA3210 t

GaKCNA3210a

OlKCNA3210a

TnKCNA3210a a

12 of 26 positions (0.462) totally conserved.

17 of 121 positions (0.140) conserved in aligned fragments.

**Clique 88**:

| Gallus gallus | KCNA3-2 | 11698 | (28) |
| --- | --- | --- | --- |
| Gasterosteus aculeatus | KCNA3a-2a | 3814 | (62) |
| Takifugu rubripes | KCNA3a-2a | 3592 | (48) |
| Tetraodon nigroviridis | KCNA3a-2a | 6638 | (53) |

GgKCNA3210 AGAGAGCAAACTGCATTTTTTGCTCATg

GaKCNA3210a ACAGAATAAACTGCATTTATTGATTATTCCTTTCCCTTTTGACCCCTGATCTTtAGTGCA

TrKCNA3210a C-TTTATAGATTATTGCTTTCCATTTTAACCCCTGACCTTGACTTCA

TnKCNA3210a g--------ACTGC-TTTATAGGTTATTGCTTTCTTTTTTGACCCCTGACCTTGACGTCt

~~~ ~~++++*~*** * * * ** +++++ ++++ ++++++++ +++ + +

GgKCNA3210

GaKCNA3210a TA

TrKCNA3210a GA

TnKCNA3210a ta

+

9 of 14 positions (0.643) totally conserved.

36 of 62 positions (0.581) conserved in aligned fragments.

**Clique 89**:

| Xenopus tropicalis | KCNA3-2 | 26924 | (34) |
| --- | --- | --- | --- |
| Gasterosteus aculeatus | KCNA3a-2a | 3763 | (38) |
| Tetraodon nigroviridis | KCNA3a-2a | 6592 | (30) |

XtKCNA3210 ctctGGTAATCTTATTATCTGCCTTA----AAAGGAAc

GaKCNA3210a ctAGGGCAGTCTCATTATCTGGCTTACCCgAAAGGAAa

TnKCNA3210a AGAGCAGTCTCATTAGCTGGTTAACCCcta

++ * * *** **** *** * *~~~ *+++++

15 of 26 positions (0.577) totally conserved.

22 of 38 positions (0.579) conserved in aligned fragments.

**Clique 98**:

| Homo sapiens | KCNA6-1 | 42059 | (25) |
| --- | --- | --- | --- |
| Homo sapiens | KCNA3-2 | 16459 | (40) |
| Gasterosteus aculeatus | KCNA3a-2a | 5354 | (149) |
| Oryzias latipes | KCNA3a-2a | 9083 | (146) |
| Takifugu rubripes | KCNA3a-2a | 5281 | (145) |
| Tetraodon nigroviridis | KCNA3a-2a | 8011 | (126) |
| Takifugu rubripes | KCNA3b-2b | 6571 | (253) |
| Tetraodon nigroviridis | KCNA3b-2b | 8350 | (203) |
| Gasterosteus aculeatus | KCNA3b-2b | 10175 | (247) |
| Danio rerio | KCNA3b-2b | 34272 | (73) |

HsKCNA615 c-----------------------------------------------------------

HsKCNA3210 g-----------------------------------------------------------

GaKCNA3210a a---CC----------TGTGAGT------AGCTCCCTTT----------------TTTCC

OlKCNA3210a cat------------TTGTGAAT------TTCTCTCTTC----------------TGTTC

TrKCNA3210a catgCC----------TGTGAAT------GACGCGCTTG----------------TATTC

TnKCNA3210a TCGCTGG----------------TATCC

TrKCNA3210b ATCCAGAGAGCTGG----------AACCCTTCTCTTTTAATTAAAGCATTTCTGCTAATG

TnKCNA3210b

GaKCNA3210b ac----AGTGCTGG----------GACCCTTTGCCTTTAATTTAACCCGACCATCAAATA

DrKCNA3210b ACCCAAAGCACGGGaTTTTGAActGACCCCTCGGTTTTAATTAAAGAGTGCCAGCTAATG

~~ ~ ~~ ~~ ~~~ ~~~~ ~ ~~~ ~~ ~ ~

HsKCNA615 --------------------------------------------TTGGCACGGGCCAG--

HsKCNA3210 ------------------------------------------------------------

GaKCNA3210a C--AGA-------------------GTTCACTagtGTGTTGTTTTGGGGATGGGGGAG--

OlKCNA3210a C--AGA-------------------CTGCACTC--TTG--ATTTTTGGAACGGGGCAG--

TrKCNA3210a C--AAG-------------------GTGCACTG--GTGTTATTGCTGGGATGGGGCATC-

TnKCNA3210a C--AGG-------------------GTGCACTG--GTGTTATTGTTGGGATGGGCCAAC-

TrKCNA3210b CTAACTGAGCTGAGCCTTATAGAGCCTGCATTA--TTG--GTCTTTGTAATGGGGCCCCG

TnKCNA3210b CTAAGTGAGCCGAGCCTTACCGGGCCGGCGTTA--TT---GTCTTTGTAACGGG-TCCTG

GaKCNA3210b C--AGTGAACTGAGCTTTAa--GTCCTGCATTAaa-T---GTGTTTATAACGGC-CCCCG

DrKCNA3210b C--AAG------------------------------------------------------

~~~~ ~~ ~ ~~~~ ~~~ ~ ~ ~ ~ ~~~~ ~ ~ ~~ ~

HsKCNA615 -------ATTGCTGTCt

HsKCNA3210 ---------------------------------TGTTAGTACTTACTACATGTTTTAATA

GaKCNA3210a -------AATAATGTC-AGGGGAAAGTTGTGTTTGCCAGTATTTGTTCTGTGTTATTCTG

OlKCNA3210a -------ATTGTTGTC-AGGGTAAGGCTGTGTATGCCAGTATTTGTTCCGTGTTATTACA

TrKCNA3210a ----------TTTGTC-AGGGcagtggcta-CAGACCTGTATTTGTTCTGTGTTCTGGTG

TnKCNA3210a ----------GCTGTCaAGGGAGCAGCTGTTCAGACAAGTATTTGTGCTGTGTTATGATG

TrKCNA3210b GCTCATAAATAATG------------------------------------------GCTG

TnKCNA3210b GCTCATTAATAATG------------------------------------------ACTG

GaKCNA3210b GCTCATAAATAATG------------------------------------------GCCA

DrKCNA3210b ------------------------------------------------------------

~~~~~~ ~ ~ ~~~~ ~~~~ ~ ~~~ ~~ ~~~~ ~

HsKCNA615

HsKCNA3210 TTCTATCTTA--------------------------------------------------

GaKCNA3210a TGCTGTCTTA--------------------------------------------------

OlKCNA3210a TGCCATCTTA--------------------------------------------------

TrKCNA3210a TGCTGTCTTA--------------------------------------------------

TnKCNA3210a TACTGTCATA--------------------------------------------------

TrKCNA3210b TGCTGTCATGAGGAACCtGGGAAAGCCATTAAAAGCTAGTGAGAGGTACCCTCATTTGTG

TnKCNA3210b TACTGTCATGAGGAACCCAGGAAAACCATTAGGAGATAGTGAGACGTATCCTCGTGTGTG

GaKCNA3210b TGCTGCCATGAGGGAGTGGGGAAAGCCAccgAAAGACATTGAGGGGCATCCGCATGAGTG

DrKCNA3210b ------------------------------------------------------------

~ ~ ~ ~ ~~~ ~ ~~~~~ ~~~ ~~ ~ ~~~~ ~ ~ ~~ ~ ~ ~~~

HsKCNA615

HsKCNA3210 -------------At

GaKCNA3210a -------------AACATGTCCGACATGAAGAGACTct

OlKCNA3210a -------------AACATGACCCATGTGAAGAGATTGt

TrKCNA3210a -------------AACATGACTCATATAAAGAGATc

TnKCNA3210a -------------AACATGACCCGTATAAAG

TrKCNA3210b GCAGCAAGCA-GTATCCTGACAGACGGAACGAGGCTGGAAGCCTGGAAGGCCTCGGGTCT

TnKCNA3210b ACAGCAGGCA-GCAACCTGACAGAAGGGACGAGGCTGGAAGCCTGGATGGCTTCGGGTCT

GaKCNA3210b GCAGCAGAcggGCAGACGGACGGAAGGGACCAGGCGGGGAGGCGTGGTGGTCTCGTGTCT

DrKCNA3210b -----------------------------------TGGAAGtgc

~~~~~ ~ ~ ~ ~ ~ ~ ~~ ++ + ++ +++ ++++

HsKCNA615

HsKCNA3210

GaKCNA3210a

OlKCNA3210a

TrKCNA3210a

TnKCNA3210a

TrKCNA3210b GACAGTAGTA

TnKCNA3210b GACAGGAGTAAc

GaKCNA3210b GCCAGGAGTAAt

DrKCNA3210b

+ +++ +++++

No position present in all sequences.

21 of 312 positions (0.067) conserved in aligned fragments.

**Clique 101**:

| Homo sapiens | KCNA6-1 | 44036 | (70) |
| --- | --- | --- | --- |
| Gasterosteus aculeatus | KCNA3a-2a | 6777 | (46) |
| Danio rerio | KCNA3b-2b | 13741 | (86) |

HsKCNA615 gagcCACATGGGACACAACATTAAGAAATACTGGAATATTTcacaca----CACACATct

GaKCNA3210a TAGTGTTTATGTGCACAT--

DrKCNA3210b aattCACATACTTCACATGATTTAGAAACACTATAATATTTAGTATTTTTGCATACAT--

+ +++++ ++++ +++ +++++ +++ ++++++* ~ ~~ ****

HsKCNA615 gaAGTTTTGGTTTc

GaKCNA3210a --GACTTGTGAGAATGTGGGCATGtgcgtc

DrKCNA3210b --AGTATTTGTTTATGTGGGAATGcgtgta

* * ++++++ +++ + ++

7 of 26 positions (0.269) totally conserved.

46 of 90 positions (0.511) conserved in aligned fragments.

**Clique 102**:

| Xenopus tropicalis | KCNA6-1 | 6167 | (31) |
| --- | --- | --- | --- |
| Oryzias latipes | KCNA3a-2a | 8666 | (67) |
| Takifugu rubripes | KCNA3a-2a | 4841 | (81) |

XtKCNA615 AAACAAACACTGAAAGCTTTGTAGCAACAGg

OlKCNA3210a TTTGTAGCCGCAGCTTctattgttaAGCTGACACAATCTGAAT

TrKCNA3210a AAACCCTCACTGACAGTATTGTAGCAGCAGCTTtgtctgg--AGCTGACACCTTGTGAGT

++++ ++++++ ++ ******* *** ++ ++ +++++++++ + +++ +

XtKCNA615

OlKCNA3210a GGGGGAGTCACGGGATCAAAagtg

TrKCNA3210a TTAGCCATCACGAGGCAAAAgag

+ +++++ + +++

10 of 14 positions (0.714) totally conserved.

50 of 84 positions (0.595) conserved in aligned fragments.

**Clique 112**:

| Takifugu rubripes | KCNA6a-1a | 248 | (42) |
| --- | --- | --- | --- |
| Oryzias latipes | KCNA6a-1a | 11509 | (52) |
| Tetraodon nigroviridis | KCNA6a-1a | 1713 | (31) |
| Gasterosteus aculeatus | KCNA6a-1a | 902 | (56) |

TrKCNA615a CAC--CAAGGAGGTGAGTGACGGA-TTTACCCACGGAGGGGTAA

OlKCNA615a GTTAa--ACAGA-TTTACCCTTGGAAGGGGTA

TnKCNA615a GGCGA------GGAGACcgagCACGGAGGCGAGTGACGGgtTTTACCCTCGCAGGGGT

GaKCNA615a GGCAAaaggggGGAGACACtg--AGGAGGTGAGTGACGGA-TTTACCCTCGGAGGGGTA

+++ + ++++++ ~~~ ++++* * ~~** * ******* * * *** +

TrKCNA615a A

TnKCNA615a

OlKCNA615a Aa

GaKCNA615a

+

17 of 27 positions (0.630) totally conserved.

33 of 62 positions (0.532) conserved in aligned fragments.

**Clique 117**:

| Takifugu rubripes | KCNA6a-1a | 2306 | (64) |
| --- | --- | --- | --- |
| Oryzias latipes | KCNA6a-1a | 13610 | (45) |
| Tetraodon nigroviridis | KCNA6a-1a | 4243 | (49) |
| Gasterosteus aculeatus | KCNA6a-1a | 3101 | (48) |
| Xenopus tropicalis | KCNA3-2 | 11556 | (35) |

TrKCNA615a tctgcctCTGACCAGATGAGCTCTGAGCGCCACTCTGTGGTCGATCCGAACCCCTTCGAA

TnKCNA615a GAGCCGTGAGCGCCACTCCGTGGCTGACCGAGATCCCTCCcAA

OlKCNA615a g---------------TGATCTCTGAGCGCCGCTTTGTGGATGATTGATACCTCTGCAGA

GaKCNA615a a--------------ATGAGCACTGAGCGCCACTCTGTGGAGGATAAACATCCCTGCAGA

XtKCNA3210 tgtacacCTGACCAGATAGGCTTTAAGTGTTACTa

~ ~ ~~~~~~~~~+ * * ** * ** ++++ ++ + + ++ + +

TrKCNA615a CAGg

TnKCNA615a Cg

OlKCNA615a CAGc

GaKCNA615a CA

XtKCNA3210

+ +

7 of 18 positions (0.389) totally conserved.

22 of 64 positions (0.344) conserved in aligned fragments.

**Clique 120**:

| Takifugu rubripes | KCNA6a-1a | 2860 | (36) |
| --- | --- | --- | --- |
| Tetraodon nigroviridis | KCNA6a-1a | 14085 | (41) |
| Gasterosteus aculeatus | KCNA6a-1a | 3795 | (35) |
| Homo sapiens | KCNA3-2 | 4878 | (27) |

TrKCNA615a TATGTGAACTTAACTTAATCCATTTTATAAATAAAT

TnKCNA615a TATGTGAACTTAACTTAATCCGTTTTATAAATAAATTATGA

GaKCNA615a TATGTGAACTTAACTTAAT-CGTTTTATAAATAAAa

HsKCNA3210 TTGATTAGTTCTATAAGTAAATTATGG

++++++++++++++** ** ** ***** **** ++++

15 of 21 positions (0.714) totally conserved.

33 of 41 positions (0.805) conserved in aligned fragments.

**Clique 121**:

| Takifugu rubripes | KCNA6a-1a | 3327 | (32) |
| --- | --- | --- | --- |
| Oryzias latipes | KCNA6a-1a | 5148 | (25) |
| Gasterosteus aculeatus | KCNA6a-1a | 4208 | (34) |

TrKCNA615a TGCGCTGTCCCCGGTGCTGAAA---ACGATCCtcg

OlKCNA615a cCTTCGCTGTCCGTGGTGCTGAAAg

GaKCNA615a tCTGCGCTGTCCGGGGTGCTGAAActgACCTTCC

+* ******** ********** ++ +++

19 of 22 positions (0.864) totally conserved.

25 of 37 positions (0.676) conserved in aligned fragments.

**Clique 128**:

| Takifugu rubripes | KCNA3b-2b | 8074 | (22) |
| --- | --- | --- | --- |
| Tetraodon nigroviridis | KCNA3b-2b | 9671 | (16) |
| Gasterosteus aculeatus | KCNA3b-2b | 11834 | (22) |

TrKCNA3210b tgCCAATCTCCATCCCACGACA

TnKCNA3210b TCTCCATCCCACGACA

GaKCNA3210b tcCCAATCGCCATCCAACGGCA

+ ++++** ****** *** **

13 of 16 positions (0.812) totally conserved.

18 of 22 positions (0.818) conserved in aligned fragments.

**Clique 129**:

| Takifugu rubripes | KCNA3b-2b | 8104 | (89) |
| --- | --- | --- | --- |
| Tetraodon nigroviridis | KCNA3b-2b | 9696 | (93) |
| Gasterosteus aculeatus | KCNA3b-2b | 11859 | (95) |
| Danio rerio | KCNA3b-2b | 41688 | (33) |

TrKCNA3210b gGAGGAAAGGAGTCGGGGAGGGGgAAGCGAGGACACACCCGCATATACT-----GTACCC

TnKCNA3210b GAGGAAAGGAATCGGGGAGGGGAAAGCGAGGACACACCCGCACATACTgtactGTACCC

GaKCNA3210b atAGGAAAGGAGTCTGGGAGGGGAGAGCGAGGACACACCCGCACGTACT-----GTACCC

DrKCNA3210b GGGTAGA-CGAGGACACACCCGTAC--ACT-----GTACCt

+++++++++ ++ ++++*** *~************** * ~*** *****

TrKCNA3210b GGTGTGC------AGGATGCTGCAGACAACATGAGGTGAA

TnKCNA3210b GGTGTGC------AGGATGCTGCAGACAACATGAGGTGAA

GaKCNA3210b GGTGTacacggcgAGGATGCCGCAGACAACATGAGGTGAA

DrKCNA3210b

+++++ + +++++++ +++++++++++++++++++

27 of 33 positions (0.818) totally conserved.

74 of 100 positions (0.740) conserved in aligned fragments.

**Clique 130**:

| Takifugu rubripes | KCNA3b-2b | 8690 | (151) |
| --- | --- | --- | --- |
| Tetraodon nigroviridis | KCNA3b-2b | 10297 | (154) |
| Gasterosteus aculeatus | KCNA3b-2b | 12516 | (156) |

TrKCNA3210b GTGCTTTTCTGTGATACTGCAGACAGGATGCTGTCTGGATGAGTTCAGTGCCTGCTGCAT

TnKCNA3210b GTGCTTTCCTATGATACTGCAGACAGGATGCTGTCTGGATGAGTTCAGTGCCTGCTGCAT

GaKCNA3210b GTGGATTTCTATGATATTCTAGACAGGATGTTGTCTatatggacTCGGTGCTTGCTGCGT

*** ** ** ***** * ********** ***** *** ** **** ****** *

TrKCNA3210b TGCCATATTCATGCATGATTTCACTGC--AAAGGGTAGTCCCATTCATTTCAGTGGATAC

TnKCNA3210b TGCCATATTCATGCATGGTTTCACGGC--AAAGGGTAGTCCCACTCATTTTAGTGGATAC

GaKCNA3210b TGCCGTATTCATGCAGGGCTCTACTGCagAAAAAGTAGTCCCGATCATTTAAGTGGATtt

**** ********** * * ** ** *** ******** ****** *******

TrKCNA3210b TGTTCATTGCAGCAGATCATTGTGCTAATTTAT

TnKCNA3210b TGTTCATCGCAGCAGATGAGTGTGCTAATTTATgag

GaKCNA3210b TGTCCATTGCAGCAGACCGGTGTCCTAATTTAcaag

*** *** ******** *** ******** ++

113 of 151 positions (0.748) totally conserved.

115 of 156 positions (0.737) conserved in aligned fragments.

**Clique 131**:

| Takifugu rubripes | KCNA3b-2b | 8899 | (25) |
| --- | --- | --- | --- |
| Tetraodon nigroviridis | KCNA3b-2b | 10467 | (81) |
| Gasterosteus aculeatus | KCNA3b-2b | 12691 | (83) |

TrKCNA3210b CAGAGTTTATT

TnKCNA3210b gacatttagccgtta--TTACTGCATTGAAATAATTTATATTCAagaggCAGAGTTTATT

GaKCNA3210b gctagtgtgccagagttTACCTGCATTGAAGCCATTTATATGCAggggtgatgGTTTATT

+ + + +++ + ++++++++++ ++++++++ ++ + + * *******

TrKCNA3210b TGCATATTAGTCCA

TnKCNA3210b TGCATATCAGTCCATCCGAGTCA

GaKCNA3210b TGCATATTAATTTGCCCGAGTCA

******* * * ++++++++

17 of 25 positions (0.680) totally conserved.

54 of 83 positions (0.651) conserved in aligned fragments.

**Clique 133**:

| Takifugu rubripes | KCNA3b-2b | 9140 | (16) |
| --- | --- | --- | --- |
| Tetraodon nigroviridis | KCNA3b-2b | 10742 | (14) |
| Gasterosteus aculeatus | KCNA3b-2b | 12869 | (15) |

TrKCNA3210b gCTGAATCATGTCTCa

TnKCNA3210b CTGAATCATATCTC

GaKCNA3210b a-TGAATCATGTCTgc

~******** ***

11 of 13 positions (0.846) totally conserved.

11 of 16 positions (0.688) conserved in aligned fragments.

**Clique 134**:

| Tetraodon nigroviridis | KCNA6a-1a | 14641 | (26) |
| --- | --- | --- | --- |
| Takifugu rubripes | KCNA3b-2b | 9162 | (176) |
| Tetraodon nigroviridis | KCNA3b-2b | 10762 | (179) |
| Gasterosteus aculeatus | KCNA3b-2b | 12889 | (178) |
| Danio rerio | KCNA3b-3b | 44505 | (170) |

TnKCNA615a

TrKCNA3210b a-CTGACC------------------TTGTTGTCTGTCTTTGCTTTATCTGTTCTGTATG

TnKCNA3210b aCCTGACC------------------TTGTTGTCTGTCTTTGCTTTATCTGTTCTGCATG

GaKCNA3210b gCCTGACC------------------TGGTTGTCTGTCTCTGCTTTGTCTGTCCTGCATG

DrKCNA3210b atctgtattctgctttcaaacatcttTTGTTGTCTTTC----------------------

+++ + +++++++ ++~ ~~~~~~ ~~~~~ ~~~ ~~~

TnKCNA615a GGCGTCCATTGGCCATCACGTCTTAA

TrKCNA3210b CAGTGCAGTCCTA---GGT-GACATCCTGTGGCCGACAGGTCTAAAAGGTGTTGAGCCCC

TnKCNA3210b CAGCGCAGTCCTA---GGT-GACATCCTGTGGCCATCAGGTCTAAAAGGTGTTGAGCCCC

GaKCNA3210b CAGTCGGATCCTA---GGggGACATCCTGTGGCCGTCAGGTCTTTACGACGTTGAGCCCC

DrKCNA3210b ------AGTCCcacccGGA-AGCACGCTGTGGCCTTTAGAGCTCTCCGCTGTCAAGTCCC

~~~ +++ + ++ * * ***** * ** + ++ ++ +++

TnKCNA615a

TrKCNA3210b TT-CCCTTT-CCTGGCGCCTGCCTGCAGTTCTCCAGGCTGCAGGACCCACACTTACTGAA

TnKCNA3210b CCTCCCTTTcCCTGGCGACTGCCTGCAGTTCTCCAGGCTGCAGGACCCAGACTTACTGAA

GaKCNA3210b CT-CCCTCT-CCTGGCCCCTGCCTGCAGTTCTCCAAGCTGCAGGACCCCGCCTCACTGAA

DrKCNA3210b CTTCCatttggatt--CTCTTCCTGCATTTCCCCACCCTGCGGTGCCCCGCCTCTGTGAA

~++ + + + ~~ ++ ++++++ +++ +++ ++++ + +++ ++ ++++

TnKCNA615a

TrKCNA3210b AAGTGCAGGCCTCCTCCCCCT

TnKCNA3210b AAGTGCAGGCCTCCTCTCCCT

GaKCNA3210b AAGTGCAGGCCTCCACCTCCT

DrKCNA3210b AAGTGCAGGCCTCACCCTCCT

+++++++++++++ + +++

10 of 26 positions (0.385) totally conserved.

87 of 201 positions (0.433) conserved in aligned fragments.

**Clique 135**:

| Tetraodon nigroviridis | KCNA6a-1a | 15224 | (43) |
| --- | --- | --- | --- |
| Gasterosteus aculeatus | KCNA3a-2a | 6196 | (202) |
| Oryzias latipes | KCNA3a-2a | 10464 | (126) |
| Takifugu rubripes | KCNA3a-2a | 5864 | (199) |
| Tetraodon nigroviridis | KCNA3a-2a | 8714 | (200) |
| Tetraodon nigroviridis | KCNA3b-3b | 8228 | (28) |

TnKCNA615a gc----------------------------------------------------------

GaKCNA3210a tgCATTGCTGAACCCTACAGAGAACCAAACTGTGTGTGAGTCCAGTGGAAAACTTCATAT

OlKCNA3210a CTGAAtgga----------------------AGCCCAGTGGAAAAGTTCACAT

TrKCNA3210a CACATGACTGGAATTTTAAGAGATCTGATCCGcGTTTGACTCCAGTGGAAAACTTAATTT

TnKCNA3210a CACATGACTGGAATTTTAAGAGATCTGATCCGTGTGTGACTACAGTGGAAAACCTCATTT

TnKCNA3210b

~~~ ~~~ ~ ~~~~~ ~ ~ ~ ~ ~~ ~~~ ~~~~~~~~~~ ~ ~ ~

TnKCNA615a ------------------------------------------------------------

GaKCNA3210a CATCTCAGTGCagcAGGAGGTAAATATTAACATTCTAACAGACGTTTACAGCCATCACAC

OlKCNA3210a ATTCCCAGTGCCA-AacAGGTAAATATTAACATTCTAGCAAAGATTTACTGCCATCGTCC

TrKCNA3210a GGTCCCTGTGCTG-AGGAGGTAAATATTAACATTCTACCAGAGCTTCGCGGCCATCATGG

TnKCNA3210a TGTCCCTGTGCTG-AGGAGGTAAATATTAACATTCTACCAGCGCTTTGCAGCCATCGCAG

TnKCNA3210b

~~ ~ ~~~~ ~ ~~~~~~~~~~~~~~~~~~~~ ~~ ~~ ~ ~~~~~~

TnKCNA615a -----------------GCAACTCTGCCTTTTGGCTCCTCTGATTCCACTA-------AA

GaKCNA3210a TGTCATTCTGTGTACGAGCAAGTGTGCTTTATTGCTCCACTGAGCTCACCAGCTTACTAt

OlKCNA3210a TGTCGTGCTGTGTATAAACACGTTTGTTTGATTGCT

TrKCNA3210a TGTCATCTTGTGTACCAG--AGTGTGCTTTATTACTCCAGTAAACCCACCAGCTTGCTAA

TnKCNA3210a TGTCATCCTGTGTACGAG--AGTGTGCTTTATTACTCCAGTAAGCCCACCATCTTGCTAA

TnKCNA3210b TATTACTCCAACAAACCCACAATGCTGg

~~~~ ~ ~~~~~~ ~ ~~ + ++ + * **++ + +++ + ~ ~+

TnKCNA615a TCACa

GaKCNA3210a TAACTCTGCaca-TCTCCAGGCt

OlKCNA3210a

TrKCNA3210a TAACTTTGCTATCTCTCGTGGC

TnKCNA3210a CAACCTTGTTATCTCTCttagct

TnKCNA3210b

++ ++ ~++++ +++

3 of 7 positions (0.429) totally conserved.

26 of 203 positions (0.128) conserved in aligned fragments.

**Clique 137**:

| Oryzias latipes | KCNA6a-1a | 5362 | (53) |
| --- | --- | --- | --- |
| Gasterosteus aculeatus | KCNA6a-1a | 4453 | (50) |
| Xenopus tropicalis | KCNA3-2 | 41808 | (37) |

OlKCNA615a GTGATGGAATGAAAATGTTTTGTTTCTGTCTCGCGCACGATCTGAGGTTattt

GaKCNA615a GTGATGGAACAAAAATGGTGATTTAATCTCTCACCGATTTAATCCGTTTc

XtKCNA3210 AATAATTATTTGACCCCTCACTGATTTTGTAAGTTTg

+++++++++ ++*** * ** *** * * * * **

15 of 37 positions (0.405) totally conserved.

26 of 53 positions (0.491) conserved in aligned fragments.

**Clique 140**:

| Homo sapiens | KCNA6-1 | 51351 | (59) |
| --- | --- | --- | --- |
| Takifugu rubripes | KCNA6a-1a | 4155 | (131) |
| Tetraodon nigroviridis | KCNA6a-1a | 15306 | (158) |
| Oryzias latipes | KCNA6a-1a | 6116 | (108) |
| Gasterosteus aculeatus | KCNA6a-1a | 5250 | (131) |

HsKCNA615 CTGA----TAagagtt-CACCGG----------------

TrKCNA615a GGTGCGATGTAAAGTTTCCACTTGA----CACCATCTGCTGAtCTGCAGTTGGATGAATC

TnKCNA615a GCCATGTAAAGTTTCCACTCGG----CACCATCTGCTGAGCTGCAGTTGGATGAATC

OlKCNA615a GAGCCACGCAAAGTTTCCACCTGA----CACCG----CACCGGTGTTGTTGGATGATTC

GaKCNA615a GGTGCAATGTGAAGTTACCACATGAgaccCACCG----CTGCGCTGCTGTTGGATGATTT

++ ++ + + +++++ ++++ * * ~~* ~~ ~~~~~~~~~ ~

HsKCNA615 -----------------------------------------------------------C

TrKCNA615a TACCCGAGGACGGAGCGCAgcggAGCGATTTCAGGATCATCCCGGGGTAAGCATCGAAAC

TnKCNA615a CACGCGAGGACAGAGCGCATTCCAGCGATTTCAGCACCATCCCCGGGTAAGCGTCCAAAC

OlKCNA615a CACTTGAGCCAAGA------------CGTCTCAAGACCGAGCCACGGTAAGGA---AAAC

GaKCNA615a TACTCGAGGAAAGGACGCATTCAAGaCGATTCAAGATCAGCCCACGGTGAGCCCCGAAAC

~~ ~~~ ~ ~~~~ ~~ ~~~ ~ ~ ~~ ~~~ ~~ ~ ~~~*

HsKCNA615 TCCTGTC-------ATCCTCTTcgtcCTCCCATTTGTGTGTTTGACg

TrKCNA615a TGCTGACTTTGACAA

TnKCNA615a TGCTGACTTGTACAATCCGCTTga--CTCCCATTTGTGTGCTCGACc

OlKCNA615a TGCTGACTcggg

GaKCNA615a TGCTGACTTATACAA

* *** *~ ~~++++ +++ ++++++++++++++ + +++

9 of 23 positions (0.391) totally conserved.

49 of 167 positions (0.293) conserved in aligned fragments.

**Clique 151**:

| Homo sapiens | KCNA6-1 | 53133 | (67) |
| --- | --- | --- | --- |
| Takifugu rubripes | KCNA6a-1a | 3581 | (116) |
| Tetraodon nigroviridis | KCNA6a-1a | 14756 | (91) |
| Oryzias latipes | KCNA6a-1a | 5486 | (92) |
| Gasterosteus aculeatus | KCNA6a-1a | 4541 | (109) |
| Danio rerio | KCNA6a-1a | 7868 | (83) |

HsKCNA615 c-----------------------CCAGTGACTGTAGAC---------TTCCTGTGTcag

TrKCNA615a cccgttcgtTGCAGTCTGATTGCGCCAGTGCT----------------------------

TnKCNA615a cggc-------------------------GCT----------------------------

OlKCNA615a CTGGTTGCGCCAGTGCTGGCACAC---------TGTTTTTGTTG-

GaKCNA615a TGCAGTCTTATTGCACCAGAGCTGGCACACAAGTGCTCTTGTTTTTGTGG-

DrKCNA615a GCGCCAGTGTTGGCACATAAATGCTCT------------

+ ~~~~~~~~ ~~~~ ~~~~ * ~ ~ ~ ~~ ~~~~~~~ ~ ~~~

HsKCNA615 aatcCTGCAGCCTCTGTCCCTGGGCTGCTTGGGCGGA---Gt

TrKCNA615a ----CAGCCGCTCCTGCCTCTGTGGTGTTTGTGTGGA---GAACTTCTAGCGGCGTGCTT

TnKCNA615a ----CGGCCGCTGCTGCCTCTCCGGGCTCTGCGTGGG---GAACTTCTGGCGGCGTGCTg

OlKCNA615a -----------------------------TGCATGAG---GAACTTCTGACGGCGTCCTT

GaKCNA615a -----------------------------TGTGTGGA---GAACTTCTGGCGGCGTGCTT

DrKCNA615a ----------------------CGCGCACTGCGTGGAcagGCACTTGTGGCCACGCG---

~ ~~ ~~ ~~~ ~ ~~ ~ ** * * ++++ + + ++ ~~

HsKCNA615

TrKCNA615a CAGTAGAGTCGGTCTGCATTGCGTCGAAACA

TnKCNA615a gAGTAGAGTCGCTCTGCATTGCGTCGAAACA

OlKCNA615a TAGTAGAGCCGGTCTGCATTGCGTCGAAA

GaKCNA615a TAGTAGAGCCGGTCTGCATTGCGTCGAAACc

DrKCNA615a ---------CGGTCTGCATTGCGTCGAAgc

~~~~~~~ ++ ++++++++++++++++ +

5 of 13 positions (0.385) totally conserved.

33 of 151 positions (0.219) conserved in aligned fragments.

**Clique 154**:

| Gallus gallus | KCNA6-1 | 36354 | (75) |
| --- | --- | --- | --- |
| Takifugu rubripes | KCNA3b-2b | 4627 | (220) |
| Tetraodon nigroviridis | KCNA3b-2b | 6795 | (165) |
| Gasterosteus aculeatus | KCNA3b-2b | 8136 | (161) |

GgKCNA615 gcgctcAGCTGCAGACATgtAAGCACAAGATAGCTCTATTTACATTGTAGTAACTGAGAt

TrKCNA3210b gtgacaAACTGAAAATATtcAAACAAATGATTATTCTATTAATAATGCAGTAACTGAAA-

TnKCNA3210b AAA-

GaKCNA3210b AAA-

+ + + +++ + + ++ ++ ++ + +++ ++++++ + + ++ ++++++++* *

GgKCNA615 ctgATCTCTTCATCt

TrKCNA3210b ---AGTGCTTCATCATCAGGCTCTAACTTAATCTGAAATatcccatcaGCATCAAACATG

TnKCNA3210b ---ACTGCTTCATCATCAGGCTCTAACTTAATCTGAAAcacCCCGGCGGCATCAAACGCG

GaKCNA3210b ---AGCGCTTCGTCATGGTGCTGTAACTTTACCTGAGATctCTCAGGGGCACCAGAGATG

* **** ** + +++ ++++++ + ++++ + + + +++ ++ + +

GgKCNA615

TrKCNA3210b CTGTAGGTGTGATGTCACCACCCAGCCTCCTCCTATCCAGTGCTGCCTCGAGAGATGGTT

TnKCNA3210b CTGTAGGTGTGATGTCACCAGCCGGCCTCCGCCTATCCAGTGCTTCCTCTTGAGATGGTT

GaKCNA3210b CCGTGGGCGTGATGTCACCGCACAGCCTCCTC----CCACTGCTGCCCCCGGCGATGGTC

+ ++ ++ +++++++++++ + ++++++ +~~~~+++ ++++ ++ + + ++++++

GgKCNA615

TrKCNA3210b TCCGTAGCAACGAAAAGAGGCTCAGTGAAAGACTCACTCAACTA

TnKCNA3210b TCCGTAGCAACCAATAGAGGCTCAGTGGAAGACTCACCCAACTAC

GaKCNA3210b TCCGTAGCAACCgAATGAGGCTGAGGACAAGATTCACCCAGATAC

+++++++++++ + ++++++ ++ ++++ ++++ ++ +++

9 of 15 positions (0.600) totally conserved.

144 of 225 positions (0.640) conserved in aligned fragments.

**Clique 156**:

| Gallus gallus | KCNA6-1 | 37722 | (66) |
| --- | --- | --- | --- |
| Takifugu rubripes | KCNA3b-2b | 2466 | (15) |
| Tetraodon nigroviridis | KCNA3b-2b | 4407 | (26) |
| Gasterosteus aculeatus | KCNA3b-2b | 3286 | (85) |

GgKCNA615 GAGCCTGGCAGAAGTCAGGCTGgt---------------GTGGGGTTTTTGGGGTcacta

TrKCNA615a CAGCAGAAGCCTGac

OlKCNA615a TTTGTT

GaKCNA615a GAGCCCAGCAGAAGTCTGGCTGctttctctcactgttcaGTGGGTTTAGTGGGTTTTATT

+++++ +++++++ + + +++ + +++++ ++ ++++ + +

GgKCNA615 ----GATTGCTCACATCAAGCAGAg

TrKCNA615a

OlKCNA615a CTCTGTTTACGTGCAGAAAG

GaKCNA615a TTCCGTTTACTTACAGCAAGCAGAc

~~ + ++ + ++ +++++++

No position present in all sequences.

44 of 85 positions (0.518) conserved in aligned fragments.

**Clique 157**:

| Gallus gallus | KCNA6-1 | 38923 | (32) |
| --- | --- | --- | --- |
| Oryzias latipes | KCNA3a-2a | 8768 | (34) |
| Takifugu rubripes | KCNA3a-2a | 4949 | (16) |

GgKCNA615 g--GAAAGCAGTAAGTGATTTCTAGTATAACAGa

OlKCNA3210a ctAGGAAACAGCAAGTGCTTTCTCACATCACAGg

TrKCNA3210a tcAGGAAACAGCCAGc

~* ** *** ** + +++++ ++ ++++

8 of 14 positions (0.571) totally conserved.

20 of 34 positions (0.588) conserved in aligned fragments.

**Clique 159**:

| Gallus gallus | KCNA6-1 | 40818 | (37) |
| --- | --- | --- | --- |
| Gasterosteus aculeatus | KCNA6a-1a | 4244 | (58) |
| Gallus gallus | KCNA3-2 | 12203 | (58) |

GgKCNA615 GCATGAGGATATGCAAGAAACATTGTATATTTCTCAa

GaKCNA615a cCTCTGCACAGCGGTATGCAAGAATTTCTGTATTTGTCTCAGATGTGTTGCACTTCAg

GgKCNA3210 tCTGAGCACGGCGAGGCTCCATCACGTCTGTTGCTCTTCCACATTTGTTGCACTTCAt

++ *** * * * * *** * * ** ++ ++++++++++++

14 of 37 positions (0.378) totally conserved.

30 of 58 positions (0.517) conserved in aligned fragments.

**Clique 160**:

| Xenopus tropicalis | KCNA6-1 | 17820 | (27) |
| --- | --- | --- | --- |
| Gasterosteus aculeatus | KCNA3a-2a | 3119 | (172) |
| Oryzias latipes | KCNA3a-2a | 6959 | (106) |
| Takifugu rubripes | KCNA3a-2a | 2784 | (131) |
| Tetraodon nigroviridis | KCNA3a-2a | 5643 | (91) |

XtKCNA615

GaKCNA3210a AGGTGATACGTCGGGAGAAGTGGAAGAAGAGGCATCGCGTAAGGAATTATGAATGGACTG

OlKCNA3210a AGCTGCTGTGCCTGAAAACATGAAAGGAGGAGCG--GCGCGTGAAAATATGAATGAAGTG

TrKCNA3210a G--GCGTGAGGAATTATGAATGTACTG

TnKCNA3210a ATTGCGTGAGGAATTATGAATGTACTG

++ ++ + + + + + + ++ +++ ++ ++ ~ +++ + ++ ++++++++ + ++

XtKCNA615

GaKCNA3210a GGAGCACTtag----TGGCTGGCtgtcctaaGTGCACTACTGCGGGCATCCAGCCGAGGC

OlKCNA3210a GGAGCACT------------------------------ACCGCAAACGCACAGCAAAGGG

TrKCNA3210a GGAGCACGTCGCtTCAGTCTGGC--------GTGCGCTACCTCTGGCTCCCAGCAGAGGT

TnKCNA3210a GGAGCACGTCGCgTCTGTCTGGC--------GTGCACTACCTCTGGTGCCCAGAAGAGGC

+++++++ ~ ~~ ~~ ~ ~~~~~ ~~~~ ~~++ + +++ +++

XtKCNA615 TTCTGATCACAGCCCTTTATTgagtgc

GaKCNA3210a CGCTGTAGTCCAACTGCTAAACTGGTAGGTTCTGATCACTGCTCCTTGTTaaacgt

OlKCNA3210a TGCTGTTCCCTTACTGAa

TrKCNA3210a CACTGTTCCCCCACTGATCCAACCGGTGAGTATTGTGAATTCTACTTCTgtctg

TnKCNA3210a CGCCGTTCCCTCACTGAT

+ ++ + ++++ + + + + + + + + ++ + +

No position present in all sequences.

72 of 176 positions (0.409) conserved in aligned fragments.

**Clique 165**:

| Xenopus tropicalis | KCNA6-1 | 26390 | (61) |
| --- | --- | --- | --- |
| Gasterosteus aculeatus | KCNA6a-1a | 3564 | (31) |
| Homo sapiens | KCNA3-2 | 16945 | (73) |
| Takifugu rubripes | KCNA3b-2b | 2283 | (58) |
| Tetraodon nigroviridis | KCNA3b-2b | 4588 | (71) |
| Gasterosteus aculeatus | KCNA3b-2b | 4500 | (85) |

XtKCNA615 CACC---------------------------TATATATGCCTAgttatgagagtgacATT

GaKCNA615a tg----------------------------------------------------------

HsKCNA3210 ga----------------------ACAAATGCATAAATAACCA--------------ATC

TrKCNA3210b CACT---------------------------TATATGTAACTA--------------ATT

TnKCNA3210b ATTGTtc----ATGTAATGAATAGACAAATGTATAAATAACCA--------------GAT

GaKCNA3210b ATTGTcaagtaATGTGATGAATAGAt-------TATACAGCCA--------------AAT

~ ~~~~ ~~~~~~~~~ ~~~~~ ~~~ ~ ~

XtKCNA615 ATCCCTTTGGC-----------------TAATTATCATAAGAtgg

GaKCNA615a --------------------ACCACGCTTTATTACTATAAAAGCCCTTt

HsKCNA3210 CTCTGTTATttaaATATAtaACCATGCTATGTTATAATAAGAGCACTTg

TrKCNA3210b GCCTCTTTAGG-----------------TAAATATCATAAGCTGTCGAGATATTGg

TnKCNA3210b GCCTGTTTAGG-----------------TAAATATCATAACTgtgg

GaKCNA3210b GTCTGTTTTagg-ATATA---------TTTAATATCATAACTTCTCGAGATGTTGa

~ ~~ ~~~~~ ~~~~ ~~~ ** **** ++ +++

6 of 19 positions (0.316) totally conserved.

11 of 116 positions (0.095) conserved in aligned fragments.

**Clique 167**:

| Xenopus tropicalis | KCNA6-1 | 26510 | (29) |
| --- | --- | --- | --- |
| Gasterosteus aculeatus | KCNA3a-2a | 10987 | (56) |
| Oryzias latipes | KCNA3a-2a | 27355 | (90) |
| Takifugu rubripes | KCNA3a-2a | 10166 | (48) |
| Tetraodon nigroviridis | KCNA3a-2a | 12716 | (66) |

XtKCNA615 AGTGCCAGGGCAAAATCCTGCAGGT

GaKCNA3210a AGAACCAGGGTGAATTCCTGCATGT

OlKCNA3210a AAACTGGATAAAACacaca-CGTCTGCGCAACAAGAGAACCGCGGTGAATTCCTGCATGT

TrKCNA3210a GTGCTCCATCCGAATCAAGGTGAATTCCCGCACGT

TnKCNA3210a AAACTGGGCAGAACtaaattCGCCTGCGCTCCAGCTGATTCAAGGTGAATTCCTGCACGT

+++++++ + +++ + ++ +++ ++ ++ * * ** ** *** *** **

XtKCNA615 TATA

GaKCNA3210a TATT---CTCTtGAAAatatATGCTGTAGTgtga

OlKCNA3210a CATAaccATGT-GAAATa--ATGCTGTAGTccga

TrKCNA3210a CGAA---ATCT-GAAAT

TnKCNA3210a CGAttt

+ + ++++ ++++++++++ ++

14 of 29 positions (0.483) totally conserved.

53 of 94 positions (0.564) conserved in aligned fragments.

**Clique 173**:

| Xenopus tropicalis | KCNA6-1 | 29877 | (72) |
| --- | --- | --- | --- |
| Xenopus tropicalis | KCNA3-2 | 7191 | (101) |
| Takifugu rubripes | KCNA3a-2a | 6204 | (28) |

XtKCNA615 cCCGGGCTGGTGCTGTTCTCTCCGAGCAGGGGCACCAGCCcggggtaaaAGGTAAGCGAT

XtKCNA3210 CCTGGCCAGTACAGTTTTCTTCTAACCGGAGCACTGGCCatggatttcAGGTAAGCGAT

TrKCNA3210a c-----------------------------------------------------------

+~~ ~~~ ~~ ~ ~~~ ~~~ ~ ~ ~ ~~ ~~~~ ~~~ ~~ ~ ~~~~~~~~~~~

XtKCNA615 TTAAGTCTCTtg

XtKCNA3210 TTAAGTCACTggtttCCTTCTCCTTTAAGACAGAAGTACATT

TrKCNA3210a ---------------CCTTCTTCTTCACAACAAAGGTACATA

~~~~~~~ ~~ ~ ++++++ +++ + +++ + ++++++

No position present in all sequences.

21 of 102 positions (0.206) conserved in aligned fragments.

**Clique 182**:

| Gallus gallus | KCNA6-1 | 60631 | (25) |
| --- | --- | --- | --- |
| Oryzias latipes | KCNA6a-1a | 4958 | (60) |
| Takifugu rubripes | KCNA3b-2b | 8512 | (147) |
| Tetraodon nigroviridis | KCNA3b-2b | 10132 | (74) |
| Gasterosteus aculeatus | KCNA3b-2b | 12310 | (149) |

GgKCNA615 GTCTACATC

OlKCNA615a TATTTTGTTAACAATTTTTGTAAATATTcgCAGA-AGTCTGCACC

TrKCNA3210b TTTCCCTCTATGATCAATTATTTAGCAATGTTTTTGAATATTT-CAGAGAC-AGGCACC

TnKCNA3210b TTCCCTCTGTGATTAATTATTTAGCAATGTTTTTGAATATTT-CAGAGAGTAGGCACC

GaKCNA3210b tTTCCTCTCTTGTATAAATTATTTAtggtgGTTTTTGAATATTT-CAGAGAC-ATGCACC

++ + ++++ ++ + ++ +++ +++ + ++++++ ++++~+ ~ ** *

GgKCNA615 TTTT-------------CTCCAGGACAGt

OlKCNA615a TTTT-------------TTGCAGGTCAGc

TrKCNA3210b ATTTCAGGAATAAAAGATTGCAAGTCAAGGAAATCGGg-GCCAATTAATgcaaggtgttt

TnKCNA3210b ATTTCAGGAATGAAATg

GaKCNA3210b ATCTCAAGACTAAAATTTTCCAACAAAAACAAAACAGaaGTCAGTTAGTatgactgtgtg

* *~~ ~~ ~ ~~~ + ++ + +++ + + + ++ +++ + + +

GgKCNA615

OlKCNA615a

TrKCNA3210b gGTGTGTTTGGTTCGGCAGTGGATCTTAAAA

TnKCNA3210b

GaKCNA3210b tGTCTGTTTGGTGCAGCACTAAATCTTAAAA

++ ++++++++ + +++ + +++++++++

5 of 12 positions (0.417) totally conserved.

77 of 151 positions (0.510) conserved in aligned fragments.

**Clique 187**:

| Gallus gallus | KCNA6-1 | 64371 | (63) |
| --- | --- | --- | --- |
| Xenopus tropicalis | KCNA6-1 | 18626 | (48) |
| Oryzias latipes | KCNA3a-2a | 28078 | (53) |
| Takifugu rubripes | KCNA3a-2a | 10730 | (79) |
| Tetraodon nigroviridis | KCNA3a-2a | 13379 | (23) |

GgKCNA615 TAAATTTATTAAAATTCTGACATTTT--CTCTCTTCCCCTCT------CACT

XtKCNA615 taAAGAATTTAACCCTTTCAATGAAACGCAGTTGGACTCATTTCTACa

OlKCNA3210a cac--------------------------------ACCTTGTTCCCCTCCtgtgttCACT

TrKCNA3210a tgAAGAATTTAACTTATACAATATCTCGCTTTGTGACTCTCTTCCACTCC------CACT

TnKCNA3210a CACC

~~~~~~ ~~ ~ ~~ ~ ~ ~~+ +++ + + +++

GgKCNA615 GAGGTACCAGTGCAGAACa

XtKCNA615

OlKCNA3210a GCCGCGTTGCTGCAAAGCtgtaggc

TrKCNA3210a GACGTGTTGCTGCAGAACCacggga

TnKCNA3210a GCGGCGTTGCTGCAGAGCC

+ + ++++ + + ++

No position present in all sequences.

19 of 85 positions (0.224) conserved in aligned fragments.

**Clique 200**:

| Gallus gallus | KCNA6-1 | 82030 | (32) |
| --- | --- | --- | --- |
| Takifugu rubripes | KCNA3b-2b | 7360 | (83) |
| Tetraodon nigroviridis | KCNA3b-2b | 9096 | (92) |
| Gasterosteus aculeatus | KCNA3b-2b | 10906 | (98) |
| Danio rerio | KCNA3b-2b | 39746 | (88) |

GgKCNA615 AGATAGCCCCCTTGCTGGTGAAGT-

TrKCNA3210b GAATAGTTCTGGTGATG-TGTACCCCTTTAAATTGCTCACACTGCAGATATGGT-

TnKCNA3210b CT---GAACGGTCCTGGTGATG-TGTACCCCTTTAAATTGCCCACACTGCAGATATGAT-

GaKCNA3210b CC---GAAGGATGCTGGAGATGcTGTACCGCTTTAAACTGCCCACGCTGCAGATAAGGTg

DrKCNA3210b tcgtgGAAAGCTCACAGTGACG-CGT--CCCCTTTAAATGGCCACTCTGCGGATAAAGT-

+++ + + ++ + ++~~+ + ++ * * * * *** * * *

GgKCNA615 -CAGGG--------GTt

TrKCNA3210b -AAGGA--------GTGGCCTTCAGTGCGGAGGGAGAGg

TnKCNA3210b -AAGGA--------GTGGCCTTCAGTGCGGAGGGAGAGCATGCTTG

GaKCNA3210b gAAGGAgtggcgccGTGGCCTTCGGTGCGGATGGAGAGCGc

DrKCNA3210b -GAGGA--------GTGGC-----GCGCGGACGGAGCGCGTGTCTG

*** ** ++~~~~ + +++++ ++++ + + ++

15 of 32 positions (0.469) totally conserved.

45 of 106 positions (0.425) conserved in aligned fragments.

**Clique 202**:

| Xenopus tropicalis | KCNA6-1 | 38309 | (59) |
| --- | --- | --- | --- |
| Oryzias latipes | KCNA6a-1a | 4036 | (35) |
| Xenopus tropicalis | KCNA3-2 | 35626 | (49) |

XtKCNA615 AATAAAACATTAAAggatcaGTAATTCCATAGAGTAAAAATAATTTTACTTTAAAGAAT

OlKCNA615a TTTCCTAATGTGTAAATAATTTTACTCTGAAAAAC

XtKCNA3210 AACAAAAGATCAAAaaagggGTAATACCAAAGAGCGAAAATAGTTTTAg

++ ++++ ++ +++ + ++++* * * * ***** ***** + + ++ ++

14 of 25 positions (0.560) totally conserved.

36 of 59 positions (0.610) conserved in aligned fragments.

**Clique 204**:

| Gallus gallus | KCNA6-1 | 84333 | (39) |
| --- | --- | --- | --- |
| Takifugu rubripes | KCNA6a-1a | 3368 | (41) |
| Gasterosteus aculeatus | KCNA3b-2b | 5737 | (23) |

GgKCNA615 TTTTATTACCTCCTTTCATGGTGCTTAAAGCATTATGAg

TrKCNA615a TTTTATTTCCCACTTTCGTGTTACTGAACCTATCATGACAa

GaKCNA3210b TGTTATTGAGCCTATAATGACAc

+++++++ ++ +++++ ** * * * ** **** +

11 of 21 positions (0.524) totally conserved.

26 of 41 positions (0.634) conserved in aligned fragments.

**Clique 211**:

| Gallus gallus | KCNA3-2 | 14347 | (30) |
| --- | --- | --- | --- |
| Takifugu rubripes | KCNA3b-2b | 4416 | (98) |
| Tetraodon nigroviridis | KCNA3b-2b | 6550 | (67) |
| Gasterosteus aculeatus | KCNA3b-2b | 7824 | (98) |

GgKCNA3210 ACTGAAAACATAATAACGCCCTGCTGGTT

TrKCNA3210b TCTCAGGCTGCCATTCATCCATTTGCTCTTTAGAAAACATATAATAGCACTCTGGGGGCT

TnKCNA3210b ATTGAAAACATAATAGCACTCTGGGGGCT

GaKCNA3210b TCTCTGGGTGCCATTGGTCCATCTGCTCTTCACAGAACATGTAATGATACTCTGGGGGCT

++++ ++ +++++++ +++++ +++++++ * ** * **** * *** ** *

GgKCNA3210 T

TrKCNA3210b ATTTGTAAAGTTCACTGTTGTGCAATGAGACTCAGAGC

TnKCNA3210b ATTCaTAATGTTCACTGCTGTGCAATGAGACTCTGAGC

GaKCNA3210b TTTCGGAAAGTTCAGTCTTCTGCATCAGGACTCTGTGt

++ ++ +++++ + + ++++ +++++ + +

15 of 30 positions (0.500) totally conserved.

62 of 98 positions (0.633) conserved in aligned fragments.

**Clique 218**:

| Gallus gallus | KCNA6-1 | 100847 | (25) |
| --- | --- | --- | --- |
| Takifugu rubripes | KCNA6a-1a | 3759 | (68) |
| Tetraodon nigroviridis | KCNA6a-1a | 14934 | (42) |
| Oryzias latipes | KCNA6a-1a | 5647 | (128) |
| Gasterosteus aculeatus | KCNA6a-1a | 4713 | (114) |

GgKCNA615

TrKCNA615a ATCCCCCGCGCCGGAACGT--AAAATGCACGCCAAAAACTTGCTCGACACCT

TnKCNA615a GCACGGCAAAAACTTGCTTGACATCT

OlKCNA615a cCCCTCGCATCCACTGCACTTTCACGT--AAAATGCACTG--AAAACCGGCCTGACATTG

GaKCNA615a tCCCTCACCTCCCCTGCACTTggataaaaAAAATGCACAGt-AAAACTTGCGTGACATTG

+++++ + +++ + ++ + + +++++++++ ~+++++ ++ ++++

GgKCNA615 TTTTCCTTTTC

TrKCNA615a CGGAGGAAACATTCGGAC

TnKCNA615a CGAAGACAGCACTCGG

OlKCNA615a TTGAGTttt--TTTGGTCGTCGCTGCTACACCTGTCcaccatcctttgaTTTTTCTCTTC

GaKCNA615a TGGAGAAAACATTTGGACCTCAATGCTGCACTTGAC-------------TGTCCCTc---

++ ~~ + ++ + ++ ++++ +++ ++ + + + ++ ~~~

GgKCNA615 TTTTTAGAAAGgag

TrKCNA615a

TnKCNA615a

OlKCNA615a TTTTTAAAAAGaat

GaKCNA615a -------AAActgtccgt

~~~~~~ +++

No position present in all sequences.

59 of 138 positions (0.428) conserved in aligned fragments.

**Clique 222**:

| Xenopus tropicalis | KCNA6-1 | 42075 | (48) |
| --- | --- | --- | --- |
| Danio rerio | KCNA6a-1a | 5347 | (51) |
| Homo sapiens | KCNA3-2 | 21013 | (46) |

XtKCNA615 TCGTATTGATGGAGAGGGGATTTAGTTTTTTATTGAACAAACGTACTg

DrKCNA615a TTGTGGTGCTtaAAAGGGGAACTGTTTTTTTTTTAAATAAACTTATTTTCa

HsKCNA3210 ggac-----TGGGGAGAAAAACTGCCTTTATTTTAAGAAAGCTCATTTTCc

~~ * ** * * *** * ** * ** * * * ++

17 of 43 positions (0.395) totally conserved.

19 of 51 positions (0.373) conserved in aligned fragments.

**Clique 223**:

| Xenopus tropicalis | KCNA6-1 | 42849 | (44) |
| --- | --- | --- | --- |
| Xenopus tropicalis | KCNA3-2 | 36229 | (28) |
| Oryzias latipes | KCNA3a-2a | 8029 | (45) |

XtKCNA615 gCATAAACCTTTTGTTTTAATGCAGAAGCCCTGcaaaGTTTGTc

XtKCNA3210 TGAAACTAAAGCCTCTTTTCTCTTGTTa

OlKCNA3210a aCATCAGCCTCTTGTTTTGAAGCTAAAGCCTTTTTTTGTTTGTTt

+++ + +++ ++++++* * * ***** ****

12 of 27 positions (0.444) totally conserved.

25 of 45 positions (0.556) conserved in aligned fragments.

**Clique 236**:

| Gallus gallus | KCNA6-1 | 104103 | (31) |
| --- | --- | --- | --- |
| Xenopus tropicalis | KCNA6-1 | 56075 | (31) |
| Homo sapiens | KCNA3-2 | 61747 | (22) |

GgKCNA615 catcTGGAATCAAAAATAGACCCTGGCccag

XtKCNA615 cttaTGGAATAAAAAAAAAACCCTGGactat

HsKCNA3210 GAGTCAGAAATAGCCCCTGGCt

+ + ++** * * *** * ****** +

14 of 22 positions (0.636) totally conserved.

19 of 31 positions (0.613) conserved in aligned fragments.

**Clique 240**:

| Takifugu rubripes | KCNA6a-1a | 4434 | (38) |
| --- | --- | --- | --- |
| Takifugu rubripes | KCNA3b-2b | 7649 | (97) |
| Tetraodon nigroviridis | KCNA3b-2b | 9411 | (90) |
| Gasterosteus aculeatus | KCNA3b-2b | 11208 | (94) |

TrKCNA615a

TrKCNA3210b tgtgatgctctGGCTCAGGGTAATTGCATAATCTTGTTGTAGGCCACTACAGta--TAGG

TnKCNA3210b GCTCAGTGTAATTGCATAATCTCGTTGGAGGCCACCACAGtaCGTAGG

GaKCNA3210b tgggaaccga-GGCGCCCCGTTATTGCATCACCCCc--GGAGCCCGCCA-----CGTCGG

++ ++ + +++ + ++ +++++++ + + ~~+ ++ ++ + +~~~~~~~+ ++

TrKCNA615a GGGCTGCTGTCATGCGccTGTCATATCCAGCCAGTGTg

TrKCNA3210b GCGATTCTTTCATGCA--TGTCGTTCTAAGTCAGTGTCTGA

TnKCNA3210b GCGATTCTTTCATGCA--TGTCGTTTCCAGTCAGTGTCcaagtg

GaKCNA3210b CCGATTCTTTCATGCG--CGCCGTTTCAAGTCGGTGCCTGAata

* * ** ****** * * * ** * *** + +

19 of 36 positions (0.528) totally conserved.

51 of 104 positions (0.490) conserved in aligned fragments.

**Clique 241**:

| Takifugu rubripes | KCNA6a-1a | 4627 | (92) |
| --- | --- | --- | --- |
| Tetraodon nigroviridis | KCNA6a-1a | 15836 | (50) |
| Oryzias latipes | KCNA6a-1a | 6604 | (93) |
| Gasterosteus aculeatus | KCNA6a-1a | 5830 | (83) |
| Danio rerio | KCNA6a-1a | 8818 | (77) |
| Takifugu rubripes | KCNA3b-2b | 6199 | (29) |

TrKCNA615a gactAACTGAGCTAATTGCAATATGAGAG------GAATATATAAATAAATAAACTGCAG

TnKCNA615a

OlKCNA615a ggctccaTGCATTAATTGTAGTCAGAGAtcggtATAAATATAAAAATGAATAAGTgc---

GaKCNA615a AACTGCACTAATGGT----TGAGCG------AAATAAAGAAATATATGAACAGCA-

DrKCNA615a ATGAGTAGATAAATAAATAAGTAGtgt

TrKCNA3210b GCTG

+ ++ ++ ++++ + ~ ~ +++ ~~ + ++ + ++++ ++ +

TrKCNA615a AAAAATGCTGAGCCAGCAAATATCGCGGCAGTTTGATG

TnKCNA615a AAACTGCTGAGCCAGCAAATATCTGGGCAGTTTGATGCCATTATTTCctn

OlKCNA615a AGAAATGCTGAGCCAGCAAATATCTTGGCTGTTTGA

GaKCNA615a AAAATTGCTGAGCCAGCAAATATGTTGGCTGTTTGATt

DrKCNA615a gAACTTGCTGAGCAAGCAAATATCGCCGCTGTTCGATCCATTTTTCTCtg

TrKCNA3210b AAAAATGTTGAGCCTGTAAACCTCa

* ** ***** * *** * ++ +++ +++ + ++ + ++

13 of 24 positions (0.542) totally conserved.

51 of 111 positions (0.459) conserved in aligned fragments.

**Clique 242**:

| Homo sapiens | KCNA6-1 | 78480 | (37) |
| --- | --- | --- | --- |
| Takifugu rubripes | KCNA6a-1a | 4796 | (244) |
| Tetraodon nigroviridis | KCNA6a-1a | 15941 | (126) |
| Oryzias latipes | KCNA6a-1a | 6755 | (259) |
| Gasterosteus aculeatus | KCNA6a-1a | 5968 | (223) |
| Danio rerio | KCNA6a-1a | 8900 | (87) |

HsKCNA615 GAGGTCGTAGAGGAAGCAGCAGGAAGTGTTCC--------TGGAA

TrKCNA615a GAGGCAGCAGTAACAGCAGCAGCAGATACAC---------CGGTGAGCAACAATG

TnKCNA615a tgacaGAGGCAGCAGTAACAGCAGCAGCAGAAACAC---------CGGCGAGCAACAATG

OlKCNA615a GAAGCAGCAGAGGCAGCAGCAGCAAATGCTGCaGAAGCGCTGGAGAACAGCAATG

GaKCNA615a GCAGCAGTAACAACAGCAGCAGATGCTT---------TGGAGAGCAGCAATG

DrKCNA615a gtga--------CAGAGGCAGCCGAAGtg---------GATGCAGTGGAAAGGAACGATG

~~ ~ ~ ** * * * ** ~ ~~ ~~ ** + + + +++

HsKCNA615

TrKCNA615a GTCAAGTCAGCGAGAACCGCAGTGGAGCCGCACTGAGGCAGAGCAGCCAGGCAGCCCTCT

TnKCNA615a GTCAAGTCAGCGAGAACCGCAGTGGAGCCGCACTGAGGCAGAGCAGCCAGGCAGCCCTCT

OlKCNA615a GTCAAGTCAGCGAGAGCCGCAGTGGAGCCGCACTGAGGCAAAGCAGCCAACCGCCCCCCT

GaKCNA615a GTCAAGTCAGCGAGAACCGCAGTGGAGCCGCACTGAGGCAAAGCAGCCAACCGCCCCCCT

DrKCNA615a GTCGAATCAGTGACAACTGCAGTGGGGCCACGCTGAAGAAAAGa

+++ + ++++ ++ + + +++++++ +++ + ++++ + + ++ +++++ + +++ ++

HsKCNA615

TrKCNA615a CCTTTCT---GGGGACGTCTCCGGCAATTTAAGGGACGCGGGAGGCCGAAGACTGGAACA

TnKCNA615a GCCTTCC---GGGGACGT

OlKCNA615a GGCATCT---GGGGACGCCTCCAACAGTCTAAGGGCTGTGGGATGCTGAGGAGTTGAAAT

GaKCNA615a CCCGTatctgGGGGACGTCTCTGACAAATTAAGGGGTGTGGGAAGCTGAGGAGTGGAAAA

DrKCNA615a

+ +++++++ +++ ++ ++++++ + ++++ ++ ++ ++ + +++

HsKCNA615

TrKCNA615a TTTCccA------CCCGAATGCCTGCTCAGTCAGCCTgcCTGACAGCCTAGCACAACTCC

TnKCNA615a

OlKCNA615a TTTTttctctcatCCTGAATGCCTGACCAGTCAGCCTAACCAACACCCCAACACATTTCC

GaKCNA615a TTTga-A------CCTGAATGCCTGCTCAGTCAGACTAACCgggATCCCGACTCAT----

DrKCNA615a

+++ ++ +++++++++ +++++++ ++ + + ++ + ++ ~~~

HsKCNA615

TrKCNA615a TCTTCCTCCGCCGGCTccttgtcctac

TnKCNA615a

OlKCNA615a CGTTCCTCCTCCTCCTttatcacctgc

GaKCNA615a -----CTCCTCCTCtt

DrKCNA615a

~~~++++ ++ + + +++ +

9 of 22 positions (0.409) totally conserved.

133 of 267 positions (0.498) conserved in aligned fragments.

**Clique 246**:

| Homo sapiens | KCNA6-1 | 80318 | (29) |
| --- | --- | --- | --- |
| Gasterosteus aculeatus | KCNA3a-2a | 2224 | (74) |
| Oryzias latipes | KCNA3a-2a | 6001 | (83) |
| Takifugu rubripes | KCNA3a-2a | 1876 | (26) |

HsKCNA615 cacTAG----GGG--------------GAACCAGGGct----------------------

GaKCNA3210a gctccaTTCAGGG--------------GAACCAGGGTTTTTCACCTCGGTGATCCAAATT

OlKCNA3210a gctTAGTTtgt----GCTCTGTTTAAAGAATCCGGATAGTTCACTTTAGTGATATAAATT

TrKCNA3210a GTTCAGacgcGCTCCGATTAAAGAAT

~~ ~~~~ ~ ~~~~~*** + ++ ~~~~~ ~ ~~~~~ ~~~~~

HsKCNA615 ---ATTTCTCTG

GaKCNA3210a tggAATTCTTTGt---TTCCTTTCGCGCTTG

OlKCNA3210a ca----TCCCTtaaggTTCCGTTTGCGCTTG

TrKCNA3210a

~ ~++ + ++++ ++ +++++++

3 of 6 positions (0.500) totally conserved.

22 of 91 positions (0.242) conserved in aligned fragments.

**Clique 248**:

| Homo sapiens | KCNA6-1 | 81843 | (52) |
| --- | --- | --- | --- |
| Homo sapiens | KCNA3-2 | 31395 | (40) |
| Xenopus tropicalis | KCNA3-2 | 43702 | (82) |

HsKCNA615 tgactttTCTGAGTGACAGTTCCCAAAAGGGTTCCTTTTc---------ACCTTTGTtga

HsKCNA3210 CTTTGTcat

XtKCNA3210 tgtccccTCTGACTTATATTTCTTAAAAGCTTTCCTTTTtctccctattAACTT------

++ + +++++ + + + +++ +++++ ++++++++ + ***~~~

HsKCNA615 a

HsKCNA3210 cagCTTTACCTCATGGCTCAGCCACAGAGGc

XtKCNA3210 ---CTTTACCTCAGAGTTAAGCCACATAGGg

++++++++++ + + +++++++ +++

3 of 3 positions (1.000) totally conserved.

53 of 91 positions (0.582) conserved in aligned fragments.

**Clique 249**:

| Homo sapiens | KCNA6-1 | 81975 | (44) |
| --- | --- | --- | --- |
| Tetraodon nigroviridis | KCNA6b-1b | 8574 | (24) |
| Homo sapiens | KCNA3-2 | 37951 | (43) |

HsKCNA615 cagCATCAGATCTGGTCCTGACTCCAGCTTCCACTGAGTACACa

TnKCNA615b caaCATCAGCTCTGGCCCTGACTg

HsKCNA3210 ac-CATCAGCTAGGGCTGGAACTCCAACTCACTCTGAGCACACt

****** * ** *** ++ ++ + +++++ ++++

12 of 23 positions (0.522) totally conserved.

26 of 44 positions (0.591) conserved in aligned fragments.

**Clique 250**:

| Homo sapiens | KCNA6-1 | 83560 | (63) |
| --- | --- | --- | --- |
| Gallus gallus | KCNA6-1 | 80831 | (30) |
| Homo sapiens | KCNA3-2 | 40439 | (83) |

HsKCNA615 cactgccCTTCAGAAAAGGGCAGGCTGGGAATGCTGTTGTGTGGCC-------CCCTTTG

GgKCNA615 CCCTTCT

HsKCNA3210 cacaaatCTTCAGAACAGTCCCAGCTGGGAAGTCTTCCTGGTGGCCaagtgatCCCTCAC

+++ ++++++++ ++ + ++++++++ ++ ++++++ ****

HsKCNA615 GGGTGCCAGg

GgKCNA615 AGCTGCCAGCCCTGAAATGAGGt

HsKCNA3210 AGCTGCCAGCCTGGCAACAAGGg

* ****** + + ++ +++

11 of 17 positions (0.647) totally conserved.

48 of 83 positions (0.578) conserved in aligned fragments.

**Clique 255**:

| Xenopus tropicalis | KCNA3-2 | 45176 | (62) |
| --- | --- | --- | --- |
| Takifugu rubripes | KCNA3b-2b | 8238 | (122) |
| Gasterosteus aculeatus | KCNA3b-2b | 12007 | (126) |

XtKCNA3210 CATGTTAAAGACTGCCAAGCTACAGCTGATTAGTAAATC---------------------

TrKCNA3210b CAAGCTGAAGCCTGGCTTGCCGCAGCTGGTTCCTACACCTccacga--------TCCGGA

GaKCNA3210b ctgg------------TTGTCGCAGCTGGTTAtt-CACCTtgctgataccgacgTCCGGC

* * ~ ~~~ ~~~ ~ * ****** ** *~ * *~ ~~ ~~~~~

XtKCNA3210 -----------CTCAtgcttcATAA-AAAAAATCT

TrKCNA3210b AGGAGCatcgc-------CAGATAA-AATGAATATGTCTGCAGGTATGTct--------G

GaKCNA3210b AGGAGCgtgaaCTCAcagCAGATgtgAAAAAAGATGCCTGCAGGTATGTtgaagatcaaG

~~~~~~ ~ ~~~~ ** ** ** *+ ++++++++++++ +

XtKCNA3210

TrKCNA3210b AAAACACCGATTAAGTTCAcctgtca

GaKCNA3210b ATAAAGCAGCTTACCTTCA

+ ++ + + +++ ++++

21 of 42 positions (0.500) totally conserved.

47 of 146 positions (0.322) conserved in aligned fragments.

**Clique 256**:

| Xenopus tropicalis | KCNA3-2 | 45288 | (35) |
| --- | --- | --- | --- |
| Gasterosteus aculeatus | KCNA3a-2a | 8715 | (71) |
| Oryzias latipes | KCNA3a-2a | 16985 | (67) |
| Takifugu rubripes | KCNA3a-2a | 8234 | (70) |
| Tetraodon nigroviridis | KCNA3a-2a | 10826 | (70) |

XtKCNA3210 GAAACA-CTAGAAGCTGTCATTTATGGCACAGATTg

GaKCNA3210a GTGAGCGATCACCTGCTGCAGCAaCCAGCAGAGGTCATTTGGGACGGAGccCCTCCCATG

OlKCNA3210a GTGAGCCATCTCTGGCTGCAGCA-CCAGCAGAGGTCATCTAAGACAAAGATTTCTCCCTG

TrKCNA3210a GTGACAGATCACCTGCTGCAGCA-CCAGCAAAGGTCACTCGGGACAAACCTCCTCCCCTG

TnKCNA3210a GTGACAGATCACCTGCTGCAGCG-CCAGCAAAGGTCACCCAGGACACctCTCCTCCCCTG

++++ +++ + +++* * * * ** * **** * * ++ ++

XtKCNA3210

GaKCNA3210a CAGTCTGCTGt

OlKCNA3210a CTGTTTGg

TrKCNA3210a CAGTCTCCTGC

TnKCNA3210a CAGTCTCCTGC

+ ++ + ++

13 of 35 positions (0.371) totally conserved.

34 of 71 positions (0.479) conserved in aligned fragments.

**Clique 266**:

| Gallus gallus | KCNA6-1 | 108417 | (44) |
| --- | --- | --- | --- |
| Takifugu rubripes | KCNA6a-1a | 2573 | (33) |
| Oryzias latipes | KCNA6a-1a | 4469 | (66) |
| Gasterosteus aculeatus | KCNA6a-1a | 3378 | (99) |
| Danio rerio | KCNA6a-1a | 6180 | (52) |
| Gasterosteus aculeatus | KCNA3a-2a | 4627 | (172) |
| Oryzias latipes | KCNA3a-2a | 8408 | (128) |
| Takifugu rubripes | KCNA3a-2a | 4602 | (102) |
| Tetraodon nigroviridis | KCNA3a-2a | 7300 | (99) |

GgKCNA615 TTTTTTTGTCTTATAGGAATGTACTTGTTCTGGTGTTTCTG

TrKCNA615a

OlKCNA615a

GaKCNA615a AAA-CGTGTT----TTCCca

DrKCNA615a a--------------------------------------AAAA-CGTTTTGCTCTa----

GaKCNA3210a ACCACTGACCTGCAGTGACTCTTTATTCTCTTTAAGGGCACAA-TGATCTGGTCTTCCTT

OlKCNA3210a ACCACTAACCTGCAGTAGCTTTTCTTTCCCTACAGGTGTGCAGTGATTCTGGTCTTCCGG

TrKCNA3210a c---------------------TTCTTTCTTTTAGGAATGCAATGATTCTGGTCTTCCGG

TnKCNA3210a TTTTCTTTTCTTTTAGGAATGCAATGATTCTGGTCTTCCGG

~~~~~ ~~~~~~~~~ ~~ ~~ ~ ~ ~ ~ + ~ +~ ~ + ~

GgKCNA615 ------------------------------------------------------------

TrKCNA615a CTTG

OlKCNA615a AAGT----------AATGGATCCTTTTCTTG

GaKCNA615a ---------------------------TAAAGTCACAACTCA-ATTGGATCATTTTCTCG

DrKCNA615a -----------------------------GTGTCTCGCCTTA-ATTAAATCTTTCCATCG

GaKCNA3210a TTGAGCCAGTTTGGCTAAATTCGAAAGCTGAGACAGAACTCA-ACTGGACAATTTTCCTG

OlKCNA3210a ATGGGATAGATGGGTCACATTTGGGATCTGAGACAGAAATCCTTCTGGATCGGTTCTTCT

TrKCNA3210a TTGGGCC-GATGGGCCAAA-----GATTTAAGAAATGCCTCCCATTGGATCAGTTCTTCC

TnKCNA3210a TCAGGCCTGATGGGCCAAA-----GATTTAAGAAATGCCTCCCACTGGATCAGCTTTTCT

~ ~ ~ ~~ ~ ~~~ ~ ~ ~ ~ ~ ~

GgKCNA615 ----------------------------------------------------CCT

TrKCNA615a ----TACATTTTT--------ATGAATCGCCAGTGTTTCGa

OlKCNA615a ----CGGATT------ACTCCATGCATTGACAAAGTTGC-----AGAGACTTCCTGGCGG

GaKCNA615a ----CTGATATTT--------GTGCATTGCCAGTGTTTCAGCCCAGAGAACTCGTGGAGG

DrKCNA615a ----CTGAC

GaKCNA3210a atggCTGACctcctggACTCGATGCATac---------CAGCCCAGA-AATCCATCAAGA

OlKCNA3210a ----TTC----------------------CCTGT

TrKCNA3210a ----CTC----------------------Caccct

TnKCNA3210a ----Tcc

~ ~ ~~~~ ~~ ~~ ~~ ~ ~~~~~~~~ + + +

GgKCNA615

TrKCNA615a

OlKCNA615a

GaKCNA615a AAAc

DrKCNA615a

GaKCNA3210a AAAt

OlKCNA3210a

TrKCNA3210a

TnKCNA3210a

+++

No position present in all sequences.

9 of 184 positions (0.049) conserved in aligned fragments.

**Clique 269**:

| Homo sapiens | KCNA6-1 | 98797 | (52) |
| --- | --- | --- | --- |
| Gallus gallus | KCNA6-1 | 107661 | (33) |
| Oryzias latipes | KCNA3a-2a | 14782 | (51) |

HsKCNA615 TCTTCTTCctGCCTC--TACTggTGATCTTTTCTGAATGTCATCTAATACattg

GgKCNA615 TTC--TCATCAGTACTT-TGATTTTCTGTGAAAGTg

OlKCNA3210a TCTTCTTC--GCATCATCACCT-TGATTTTTTCTAAAAGTAATTTAATATgttt

+++++*** * **~ ** **** ** * * ** ** ++ +++++ ++

20 of 31 positions (0.645) totally conserved.

34 of 54 positions (0.630) conserved in aligned fragments.

**Clique 277**:

| Homo sapiens | KCNA6-1 | 115225 | (89) |
| --- | --- | --- | --- |
| Oryzias latipes | KCNA6a-1a | 3957 | (46) |
| Danio rerio | KCNA3b-2b | 25690 | (92) |

HsKCNA615 AGAAACAAACGCATATGGCTGAATCAATTAATTGC-------------------TGAAAT

OlKCNA615a ga-----------------------AATTTAATGC-------------------AGAGAA

DrKCNA3210b AGAGACAAATGCATTTGACTGAATTGCTTTATGGTgtgtagacactcatgcagtTGAAtg

~ ~~~~~ ~~~~ ~~ ~~~~~~ ** * * **

HsKCNA615 -AgatgagttgtatgttctaTCAGGCTGCCAAGAAAGGAGATTTCTTCc

OlKCNA615a -AT-----------------TAAAACTGGCACTTAAGCACACccca

DrKCNA3210b cAT-----------------TTAAACTGCCACTAAAGCACACTTCCTCt

* * * *** ** *** * * * ++

20 of 46 positions (0.435) totally conserved.

22 of 109 positions (0.202) conserved in aligned fragments.

**Clique 290**:

| Xenopus tropicalis | KCNA6-1 | 61723 | (59) |
| --- | --- | --- | --- |
| Takifugu rubripes | KCNA6a-1a | 3881 | (181) |
| Tetraodon nigroviridis | KCNA6a-1a | 15065 | (114) |
| Oryzias latipes | KCNA6a-1a | 5781 | (243) |
| Gasterosteus aculeatus | KCNA6a-1a | 4900 | (191) |
| Danio rerio | KCNA6a-1a | 8129 | (185) |

XtKCNA615 CCAAAACACActgaac----TACTGACATGAGGCAAAG

TrKCNA615a ggTCCATCCTCTCGCGTAGAGACAAAAATGCA----------------------------

TnKCNA615a CTGTCGCGTAGAGACAAAAc-GCA----------------------------

OlKCNA615a ccTCCATCCTCTCcag------CAAAAACACcccgtccgtcaTCCTGACCTGTGGCCAAA

GaKCNA615a ac----------------------------CA----------------------------

DrKCNA615a

~~~~~~~~ ~~ ~~~~~~~~ ~~~ + ~ ~ ~ ~ ~~~~~ ~~ ~~~ ~~

XtKCNA615 TGAAAGCAGGGGCAAA--------------------------------------------

TrKCNA615a -----------GCGATTTCCTGCTGATGTGATGATGCCTGGGAATGCCAAGATCTCGTTC

TnKCNA615a -----------GCGAATTCATGCTGATGCGATGATGCCTGGGACTGCCAAGATCTCGTTC

OlKCNA615a AGAAAATAGGTGCAGATTTATGCTGATGTAGTGATGTCTGGAAATGTCAAGGGCTCGGTT

GaKCNA615a -----------GCAAATTTCTGCTGATGCAGTGATGTCTGGAAATGTCAAGTGCTCGCGT

DrKCNA615a GGGAAATTTTGCTGATGCAGTGATGTCTGGAAATGgt-ATGGTTCGCTT

~~~~ ~~~ * ~ ~~~~~~~~ ~~~~~ ~~~~ ~ ~~ ~~ ~~~

XtKCNA615 ------------------------------------------------------------

TrKCNA615a ATCACCCCTT-ACCCTGTCCTACCCC----T-TGAATTCCAGCACAGGA-GTCCCCCCTG

TnKCNA615a ACCACCCCTTgACTGTGTCCTACCCC----C-TGAATTCCAGCACAG

OlKCNA615a ATTGCGCTCT-AATGTATCTCCCCCT----C-CGAACTGCAGCGCGAGG-TTCTCTCCTG

GaKCNA615a ATTGCCCCTT-AATGTGTCTCCCCCT----TcTGAATTGCTGTGCAAGAtTTTTCTCCTG

DrKCNA615a TTGCCCCCTT-AATGTATCTCCCGCCtgagC-TGAGCTGCAGCATTACA-ATTTCTCATG

~ ~ ~ ~ ~ ~~ ~ ~ ~~ ~ ~ ~ ~ ~ ~ ~~

XtKCNA615 ------------------------------------------------------------

TrKCNA615a CGGTAAATTGTG-----AGCTCTCCGCGCATCTCTGCCGtt-CGCT-CGTC---------

TnKCNA615a

OlKCNA615a CGGTACAgactgcgagcAGCTCTCAGCGC-----------CGCGCA-CCTC---------

GaKCNA615a CGGTACACTGAA-----CGCTGTTCACGCCACCGTGCCGCCGCGCGGCCAC---------

DrKCNA615a CAGTATAa----------GCCATTTACACCAAGGTGCCGCCGCACGAGCAggagcggagg

~ ~~~ ~ ~~ ~ ~ ~ ~~~~~ ~~ ~

XtKCNA615 -------CTGCTGCTT

TrKCNA615a --AGA

TnKCNA615a

OlKCNA615a --GGACTCCGCAGCTGAGCACTTCTCTCTGctacctact

GaKCNA615a --CGACGCTGCAGCTTTGCGTTTCTCTCTTttctctcct

DrKCNA615a gaGGACGTTGCGGCTTGATGTTTTTCTTTT

~~~ ++ +++ ++ +++ + + ++ ++

1 of 5 positions (0.200) totally conserved.

18 of 279 positions (0.065) conserved in aligned fragments.

**Clique 295**:

| Xenopus tropicalis | KCNA6-1 | 68769 | (30) |
| --- | --- | --- | --- |
| Tetraodon nigroviridis | KCNA3a-2a | 8540 | (68) |
| Gasterosteus aculeatus | KCNA3b-2b | 5606 | (66) |

XtKCNA615 ACTGCTTCAATTAGCAGCTAAACAGCATT

TnKCNA3210a AAGTAAtatGATGACAAAAAGTGTTTAATGTATTGCTTCTATTGGTGGTTAAATAGCATT

GaKCNA3210b AAGTAAa--GACGGCAATATTTGTATAAACTATTGTCTCATTATGTAGTTGAACTGAATT

++++++ ++ + +++ + +++ +++ +* ** ** * * * * ** * ***

XtKCNA615 a

TnKCNA3210a TGATTTGa

GaKCNA3210b TGAATTGt

++ +++

15 of 30 positions (0.500) totally conserved.

40 of 68 positions (0.588) conserved in aligned fragments.

**Clique 299**:

| Homo sapiens | KCNA6-1 | 132301 | (47) |
| --- | --- | --- | --- |
| Gallus gallus | KCNA6-1 | 112289 | (99) |
| Xenopus tropicalis | KCNA6-1 | 72316 | (100) |

HsKCNA615 tt----------------------TCTCAGGTTTCTGCTGATCTTGCAGCGCCCAGAAAT

GgKCNA615 cgCTGCCAAGCTGCATCAAACAGCTCTCAGGTTTCTGCTGATaCTGCAGCGTCAAGACAT

XtKCNA615 ccCTGCCTGGCTGCACCAAACAGCTCTCAGGTTTCTGCTGAGCCTGCAACGTCAATAAAT

~~~~~ ~~~~~~ ~~~~~~~~***************** **** ** * * * **

HsKCNA615 GGACCGAGc

GgKCNA615 GGCTCGAGTTTGCTCCTCTCTCTtaca-TATTCAGCagtc

XtKCNA615 GGCCAGAGTTTGCTTTTTACTCTtaatgTATTCAGCcttc

** *** +++++ + ++++++ ++++++++ ++

33 of 47 positions (0.702) totally conserved.

55 of 100 positions (0.550) conserved in aligned fragments.

**Clique 304**:

| Xenopus tropicalis | KCNA6-1 | 73011 | (43) |
| --- | --- | --- | --- |
| Takifugu rubripes | KCNA6a-1a | 3881 | (181) |
| Tetraodon nigroviridis | KCNA6a-1a | 15065 | (112) |
| Oryzias latipes | KCNA6a-1a | 5781 | (224) |
| Gasterosteus aculeatus | KCNA6a-1a | 5479 | (43) |
| Danio rerio | KCNA6a-1a | 8129 | (123) |

XtKCNA615 CTGTA-----------------------------------------------

TrKCNA615a ggTCCATCCTCTCGCGTAGAGACAAAAA--------------------------------

TnKCNA615a CTGTCGCGTAGAGACAAAAc--------------------------------

OlKCNA615a ccTCCATCCTCTCcag------CAAAAAcaccccgtccgtcatcctgacctgtggccaaa

GaKCNA615a CTGTC-----------------------------------------------

DrKCNA615a

++++++++ + ~~~~~~~~~~~~

XtKCNA615 --------------------------------AGTG------------------------

TrKCNA615a ----------TGCAGCGATTTCCTGCTGATGTGATGATGCCTGGGAATGCCAAGATCTCG

TnKCNA615a -----------GCAGCGAATTCATGCTGATGCGATGATGCCTGGGACTGCCAAGATCTCG

OlKCNA615a agaaaataggTGCAGat---TTATGCTGATGTAGTGATGTCTGGAAATGTCAAGGGCTCG

GaKCNA615a --------------------------------AGTC------------------------

DrKCNA615a GGGAAATTTTGCTGATGCAGTGATGTCTGGAAATGgt-ATGGTTCG

~~~~~ ~ ~ ~~~~~~~~ * ~~~ ~~~~ ~ ~~ ~~ ~~~

XtKCNA615 -----------------------------------------------------TTGCCTT

TrKCNA615a TTCATCACCCCTTaccc-TGTCCTACCCC----TTGAATTCCAGCACAGGAGTCCCCCCT

TnKCNA615a TTCACCACCCCTTGACTGTGTCCTACCCC----CTGAATTCCAGCAC

OlKCNA615a GTTATT-GCGCTCTAATGTATCTCCCCCT----CCGAACTGCAGCGCGAGGTTCTCTCCT

GaKCNA615a -----------------------------------------------------TTGCCCT

DrKCNA615a CTTTTG-CCCCCTTAATGTATCTCCCGCCtgagCTGAGCTGCAGCATTACAATTTCTCAT

~ ~ ~ ~ ~~ ~~ ~ ~ ~~ ~ ~~~~ ~ + +

XtKCNA615 GCATGATAT---------------------------CTTATTCAtgacacacag

TrKCNA615a GCGGTAAATtgtg-----AGCTCTCCGCGCatctctGCCGTTCGCTCGTCAGA

TnKCNA615a

OlKCNA615a GCGGTACAgactgcgagcAGCTCTCAGCGCcg----------CGCACCTCGGActccgca

GaKCNA615a GCAGTATAA---------------------------GCCATTCACTgtatgcat

DrKCNA615a GCAGTATAA---------------------------GCCATTTACa

++ + + ~~ ~~~~~~~ ~~~~ ~~ +

XtKCNA615

TrKCNA615a

TnKCNA615a

OlKCNA615a gctgagca

GaKCNA615a

DrKCNA615a

1 of 4 positions (0.250) totally conserved.

17 of 248 positions (0.069) conserved in aligned fragments.

**Clique 305**:

| Homo sapiens | KCNA1-5 | 135368 | (185) |
| --- | --- | --- | --- |
| Gallus gallus | KCNA1-5 | 115160 | (176) |
| Xenopus tropicalis | KCNA1-5 | 75293 | (118) |

HsKCNA615 GTGACtcatGTCACGCTTTGTAGATACTTTACTaag-TAGACTTGGAATGCTCTATTTAA

GgKCNA615 GTGACata-GTCACACTTTGTAGATGCTTTACTg--ATAGTCTTTGAATGCTTTATTTAC

XtKCNA615 c-----------------TGTAGATGCTTTAaacaaATAGTC-TTGAATGCTTAATTTtg

~~~~ ~ ~~~~~ ~~~******* ***** ~ ~*** *~* ******* ****

HsKCNA615 C--T-GTCAATGCGTTGTTGCATTGAGGATTTTgggggtgGTGAAC-CAGAAGCTTTCAA

GgKCNA615 C--TCGTAAATGCATTGTTGCATTGTGAGTTTTTGCTCA-GCGAtaa-AGAAGCTTCCAG

XtKCNA615 ccaTCGTTAATGCTTTGTTGCATTGCGAGTTTACACTCA-GTGAACaCAAAAGCTTTCAG

* *~** ***** *********** * *** * ** ~~* ****** **

HsKCNA615 GATCCATGAcaaaataaactATTTTCCTTTTATTAAAAAATGGGAAAAGAGAGAGTATtt

GgKCNA615 GATCCATGAatgataa----AATTTTGTTTACTTAAAAAAAGCAAACAAACACATTTTcc

XtKCNA615 GAT--------------------------TTGTTAAAAAAAaa

***~~~~~~ ~ ~ ~ ~~~ ~~* ******** ++ + + + + + +

HsKCNA615 tctaaaactg

GgKCNA615 acacccg

XtKCNA615

+

76 of 110 positions (0.691) totally conserved.

85 of 190 positions (0.447) conserved in aligned fragments.

**Clique 309**:

| Gasterosteus aculeatus | KCNA3a-2a | 11976 | (164) |
| --- | --- | --- | --- |
| Oryzias latipes | KCNA3a-2a | 28465 | (193) |
| Takifugu rubripes | KCNA3a-2a | 11074 | (148) |
| Tetraodon nigroviridis | KCNA3a-2a | 13628 | (173) |

GaKCNA3210a atctcAAAGATTGTATGGAGCACTCATCATATAATCCAATTTGTTAGTTGAAACTGCACC

OlKCNA3210a atatgAAAGATAGTGTGGAGCACTCAGCTTCTAATTAAATTTGTTGGTTGGAAATGCACC

TrKCNA3210a ag---------TGGATGGAGCACTCAGCATGTAACCAAATTTGTTGGTTGTAATTCCACC

TnKCNA3210a ACTGGACAGAGCACTCAGCATGTAACCAAATTTGTTGGTTGTAATTCCACC

+ ~ ~~~~~ * ********* * * *** ******** **** ** * ****

GaKCNA3210a CTGCGGGAGCTGATAGAGTGTTACTGGCTGGCTTATTGATCCTACAGTGTTCTCATTTGa

OlKCNA3210a CTGTGAGAGAAGATACAGTAATTCTtGCCACAGAAATGAGCTTGCAGGGTTCTCACATGG

TrKCNA3210a CTGCAAGAGATGATACCGTGA--CAGGCCAGCATATTGATTCTGC---------ATAAGA

TnKCNA3210a CTGCAAGAGCTGATAC-GTGG--CAGGCCAGCATATTGATCCTGCt--------ATATGA

*** *** **** ** ~ * ** * *** * * ~ ~~~~~~* *

GaKCNA3210a gggaGAACTCACAACCTAATACACACAGACACAATGGAGCCAAt

OlKCNA3210a ATGGAAACCCTTAGCCTCATACAAACAGACACTAAGGAGCCACATAGCTGTTTTGAACAT

TrKCNA3210a GAGGGAACTCATAACCTAATACAGATAGACACGGTGGAGCCAAATCAC

TnKCNA3210a AAGGGAACTCATAACCTAATACACATAGACACAGTGGAGCCAAATCACTTTATAGTTCTT

* *** * * *** ***** * ****** ******* + ++ + + + + +

GaKCNA3210a

OlKCNA3210a CCCTCCtttctag

TrKCNA3210a

TnKCNA3210a TCCTCCacccttg

+++++ ++ +

85 of 141 positions (0.603) totally conserved.

102 of 193 positions (0.528) conserved in aligned fragments.

**Clique 310**:

| Tetraodon nigroviridis | KCNA3a-2a | 13939 | (29) |
| --- | --- | --- | --- |
| Takifugu rubripes | KCNA3b-2b | 7023 | (69) |
| Tetraodon nigrovirdis | KCNA3b-2b | 8747 | (87) |
| Gasterosteus aculeatus | KCNA3b-2b | 10603 | (79) |

TnKCNA3210a c-----------------------------------------------------------

TrKCNA3210b ATCCTGGCGCAAAGATGGCAG-TTTGCTCTATTTTTGgTCACGCTTCCATTTA

TnKCNA3210b tgaattTAGCTGTGCACAAAGATGGCAG-TTTGCTCTATTTTTG-TCACACTGCCATCTA

GaKCNA3210b tgtgcaTAACCGAGCGCAGTCATGGaggtTTTGATCTATTTTAG-TCACCCTCCTATCTA

~ ~~ ~ ~~ ~~ ~~~~ ~ ~~~~ ~~~~~~~~ ~ ~~~~ ~~ ~ ~~ ~~

TnKCNA3210a -TATTTATGAGCCACGTTGCACAAAACAg

TrKCNA3210b TTATTAATGAAGCTTAT

TnKCNA3210b TTATTAATGAACCATGTTGCCCGATACAa

GaKCNA3210b TTATTGATGCCGCTTGTCGC

~**** *** * * ++ + + +++

9 of 16 positions (0.562) totally conserved.

16 of 89 positions (0.180) conserved in aligned fragments.

**Clique 311**:

| Gasterosteus aculeatus | KCNA3a-2a | 12418 | (69) |
| --- | --- | --- | --- |
| Oryzias latipes | KCNA3a-2a | 28858 | (42) |
| Tetraodon nigroviridis | KCNA3a-2a | 14154 | (70) |

GaKCNA3210a cagcaccttTATC-TCCTCGCGCtcc-GACGTGGCCCCGCCCTCTCTAAAACCTGCgggt

OlKCNA3210a CGTGGCTCCACCCAGTCTGAAAGCTGCa---

TnKCNA3210a caagaga--TATCaTCCTCCCACcactGACGTGGATCCCGCCTGTTTAAAAACctgtaaa

++ + ++++ +++++ + + + ++***** ** ** * * *** *

GaKCNA3210a cccatcTGCCt

OlKCNA3210a ------TGCCaCTTATCCTT

TnKCNA3210a ttc--------CATCTCCTT

~ ~~~~ + + +++++

15 of 28 positions (0.536) totally conserved.

39 of 80 positions (0.487) conserved in aligned fragments.

**Clique 312**:

| Gasterosteus aculeatus | KCNA2a-10a | 14176 | (141) |
| --- | --- | --- | --- |
| Oryzias latipes | KCNA2a-10a | 30580 | (181) |
| Takifugu rubripes | KCNA2a-10a | 13710 | (169) |
| Tetraodon nigroviridis | KCNA2a-10a | 15898 | (161) |

GaKCNA3210a caTATCAC-AGAGTTGCTGTCTCTCTTGTTTAGGACATTA-------TC------TGCAT

OlKCNA3210a tgtg--GG-ATAACTGCCACCTGTCTTATTATGGACATTG-------TCaatgtaTGCAT

TrKCNA3210a catgtcgtaAAAATTGCCACCTGTCTTATTTAGGATAGTtTTTGTCTTC------TGCAT

TnKCNA3210a TATTGT-AAAATCGCCACCTGTGATATTTAGGTCATTcTTCGGCTTC------TGCGT

* ~ * * ** ** * * ** ** * * ~~ ~ ~~** *** *

GaKCNA3210a AGAGTTAGTCTTACC----------TGTGTGACAcg-------CTGCTcg-----GTAGC

OlKCNA3210a GGAGTTGTTTTTTCAgtacatgcagTATGTGACAGTAGttgttCTGCTTGTAACAGTAGC

TrKCNA3210a AGAATTCATCCTACA----------TATGTGACAGTAG------TGCTTGCAACAGTAGC

TnKCNA3210a AGAATTCATCCTAAA----------TATGTAACGGTAA------TGCTTGCAATAGTAGC

** ** * * * *** ** ~ ~**** * ~~ ~*****

GaKCNA3210a CAAACAGTAGCCGAACCAGTAAtaaccaatATTAACAG---------TGTAAAGGGACCA

OlKCNA3210a CAAATAGTAGCCACACCAGTAAA-------ATTAACAA---------CATAAAGGGACCA

TrKCNA3210a CAAATAGTAGCCtat----------------TTAACAAAAAGTAACACATTAATACATTA

TnKCNA3210a CAAATCGTAGCtc---------A-------ATTAACAAAAAGTATCACAATAATGGGGTA

**** ***** ~~~~~~~ ~****** ~~~~~~ ~~ ** *

GaKCNA3210a CTAGTT

OlKCNA3210a CTAGTTTTGCATAGAATCTAAAAAACA

TrKCNA3210a CTAATATTTTAATGCATTTACAAAAGA

TnKCNA3210a ATAATATTCTAGTAAATCTACA

** * ++ + ++ ++ ++++ +

64 of 118 positions (0.542) totally conserved.

76 of 207 positions (0.367) conserved in aligned fragments.

**Clique 314**:

| Takifugu rubripes | KCNA2b-10b | 10994 | (88) |
| --- | --- | --- | --- |
| Tetraodon nigroviridis | KCNA2b-10b | 12594 | (98) |
| Gasterosteus aculeatus | KCNA2b-10b | 14733 | (105) |

TrKCNA3210b AGATGATGCGCTCACTTCATACTCATCCTGTAGTGACTCCTCACAACTTGCCCTC-AAAC

TnKCNA3210b tgt------GCTCACTTCATGCTCACCCTGTAGTGACTCCTCACAACTTGCCCTC-AAAG

GaKCNA3210b AGACCATCCGCTCACTCCTCACTCGTCCTGTcttcctTCCTCACAACTTGCCCTtaAAAC

* ~~ ~******* * *** ***** * ***************** ***

TrKCNA3210b TCCTGATAGCTGATAAACAGAGTGAAGTT

TnKCNA3210b TCCTGATAGCCGTCTAACAGAGTGAAATTTTagaaTTTTGCATGG

GaKCNA3210b TCCTGATGACTGTCAAACAGAGTGGAATTTTgcatTTCTGCATGG

******* * * ********* * **++ + ++ +++++++

59 of 82 positions (0.720) totally conserved.

71 of 105 positions (0.676) conserved in aligned fragments.

**Clique 316**:

| Takifugu rubripes | KCNA2b-10b | 11311 | (163) |
| --- | --- | --- | --- |
| Tetraodon nigroviridis | KCNA2b-10b | 13072 | (66) |
| Gasterosteus aculeatus | KCNA2b-10b | 15151 | (167) |

TrKCNA3210b AACTGTCTTATGCTTCACCCTTTAGAAACAGGGAAGATGTcaGAGCATAAAGCAAGCCCA

TnKCNA3210b g-----------------------------------------------------------

GaKCNA3210b AATTGTCTCACGCTATACCATTTTAGAAATGGGAGGATGAgtGAGCATGAAGCGAGCTCC

~ ~~~~~ ~ ~~~ ~~~ ~~~ ~~ ~~~~ ~~~~ ~~~~~~ ~~~~ ~~~ ~

TrKCNA3210b CTCCTGCGCtgatct-GCCTGCGGTGACTTGTGA---GGAGGTCCGTGGTTAAGTACACC

TnKCNA3210b ----------------------GGTGACTCGTGA---GAAGGTTGGTGGTTAAGTGCACC

GaKCNA3210b CTCCAGCGCcggtaaaGCCAGCGGCGACTCGTGtgacGGGGGTTGGTGGATAAGCACACC

~~~~ ~~~~ ~ ~ ~~~ ~~** **** *** * *** **** **** ****

TrKCNA3210b ATGTTATTTCAAGGATGtATGACAGCCATCAGCGCAAAGCTCATGGT

TnKCNA3210b A-----------GGAT------CAGCCATCAGCGCAAAGCTCATGGT

GaKCNA3210b ACGTTATTTCAAGGATGaATGACAGCCGTCAGCGCAGAGCTTATGGT

* ~~~~~~~~~~****~ ~~~~***** ******** **** *****

52 of 66 positions (0.788) totally conserved.

52 of 167 positions (0.311) conserved in aligned fragments.

**Clique 317**:

| Takifugu rubripes | KCNA2b-10b | 11599 | (25) |
| --- | --- | --- | --- |
| Tetraodon nigroviridis | KCNA2b-10b | 13268 | (72) |
| Gasterosteus aculeatus | KCNA2b-10b | 15450 | (75) |

TrKCNA3210b AGCTTTGAGGACAACTTGAATGTTT

TnKCNA3210b TTCACCCAAAATTGGTCTTACTGCAGCGCTTCGAGGACAACTTGAATGTTTCTTCTT---

GaKCNA3210b TTCACCCAAAACTGGTCTTACTGCAGAGCTTTGAGGACAACTTGAATGTTTCTTCTTgtt

+++++++++++ ++++++++++++++ **** *******************++++++

TrKCNA3210b

TnKCNA3210b TTATTTCTTTTCCAC

GaKCNA3210b TTGTTTGTTCTTCAC

++ +++ ++ + +++

23 of 25 positions (0.920) totally conserved.

65 of 75 positions (0.867) conserved in aligned fragments.

**Clique 319**:

| Takifugu rubripes | KCNA2b-10b | 12404 | (57) |
| --- | --- | --- | --- |
| Tetraodon nigroviridis | KCNA2b-10b | 14118 | (62) |
| Gasterosteus aculeatus | KCNA2b-10b | 16231 | (62) |

TrKCNA3210b AGAACGGAGAAGCCTTTGCTTAGTGTGCcGACCATTTCCATGATTTTATTCCTAATG

TnKCNA3210b AGAAAGGAGAAGCCTTTGCTTAGTGTGCAGACCATTGCCATGATTTTATTTCCAATGACA

GaKCNA3210b AGAAAGCAACAGACTTTCCACAATGCGCGGATCATTGCCATGTGTTTATTTTCAATGACA

**** * * ** **** * * ** ** ** **** ***** ****** ****+++

TrKCNA3210b

TnKCNA3210b gt

GaKCNA3210b at

+

39 of 57 positions (0.684) totally conserved.

43 of 62 positions (0.694) conserved in aligned fragments.

**Clique 320**:

| Takifugu rubripes | KCNA2b-10b | 12471 | (28) |
| --- | --- | --- | --- |
| Tetraodon nigroviridis | KCNA2b-10b | 14185 | (28) |
| Gasterosteus aculeatus | KCNA2b-10b | 16320 | (29) |

TrKCNA3210b TGTTGC-CCTGTTTCAGAGTCTGTTCAGA

TnKCNA3210b TGTTGC-CCTGTTTTAGAGTCTGTTCGGA

GaKCNA3210b TTTTCCtCCTGTTTCAGAGccgg--CGGAga

* ** * ******* **** * *~~* **

20 of 26 positions (0.769) totally conserved.

20 of 31 positions (0.645) conserved in aligned fragments.

**Clique 321**:

| Xenopus tropicalis | KCNA1-5 | 79441 | (49) |
| --- | --- | --- | --- |
| Takifugu rubripes | KCNA2b-10b | 12579 | (84) |
| Tetraodon nigroviridis | KCNA2b-10b | 14293 | (82) |
| Gasterosteus aculeatus | KCNA2b-10b | 16434 | (122) |

XtKCNA615 ATACGTAAACGTTTGCTTatttgttc-TCAAAGGAATGATTTAGATGAAa

TrKCNA3210b C------------------------------AAAACTGCCTTCTGTGAGGTAATTATTGA

TnKCNA3210b C------------------------------AAAACTGCCTTCTGTGAGGTGATTTTTGA

GaKCNA3210b ATACGAAAACGTTTGCTTcactcggaaTAAAAAAAATGCCTTCTGTGAATTAATTGTTGA

~~~~ ~~~~~~~~~~~~ ~ ~ ~~* * ** ** *** + +++ ++++

XtKCNA615

TrKCNA3210b A--GTCAGGCGTTTCCAAAAAActGTGTCATTCTA------AACCAGAAAGCCTGTTTtg

TnKCNA3210b AAAGTCGGGTGTTTCCCAAAAAacGTGTCATCCCA------AACCAGAAAGCTTGTTT

GaKCNA3210b AAGGCAGGCCGCTGTCCAAAAcgccac-CGTCCCgtacaagAGCCAGAGATTCTTTTTga

+~ + + + + + ++++ ~+ + + + +++++ + + +++

XtKCNA615

TrKCNA3210b tt

TnKCNA3210b

GaKCNA3210b gtt

+

9 of 20 positions (0.450) totally conserved.

42 of 123 positions (0.341) conserved in aligned fragments.

**Clique 324**:

| Homo sapiens | KCNA2-10 | 65022 | (80) |
| --- | --- | --- | --- |
| Oryzias latipes | KCNA2a-10a | 10906 | (61) |
| Takifugu rubripes | KCNA2a-10a | 6977 | (100) |

HsKCNA3210 AATGGGAAGAacta-----------AGAGATTCTCcctccagtcccTCTGTGTGGGTGTG

OlKCNA3210a ACAACCTTTG---------TTTATGTGTGAGTG

TrKCNA3210a AATGAGAAAAcagcgagagccctgtACACATCCTCTG---------TCTGTGTGTGTGCG

++++ +++ + + * * ** * * **** * * *

HsKCNA3210 GGTGTGTATGcatacacagtcataAACTCTt

OlKCNA3210a TGTt-----------------------TCTCTGGCAAAGGTGTCTTTcaTTATACCACAa

TrKCNA3210a TGTGTGTATGttgtt---------AACTCTCACACAAACGTCTCTTT--TTACTCCGCAc

** ~~~~~~ ~~~*** ++++ ++ +++++ +++ ++ ++

18 of 32 positions (0.562) totally conserved.

45 of 120 positions (0.375) conserved in aligned fragments.

**Clique 329**:

| Gallus gallus | KCNA2-10 | 24616 | (47) |
| --- | --- | --- | --- |
| Xenopus tropicalis | KCNA2-10 | 53154 | (43) |
| Gasterosteus aculeatus | KCNA2a-10a | 10220 | (28) |

GgKCNA3210 CTGTTTGTGCATTCGCTGCATGCCTGACTGCAGAGCCAATTGTAAGt

XtKCNA3210 TTATGTATGCACTGCATGCCTTACTGCAGAACCAATTGTAAGc

GaKCNA3210a CTGTTTGTTCATCTTCTGCATGTGTGAg

++++** * ** ******* * * ++++++ +++++++++++

14 of 24 positions (0.583) totally conserved.

35 of 47 positions (0.745) conserved in aligned fragments.

**Clique 331**:

| Homo sapiens | KCNA2-10 | 69489 | (28) |
| --- | --- | --- | --- |
| Gallus gallus | KCNA2-10 | 26513 | (19) |
| Xenopus tropicalis | KCNA2-10 | 55368 | (45) |

HsKCNA3210m, GCATCAGAATAGAGGATGGGCATGGGGg

GgKCNA3210m, CAGTGCAGGTTAGCGGAGC

XtKCNA3210m, CAGTGCAGAATAGTGAGGCATCATAAAACAGGCTGGCAATGGGGg

++++++++ +++ + **++++ ++ + +++ +++ +++++++

2 of 2 positions (1.000) totally conserved.

34 of 45 positions (0.756) conserved in aligned fragments.

**Clique 332**:

| Homo sapiens | KCNA2-10 | 69589 | (133) |
| --- | --- | --- | --- |
| Gallus gallus | KCNA2-10 | 26620 | (132) |
| Xenopus tropicalis | KCNA2-10 | 55485 | (57) |

HsKCNA3210 TCCACCTTGCTGCTGTTTTTTT---------CCAGGCAACGTCACACCTCCTGAGGACAg

GgKCNA3210 TCTACCTTCTCGCTGg-TTTTT---------CCAGGCGATGCCACACCTCCCGAGGACAC

XtKCNA3210 ACCTAGCTCCTGTTTTTTctctttcttaCCAGGCAACGTCACACCTCCTGAGGACAC

++ **** *** ~**** ****** * * ********* *******

HsKCNA3210 ccaGGACTCCAGCTTTTGCTGAGCTTTGCATCTTGCCTCCttccttcaaaAGGCTCCAGG

GgKCNA3210 cggGGACATCGGGTTTTGCTGAGCTTAGCACCTTCCCCCCgccgtaccggAGGCATCAGA

XtKCNA3210

+ ++++ + + +++++++++++++ +++ +++ ++ ++ + + + ++++ +++

HsKCNA3210 CCCATGCTCGGCTGTCTTCAAG

GgKCNA3210 CAGACGCTGCTCTGCCCTGAAG

XtKCNA3210

+ + +++ +++ + + +++

35 of 47 positions (0.745) totally conserved.

90 of 142 positions (0.634) conserved in aligned fragments.

**Clique 336**:

| Homo sapiens | KCNA2-10 | 72132 | (96) |
| --- | --- | --- | --- |
| Gallus gallus | KCNA2-10 | 29457 | (64) |
| Xenopus tropicalis | KCNA2-10 | 58955 | (94) |

HsKCNA3210 gTTCTACAGTGTGTATCTGGTTCTGCATGGAAAGCAATAGTTGTGCAAGTGACTTT----

GgKCNA3210 tTTCTACGGTGTATATTTGGTTCTGCATGGGAAGCAATAGCTGTGTAAGTGACTTT----

XtKCNA3210 TTCTA-GGTGTG---CTAGTTCTGCATGGGAAGCAATATCTGTGTAAGTGAtgtcttac

*****~ **** ~~~ * *********** ******** **** ****** *

HsKCNA3210 --------TGATCTTTTGACTtttgatttagaaCACAGAATATCTATc

GgKCNA3210 --------TAACCTTT

XtKCNA3210 tgcattccTAAC---TTGACTataact------CACAGAATATCTATt

* * ~~~*+++++ + + ++++++++++++++

43 of 56 positions (0.768) totally conserved.

64 of 108 positions (0.593) conserved in aligned fragments.

**Clique 337**:

| Gasterosteus aculeatus | KCNA2a-10a | 15351 | (60) |
| --- | --- | --- | --- |
| Takifugu rubripes | KCNA2b-10b | 12190 | (71) |
| Tetraodon nigroviridis | KCNA2b-10b | 13892 | (64) |
| Gasterosteus aculeatus | KCNA2b-10b | 16033 | (70) |

GaKCNA3210a GCTAGTGTAG---TGCTGCATGGCCTTTGAagtATTCACTGAA-GCATGAATTAG

TrKCNA3210b TTTATGCTACTCTAG---TGTTGCATGACCTTAAAAC-ATTCCTAGAA-GCATGCATTTG

TnKCNA3210b TTTATGCTACTCTAG---TGCTGCATGACCTTAAAAC-ATTCCTAGAA-GCATGCATTCA

GaKCNA3210b TTTACGCTAATCTcttgaTGTTGCATGACCACAAAAC-ATTCCTAGAgtGCATGCAg---

++++ **** * * ** ****** ** ** **** ** ***** * ~

GaKCNA3210a TATTCATAc

TrKCNA3210b CAATAAAAGAAGAAGC

TnKCNA3210b CAATAATAa

GaKCNA3210b --ATCAAATAAGAAGC

~ * * * +++++++

33 of 54 positions (0.611) totally conserved.

44 of 76 positions (0.579) conserved in aligned fragments.

**Clique 338**:

| Gasterosteus aculeatus | KCNA2a-10a | 16243 | (31) |
| --- | --- | --- | --- |
| Takifugu rubripes | KCNA2a-10a | 14964 | (30) |
| Tetraodon nigroviridis | KCNA2a-10a | 17200 | (25) |

GaKCNA3210a cTTGTGAGATAAGCTGCAGTATCAGCCgagt

TrKCNA3210a gTTCTGAGATAAGATGCGGTATCAGCCtgt

TnKCNA3210a TTCTAAGATAAGATTAGGTATCGGC

** * ******* * ***** **+

18 of 25 positions (0.720) totally conserved.

19 of 31 positions (0.613) conserved in aligned fragments.

**Clique 342**:

| Gasterosteus aculeatus | KCNA2a-10a | 16756 | (92) |
| --- | --- | --- | --- |
| Oryzias latipes | KCNA2a-10a | 32843 | (83) |
| Takifugu rubripes | KCNA2a-10a | 15325 | (71) |
| Tetraodon nigroviridis | KCNA2a-10a | 17539 | (55) |

GaKCNA3210a CGCACGTAGCCATGTAGATAATGTGTTTAAGAGTTAGAGCTGCTTTCAATGATGTCTTga

OlKCNA3210a ct---GTAGCCATG----CATTGTGTCCAAGAAGTATAGCTGCTGTCAACAATGTCTT--

TrKCNA3210a CACATATAGCCATGTAAACAGTAGGTGTACAATATATGTGTGCTTTTAATGATGTC----

TnKCNA3210a TATAGCCATGAAAACAATACATGTAAATTATATGAGCGCTTTTAATGATGTC----

+ ~~ ******** ~ ~ * * * * ** *** * ** *****~~

GaKCNA3210a AATGCCTAAAGCGTCAACTTTAAAACTATCTT

OlKCNA3210a AATGTGTACAGCTTCAGCTTTAAAACAAGCTT

TrKCNA3210a --------AAGCTTCAGCTTTAA

TnKCNA3210a --------AAG

~~~~ ~~ **+ +++ +++++++++ + +++

27 of 50 positions (0.540) totally conserved.

45 of 92 positions (0.489) conserved in aligned fragments.

**Clique 343**:

| Gasterosteus aculeatus | KCNA2a-10a | 16848 | (49) |
| --- | --- | --- | --- |
| Oryzias latipes | KCNA2a-10a | 32933 | (46) |
| Takifugu rubripes | KCNA2a-10a | 15413 | (43) |
| Tetraodon nigroviridis | KCNA2a-10a | 17623 | (47) |

GaKCNA3210a GTGCAGATTCCTGTGTAGCAGGGAGTCAACATGTA-TGCTTGCTGAATTA

OlKCNA3210a GTGTAGATTCTTTTGCAATAAGGAATAACGGTGTG-TGCTTTCTAAA

TrKCNA3210a CAGGTTCATGTGTAGCAGGGAATCACTGAGTACTGCTCGCTtt

TnKCNA3210a CAGGTACACGTGTAGCAGGGAATTAATCAGTACCGCTTGCTTAAGTA

+++ ** * * ** * * *** * * ** ~ *** ** + ++

20 of 42 positions (0.476) totally conserved.

26 of 50 positions (0.520) conserved in aligned fragments.

**Clique 344**:

| Gasterosteus aculeatus | KCNA2a-10a | 16969 | (50) |
| --- | --- | --- | --- |
| Oryzias latipes | KCNA2a-10a | 33063 | (35) |
| Tetraodon nigroviridis | KCNA2a-10a | 17738 | (53) |
| Danio rerio | KCNA2b-10b | 57964 | (51) |

GaKCNA3210a AAAATcccaaagTAAATATG-------------ATTT-C-TATGAGAACTTTAACTTATT

OlKCNA3210a AAAATgtttA--TAAATGTC-------------ATTT-C-TAAGAGTACTAT

TnKCNA3210a A--AAAATATAGGATGACATCAGAATcaaC-AATGAGAATAATAACTTATT

DrKCNA3210b A--AAAAGATCGGCTAACATCCAAATTT-CtAATAAAAATACTCTCTTTTT

+++++ * *** * ~~ ~ ~~~~~ ~** * * * * * +++ ++

GaKCNA3210a TGTGt

OlKCNA3210a

TnKCNA3210a TGTGc

DrKCNA3210b TGa

++ +

12 of 26 positions (0.462) totally conserved.

25 of 65 positions (0.385) conserved in aligned fragments.

**Clique 348**:

| Takifugu rubripes | KCNA2b-10b | 12680 | (103) |
| --- | --- | --- | --- |
| Tetraodon nigroviridis | KCNA2b-10b | 14408 | (97) |
| Gasterosteus aculeatus | KCNA2b-10b | 16583 | (113) |

TrKCNA3210b ACAAAtgAAGCCTTGCTGTCCTCTGAGAAGTCTCT-GATGCCAGGAACTCAATCTG

TnKCNA3210b TTGCCGTCCTCTGAGAAGTTTCT-GATGCCAGGAGCTCAATCTG

GaKCNA3210b aaaaACAAAa-AAGCCTGGTTGTCCTCTAAGACGTGTTgcGATGCCAGAAGCTCAAGCTG

+++++ +++++* * ******* *** ** * ******** * ***** ***

TrKCNA3210b TCTTTAAAA-CTGGCATCACAACCAAATCAGGTTCTGATTCTAGGATTG

TnKCNA3210b TCTTTAAAAACTGGCATCAAAAGCAATTTAGGTTCTAATTCTAGGCTTGAGTCC

GaKCNA3210b TCTTTAAAAGTTGGCGTCGGATGTGAACTAGGTTGGGATTCACGGATTGTGTCC

********* **** ** * * ***** **** ** *** ++++

63 of 91 positions (0.692) totally conserved.

77 of 114 positions (0.675) conserved in aligned fragments.

**Clique 350**:

| Takifugu rubripes | KCNA2b-10b | 12843 | (79) |
| --- | --- | --- | --- |
| Tetraodon nigroviridis | KCNA2b-10b | 14555 | (81) |
| Gasterosteus aculeatus | KCNA2b-10b | 16760 | (90) |

TrKCNA3210b GTGGGAAGTGATAATTAGCCGCAGTGTAGCAGGTAGCGGCTAATCTAag--GGTGGTCTG

TnKCNA3210b GTGGGAAGTGATAATTAGCCGCAGTGTAGCAGGTAGCaGCTAACCTA-----GTCGTCTG

GaKCNA3210b aTGGGGAGTGATAATCAGCAGCAGTGTAGCAGATACTGGCTAACCaagggaGGTCCTCTG

**** ********* *** ************ ** ***** * * ~ ~** ****

TrKCNA3210b ATTTTGCTCCGGAC--TCTTGTa

TnKCNA3210b ATTTTACTCCAGAC--ACTTGTCTTTtc

GaKCNA3210b ACTTCACTCCTGAggaTCTTGCATTTcttc

* ** **** ** **** +++

56 of 76 positions (0.737) totally conserved.

59 of 90 positions (0.656) conserved in aligned fragments.

**Clique 352**:

| Takifugu rubripes | KCNA2b-10b | 13474 | (86) |
| --- | --- | --- | --- |
| Tetraodon nigroviridis | KCNA2b-10b | 15125 | (83) |
| Gasterosteus aculeatus | KCNA2b-10b | 17438 | (88) |

TrKCNA3210b aGAGTGAACCACTGAATGTCAGTGTCTTTGCCTGGTTcTAAAGGTGCATTTATTTAACAG

TnKCNA3210b aaAGTGAACCACTGAATGTTGGTGTCTTTGCCTGGTTGgaaatGTGCATTTACTCAACAA

GaKCNA3210b gGAGAGAACCACTGAATGTCGGCGTCATAGCCTGGTTGTACAGCTGCATTCATGAAACAA

** ************** * *** * ******** * * ****** * ****

TrKCNA3210b TCATCTGTGTATTTGCATGTATGGGG

TnKCNA3210b CCATCTGCATATTTCCATGTATG

GaKCNA3210b CCATTTGCATATATCTGTATATGTGGgg

*** ** *** * * **** ++

56 of 83 positions (0.675) totally conserved.

58 of 88 positions (0.659) conserved in aligned fragments.

**Clique 353**:

| Takifugu rubripes | KCNA2b-10b | 13570 | (43) |
| --- | --- | --- | --- |
| Tetraodon nigroviridis | KCNA2b-10b | 15226 | (36) |
| Gasterosteus aculeatus | KCNA2b-10b | 17541 | (42) |

TrKCNA3210b ATTCTGACCAAAAAATATTTGTCTATGCAGGCAAATGCAgtat

TnKCNA3210b TCTGACTAAAAATAATTTGTCCATGCAGGCAAATGC

GaKCNA3210b AGTCCGACC-AAAAATATTTGTCTATGCATGTGAATGCAttgt

+ ** *** ~**** ******* ***** * *****+ + +

27 of 35 positions (0.771) totally conserved.

31 of 43 positions (0.721) conserved in aligned fragments.

**Clique 354**:

| Takifugu rubripes | KCNA2b-10b | 14687 | (42) |
| --- | --- | --- | --- |
| Tetraodon nigroviridis | KCNA2b-10b | 16404 | (61) |
| Gasterosteus aculeatus | KCNA2b-10b | 18784 | (59) |

TrKCNA3210b c--GAGGTATTTAGTTA--GCAGATCATTTACGGAGCTAATTTTAG

TnKCNA3210b catGAGGTATTTAGTTA--ACAGATGATTTAGGAAGCTAATTTTAGcaaaaaaAACAGCA

GaKCNA3210b cgctgtGTATTTAGTcagcAAAAATGATCTGAGGAGCTAATTTTAGccc----AAGAGCG

* ********* * * ** ** * * ************+ ++ +++

TrKCNA3210b

TnKCNA3210b TCT

GaKCNA3210b TCT

+++

30 of 42 positions (0.714) totally conserved.

39 of 63 positions (0.619) conserved in aligned fragments.

**Clique 355**:

| Takifugu rubripes | KCNA2b-10b | 14787 | (101) |
| --- | --- | --- | --- |
| Tetraodon nigroviridis | KCNA2b-10b | 16509 | (119) |
| Gasterosteus aculeatus | KCNA2b-10b | 18864 | (130) |

TrKCNA3210b CGAATCGATCAGAGGCCGCTTTGTTGTTATTTTTAGGACATTCAG--TCTTTGTAGCCCT

TnKCNA3210b a-AATCGATCAGAAGCTGCTTTGTTGTTATTCTTGGGACATTCAG--TCTTTGTAGCCAA

GaKCNA3210b CGAATTGATCACCGGGCCCTTTCTTCTTATTTTTAGGACAcctggcaTCTGTGTTGCCGC

~*** ***** * **** ** ***** ** ***** * *** *** ***

TrKCNA3210b AGGCAGTGCGGAGGACA-TTTTCAAACACCTTCTGGTATCTTCA

TnKCNA3210b CGGCA--------GACA-TTTTCAAACACCTTCTGGTATCTTCAGCTTCTtcaCTCGATT

GaKCNA3210b GGGCAGCGCGGAGGACAtTTTTCAAACGCCTCCTGGTATCTTCAGCTTCTcc-CTCTCTT

****~ ~~~~~~**** ********* *** ************++++++ + +++ ++

TrKCNA3210b

TnKCNA3210b TAAGTGCAtgg

GaKCNA3210b TAAGTGCAccg

++++++++ +

69 of 92 positions (0.750) totally conserved.

90 of 131 positions (0.687) conserved in aligned fragments.

**Clique 357**:

| Takifugu rubripes | KCNA2b-10b | 15494 | (36) |
| --- | --- | --- | --- |
| Tetraodon nigroviridis | KCNA2b-10b | 17237 | (41) |
| Gasterosteus aculeatus | KCNA2b-10b | 19591 | (41) |

TrKCNA3210b TGTTGTTTGTCGGtg-TGGTAGCATGCCCACAGGACC

TnKCNA3210b TGTTGTTTGTCGGGGATGGTGGCATGCCCACAGGACCAaag

GaKCNA3210b TGTTGTTTGTCAGGGATTGGAGGAAGCCCACAGGAGCAcgg

*********** * *~* * * * ********** *+ +

28 of 36 positions (0.778) totally conserved.

30 of 41 positions (0.732) conserved in aligned fragments.

**Clique 358**:

| Takifugu rubripes | KCNA2b-10b | 15559 | (42) |
| --- | --- | --- | --- |
| Tetraodon nigroviridis | KCNA2b-10b | 17291 | (50) |
| Gasterosteus aculeatus | KCNA2b-10b | 19641 | (49) |

TrKCNA3210b t--------CTAGTAATTAGGAGACAAGCACTCTGGGACTAGCTGCTAAT

TnKCNA3210b GCTTACCTGCCTGTAATTAGAAGACAAGCACTGTGGGACCAGCTTCTAAT

GaKCNA3210b GCGTATCAGCTGGTAATTAG-AGCCAAGCGCTGTGGGACTTGCTGTTAAc

~ ~~ ~ ~* ******** ** ***** ** ****** *** ***

30 of 41 positions (0.732) totally conserved.

30 of 50 positions (0.600) conserved in aligned fragments.

**Clique 363**:

| Takifugu rubripes | KCNA2b-10b | 20865 | (75) |
| --- | --- | --- | --- |
| Tetraodon nigroviridis | KCNA2b-10b | 20290 | (68) |
| Gasterosteus aculeatus | KCNA2b-10b | 25573 | (79) |

TrKCNA3210b AAAACAAGATGTTGCCCCAAACATCTGTC-----TTTGGAG------TATTGATCTGCAG

TnKCNA3210b TTGCCCAAAACTTCTGTC-----TTTattgatttttTATTGATCGGCAG

GaKCNA3210b AAACCTTGATGTTGTTCCAAACATCTGTgctattTATGGAG------TATTTAACCGTTT

+++ + ++++*** * **** ***** * * * **** * * *

TrKCNA3210b CTGCCGcTGCCTTCTGTCAAAACCgt

TnKCNA3210b CTGCAGg-GCCTTCTGTCAAAACtg

GaKCNA3210b CCGCCG-TGCCTTCTGTCAAAACCtc

* ** * ~***************

42 of 61 positions (0.689) totally conserved.

50 of 86 positions (0.581) conserved in aligned fragments.

**Clique 364**:

| Takifugu rubripes | KCNA2b-10b | 20951 | (53) |
| --- | --- | --- | --- |
| Tetraodon nigroviridis | KCNA2b-10b | 20370 | (88) |
| Gasterosteus aculeatus | KCNA2b-10b | 25670 | (89) |

TrKCNA3210b CAATTTCTTCCTAAGACACTACCA--TTGCATCGTTAATCCAGG-AGGATGCGTGA

TnKCNA3210b CAATTGCTTCCTAAGACGCAACCA--TTGCATTCCTAATCCAGGaAGGATGCGTGActGA

GaKCNA3210b CAACTGCTCCTTAAGACTTTGCCtgtTTGGATACTTAATCAGAG-AGGATGCGTcttaGA

*** * ** * ****** ** *** ** ***** * ********* ++

TrKCNA3210b

TnKCNA3210b GTACAAACAAGCTTCTTATGCAGCTGAATG

GaKCNA3210b GTCAAGAGGAGCTTCTTTCACCGCTGGATG

++ + + ++++++++ + ++++ +++

35 of 53 positions (0.660) totally conserved.

57 of 90 positions (0.633) conserved in aligned fragments.

**Clique 374**:

| Gasterosteus aculeatus | KCNA2a-10a | 22407 | (32) |
| --- | --- | --- | --- |
| Oryzias latipes | KCNA2a-10a | 38118 | (31) |
| Takifugu rubripes | KCNA2a-10a | 20895 | (29) |
| Tetraodon nigroviridis | KCNA2a-10a | 22589 | (32) |

GaKCNA3210a CACTGAAGGTAGGCCGCTTCCCTCTC-CTCCGa

OlKCNA3210a c-CTGAAGGTAAGCTGGTTTGCTCTttCTACc

TrKCNA3210a CAGTGCAGGTAGGCCGTTTTCCACTC-CTC

TnKCNA3210a CAGTGCAGGTAGGCGGCTTTGCACTC-CTCCGc

*~ ** ***** ** * ** * ** ** +

18 of 28 positions (0.643) totally conserved.

19 of 33 positions (0.576) conserved in aligned fragments.

**Clique 375**:

| Homo sapiens | KCNA2-10 | 72469 | (129) |
| --- | --- | --- | --- |
| Gasterosteus aculeatus | KCNA2a-10a | 22827 | (51) |
| Danio rerio | KCNA2b-10b | 46892 | (97) |

HsKCNA3210 tggctTAGCTTTAAATAGCTGGTGTTTGTTCAAaaggtcatactcattttTGCAttgaaA

GaKCNA3210a tatcaTAGCTTTAAATAGATAGTATTTATTAAA-----------------TGCA------

DrKCNA3210b a-----------AAAGTGCTAGTGTATTTTgtgt-------------------------A

~ ~~~~~~~*** * * ** * * ** ~~~~ ~

HsKCNA3210 AGAACACACAGTCCTGTGTGTTGGAATTACTTTCTGTGtcaCAGGCTGGAGtttgtg---

GaKCNA3210a -----------TCATGCGTGTcttg

DrKCNA3210b AGAGCATACTGTTCAGTGTAATTGTTCTATTGTTTCTGctgCAAGCTGCAGagatagtcc

~~~ ~~ ~~ ~* * ** ++ + + + ++ ++ ++++ ++ +

HsKCNA3210 -AATTGCAGTTGc

GaKCNA3210a

DrKCNA3210b aAAATGTAGTTGa

++ ++ +++++

15 of 36 positions (0.417) totally conserved.

40 of 133 positions (0.301) conserved in aligned fragments.

**Clique 377**:

| Homo sapiens | KCNA2-10 | 72872 | (99) |
| --- | --- | --- | --- |
| Gallus gallus | KCNA2-10 | 30107 | (101) |
| Xenopus tropicalis | KCNA2-10 | 60082 | (38) |

HsKCNA3210 gaTGTGGCCcagcTACACTgac-----ACTGCAGTGAAATTTA-CCAGTGATGCAATAGA

GgKCNA3210 gcTGTGGCC----TACAATatatttatACTGCAGTGAAATTTAaCCAGTAATACAGTACA

XtKCNA3210

+ +++++++ ++++ + ++++++++++++++++ +++++ ++ ++ ++ +

HsKCNA3210 GCTGCATGGGTGTTTGTTGCATGAATCTCAATATT--TAAAACACAg

GgKCNA3210 GCTGCATGGGTATCTGTTGCATGAATCTGAATATT--TAATACACAc

XtKCNA3210 GCATGAATATATATTGCATGCACACAAATATAgtTAAA

+++***** * * * ******* * ***** *** +++++

24 of 36 positions (0.667) totally conserved.

73 of 107 positions (0.682) conserved in aligned fragments.

**Clique 378**:

| Homo sapiens | KCNA2-10 | 72979 | (49) |
| --- | --- | --- | --- |
| Gallus gallus | KCNA2-10 | 30214 | (54) |
| Xenopus tropicalis | KCNA2-10 | 60137 | (73) |

HsKCNA3210 c--TATTTTCCTAG-------------------------TAGCCTAcACAGTGTTAATAT

GgKCNA3210 atATATTTTCTTAG-------------------------TAGACTAtACAGTATTCATGT

XtKCNA3210 acATATTTTTTTAGccgaaaaaattatcagctatctgtgTAGCt------GTGTCCATGT

~****** *** *** ~~ ~~~** * ** *

HsKCNA3210 TTAGAGTATTAATAgg

GgKCNA3210 TTATAGTGTTTATAgactc

XtKCNA3210 TTATAGTATTTATActctt

*** *** ** *** ++

29 of 43 positions (0.674) totally conserved.

31 of 79 positions (0.392) conserved in aligned fragments.

**Clique 386**:

| Homo sapiens | KCNA2-10 | 81740 | (48) |
| --- | --- | --- | --- |
| Takifugu rubripes | KCNA2b-10b | 11711 | (189) |
| Tetraodon nigroviridis | KCNA2b-10b | 13352 | (243) |
| Gasterosteus aculeatus | KCNA2b-10b | 15537 | (250) |

HsKCNA3210 TAAA------

TrKCNA3210b TGACA

TnKCNA3210b ACACGCGCACACGtaatac--TATTCAAAAAGataggtaggAACTGTGACTAAACTGATA

GaKCNA3210b ACACGCACATACGcacacccaTATTCAAATAGtaggtt---AACTGTGACTTTACTGACA

++++++ ++ +++ + + ++++++++ ++ + + ++++++++++ +~~~~ ~

HsKCNA3210 ------------------------------------------------------------

TrKCNA3210b GgttaG-GGTAGGTAGGTGCTGTTATGCCGAAGCATAT------TAGATTATTGCATGAC

TnKCNA3210b GCC--G-GGTAGGTAGGTGCTTTTATGACTAAGCATAT------TAGAATATTGCATGAC

GaKCNA3210b GCCgataGGTAGATAAGTGCTGTAAGTAGTAAACATATagccaaTAGATTATTGCATGAC

~ ~ ~~~~~ ~~ ~~~~~ ~ ~ ~~ ~~~~~ ~~~~ ~~~~~~~~~~~

HsKCNA3210 ATTTTTGACAGACACTGATGGTTTTCACTTGTTTCTTGCCTGCt

TrKCNA3210b ATTTTTTAAACTAAATGACAATTTCCACTTGTTTCTCAACTGCCACTTGCCTCATCACgC

TnKCNA3210b ATTTTTTAAACGAAATGACAATTTCCACTTGTTTCTCAACTGCCACTTGCCTCATCACAC

GaKCNA3210b ATTTTTCTCAG--AATGAGAAGTTCCACTTGTTTCTCGACTGCCACTTGCTTCatggCAC

****** * * *** ** *********** **** ++++++ ++++ + +

HsKCNA3210

TrKCNA3210b CACTGTATATTTGTCTATACACTGCCTTCAAAACAAgACTACACTTTAATAAAGAAGC-C

TnKCNA3210b CACTGTATATTTGTCTATACACTGCCTTCAAAACAAAACCACACTTTAATAAAGATGC-C

GaKCNA3210b CACTGTATATTTGTGTGCACACTGCCTTCTATACATATCCACACTTTTAAAAAGgcccaC

++++++++++++++ + +++++++++++ + +++ + +++++++ + ++++ + +

HsKCNA3210

TrKCNA3210b TGCAGGTAGCAT

TnKCNA3210b TGCAGGTAGCATTCA

GaKCNA3210b TGCAGGTAGCTGTCA

++++++++++ +++

28 of 42 positions (0.667) totally conserved.

134 of 255 positions (0.525) conserved in aligned fragments.

**Clique 387**:

| Xenopus tropicalis | KCNA1-5 | 86404 | (32) |
| --- | --- | --- | --- |
| Homo sapiens | KCNA2-10 | 82867 | (60) |
| Gallus gallus | KCNA2-10 | 32566 | (54) |
| Xenopus tropicalis | KCNA2-10 | 61344 | (71) |

XtKCNA615 TGTGTGAAA------------

HsKCNA3210 CCTCCAAGAAGCTGGGATGCTGGGAAGatgggagctggg

GgKCNA3210 TGAGGGGAGGCTCTTATGTTGGCTGAGTGctttaGGAATGGTGGGAGA------------

XtKCNA3210 TGATGCAACTCTCCTATGTGGCCTCAATGAAGCTGGAATTGTGGGAAA------------

+++ + + +++ +++++ + ++ + ++ ++ ** **

XtKCNA615 ATGCTTTCTGGATAGTAGGCGGG

HsKCNA3210 AAGAATTTTGTCTAAACAGTc

GgKCNA3210 AAACTg

XtKCNA3210 AAGCTTTTTGGTTAAAAAGTGGT

* + ++ ++ + +

5 of 15 positions (0.333) totally conserved.

33 of 83 positions (0.398) conserved in aligned fragments.

**Clique 391**:

| Xenopus tropicalis | KCNA1-5 | 86683 | (80) |
| --- | --- | --- | --- |
| Xenopus tropicalis | KCNA2-10 | 81935 | (63) |
| Oryzias latipes | KCNA2a-10a | 37729 | (41) |

XtKCNA615 TTACTTTCTGCACTGGAATAATTAATTCC--AGCATGGATAATACACTTGTTATTAA---

XtKCNA3210 ATTCCatAGTATTGCTCAATTACTCATTAATAAaca

OlKCNA3210a TTTTTTTCTGCACAGGGATGATTCATTCA--AGAGGGGAGAAa

++ +++++++++ ++ ++ +++ **** ** * * +++ +++ +++

XtKCNA615 --TAGTGATGAGCGAATCTGTCCtgtt

XtKCNA3210 atTAGTTATGAGCGAATCTGTCCcatt

OlKCNA3210a

++++ ++++++++++++++++ ++

8 of 17 positions (0.471) totally conserved.

57 of 87 positions (0.655) conserved in aligned fragments.

**Clique 399**:

| Oryzias latipes | KCNA2a-10a | 43018 | (48) |
| --- | --- | --- | --- |
| Takifugu rubripes | KCNA2a-10a | 21696 | (48) |
| Tetraodon nigroviridis | KCNA2a-10a | 23419 | (44) |

OlKCNA3210a CAGGAAGTTTTTAATTTATCAGgAAAAGATTTTGGCTGACCTTTGCTg

TrKCNA3210a CAGGAAGTGTTTGATTTGCCAttAAGCGATTTGGGCTGCCCTGTGCTC

TnKCNA3210a CAGGAAGTGTTTGATTTACCAGt----GATTTTGACTGTACTTGGCTC

******** *** **** ** ~~ ***** * *** ** ***

31 of 44 positions (0.705) totally conserved.

31 of 48 positions (0.646) conserved in aligned fragments.

**Clique 400**:

| Gallus gallus | KCNA2-10 | 37315 | (30) |
| --- | --- | --- | --- |
| Oryzias latipes | KCNA2a-10a | 43768 | (32) |
| Takifugu rubripes | KCNA2b-10b | 15352 | (30) |
| Tetraodon nigroviridis | KCNA2b-10b | 17081 | (79) |
| Gasterosteus aculeatus | KCNA2b-10b | 19437 | (83) |

GgKCNA3210 GCTGCCATCGGGGCAAAGCAGGAT-

OlKCNA3210a TCCAGT-TATGATGTGCAACACTGGACAAAAAg

TrKCNA3210b TCCAGT-GATGTAGTGCAACACTGCAGAAAc

TnKCNA3210b cttttTCTGATCCAGT-GATGCAGTGCAACACTCTGCAGAAACCAGGGCAAACGGGGAaa

GaKCNA3210b ctcgcTCTGATCCgcccGATGCAGTGCAGCACTCTGCAGACGCCTGGGCAAAACAAGGT-

++ ++++++++ +++ +++++ ++++ + +++++++ +

GgKCNA3210 GAGAGc

OlKCNA3210a

TrKCNA3210b

TnKCNA3210b GAGAGGCa----GAACGGGGCTGc

GaKCNA3210b GAGAGGCgggtgGGATGGAGCGGt

+++++ + + + ++ ++ +

0 of 6 positions (0.000) totally conserved.

44 of 84 positions (0.524) conserved in aligned fragments.

**Clique 407**:

| Xenopus tropicalis | KCNA1-5 |  | (40) |
| --- | --- | --- | --- |
| Gasterosteus aculeatus | KCNA2a-10a |  | (73) |
| Oryzias latipes | KCNA2a-10a |  | (67) |
| Takifugu rubripes | KCNA2a-10a |  | (15) |
| Tetraodon nigroviridis | KCNA2a-10a |  | (74) |

XtKCNA615

GaKCNA3210a GGAAGAGAAGAAA------------GGTTGGGAAGGAAAAATAcgataatgaaagtaaac

OlKCNA3210a GGAAATGAAGAAAaatgcatggaaaTGTCCTG-ACGAAAGTTAGA---------------

TrKCNA3210a

TnKCNA3210a a---------AAA------------TGCTCTGAAAGAAAAATAGA---------------

~~~ ~~~~+++ + +~+ ++++ ++

XtKCNA615 TCTGCTTTATATTGCATCTATTTTAAAGCACCCAC

GaKCNA3210a actggcCACAC---------CCTCTTTTGCTGTT

OlKCNA3210a ------CAAAgtgctcgacaCCTCCTTTt

TrKCNA3210a CACAT---------CCTCCTTTGC

TnKCNA3210a ------CACAT---------CCTCCTTTGCTTTTTACTGCAACTACTGATGAGCACTTGC

++ + ++++ * * ++ + ++ ++++ +++ + +++++ +

XtKCNA615 ACTTg

GaKCNA3210a

OlKCNA3210a

TrKCNA3210a

TnKCNA3210a ATTTa

+ ++

2 of 4 positions (0.500) totally conserved.

43 of 125 positions (0.344) conserved in aligned fragments.

**Clique 410**:

| Xenopus tropicalis | KCNA2-10 | 84695 | (45) |
| --- | --- | --- | --- |
| Takifugu rubripes | KCNA2b-10b | 15146 | (40) |
| Danio rerio | KCNA2b-10b | 55766 | (25) |

XtKCNA3210 TTTTGAAATCACAAAAAACTGACTTTCTCTAAAGCCATGAATGTc

TrKCNA3210b TTTAGGTGTAAAAAAAAATTGTATCTCTCTAAAGCAATGc

DrKCNA3210b GACTCTCTCTGAGGTCAGGAATGTa

+++ + + + ++++++ +* * ***** * * * * ++++

11 of 20 positions (0.550) totally conserved.

28 of 45 positions (0.622) conserved in aligned fragments.

**Clique 421**:

| Xenopus tropicalis | KCNA2-10 | 92750 | (42) |
| --- | --- | --- | --- |
| Takifugu rubripes | KCNA2b-10b | 10866 | (59) |
| Tetraodon nigroviridis | KCNA2b-10b | 12486 | (79) |
| Gasterosteus aculeatus | KCNA2b-10b | 14589 | (59) |

XtKCNA3210 ACATGTATA

TrKCNA3210b aAAATGTTGAACtaagttACATGACAGGACAGATGTACAGCCGCCAATCTTACTTGTGC

TnKCNA3210b a--------------GCAACACGACAGGGCAGATGTACAGCCTCCCATCTTACATGTGCT

GaKCNA3210b gAAATGCGGAGCcggGCAACTGGACGGGACAGGCGCACAGCCTCCCATCTCCCGTGTGC

~~~~~ ~~ ~ + ++ +++ ++ +++ + ++++++ ++ ++++ * ***

XtKCNA3210 GGTAAATTAAAGCTGATGGCATATGGGATATat

TrKCNA3210b

TnKCNA3210b GGAAAATTCAAGGCAATAGCATTAAGGATGTag

GaKCNA3210b

++ +++++ +++ ++ ++++ ++++ ++

4 of 8 positions (0.500) totally conserved.

50 of 93 positions (0.538) conserved in aligned fragments.

**Clique 425**:

| Xenopus tropicalis | KCNA2-10 | 109908 | (35) |
| --- | --- | --- | --- |
| Takifugu rubripes | KCNA2b-10b | 11493 | (85) |
| Tetraodon nigroviridis | KCNA2b-10b | 13168 | (80) |
| Gasterosteus aculeatus | KCNA2b-10b | 15318 | (106) |
| Danio rerio | KCNA2b-10b | 50543 | (55) |

XtKCNA3210 aggc---------------------------ACAGCAACATCTGAAGGATGGCTGTTAAT

TrKCNA3210b AGCAGGCATCAGTATCTGATGAATGGCTGAGAAT

TnKCNA3210b AGGCATCAGTATCTGATGAATGGCTGTGAAT

GaKCNA3210b tcgacacCTCTTTTTTTTTTTTTCCCAGCAGACATCGGTATCTGATGAATGACTGAGGAT

DrKCNA3210b ttgaagtCTTCTGTTTTTTTTTCCTCATCGGACAGGCACATCTG-TGAAACACTGg

+ ~~ ~ ~~~~~~~~~ ~ ~~ ~ ~ ** *****~ * * *** ++

XtKCNA3210 Ga

TrKCNA3210b GGCCTTGACTTTTCACTGCTTTGGTCTTACATTACTGTATGTATTTCAATG

TnKCNA3210b GGCCTTGACTTTTCACTGCTTTGGTCTTACATTACTGTTTGTATTGCAg

GaKCNA3210b aGCCTTGACTTTTCACTGCTTTGGTCTTAC-----TGTCTGTATTGCAATG

DrKCNA3210b

++++++++++++++++++++++++++++~~~~~+++ ++++++ ++ ++

12 of 24 positions (0.500) totally conserved.

56 of 111 positions (0.505) conserved in aligned fragments.

**Clique 429**:

| Xenopus tropicalis | KCNA2-10 | 117042 | (40) |
| --- | --- | --- | --- |
| Tetraodon nigroviridis | KCNA2b-10b | 14658 | (44) |
| Gasterosteus aculeatus | KCNA2b-10b | 16893 | (24) |

XtKCNA3210 tcagTAAGTTCAGCAGAAGAAATGTGCGTA-GGAGGGAACa

TnKCNA3210b ttaaTAAGTCCAGAAAAAAAAATGGGCCTA-AGCAAGAACTtttt

GaKCNA3210b AGA----------GGCtcgtAGCGAGAACTgtct

+ + +++++ +** ~ ~~ ~~~~~ ** * **** + +

9 of 19 positions (0.474) totally conserved.

19 of 45 positions (0.422) conserved in aligned fragments.

**Clique 441**:

| Xenopus tropicalis | KCNA1-5 | 112115 | (32) |
| --- | --- | --- | --- |
| Gallus gallus | KCNA2-10 | 50334 | (36) |
| Gasterosteus aculeatus | KCNA2a-10a | 24301 | (98) |
| Oryzias latipes | KCNA2a-10a | 43095 | (121) |
| Takifugu rubripes | KCNA2a-10a | 21806 | (93) |
| Tetraodon nigroviridis | KCNA2a-10a | 23500 | (95) |

XtKCNA615 GAAG----------------------------------------

GgKCNA3210 CTGCTGGATGCTGCTGCTCAGAGCTG

GaKCNA3210a tTGTCAAAAGGCCAAGGGAGGATTTTaGTCCCAGCTGCTGTGCACTGGGACTTGGCCCGG

OlKCNA3210a cTGTCAAAAAGCCACGGGAGGATTTTGGTCCCAGCTGCTGTGCACTGAGACTTGGCCTGA

TrKCNA3210a gg-----------------GAGATGTTTTCCCAGCTGCTGTGTtaa-GTTCTTGGCCTGG

TnKCNA3210a AGGAAGAGTTGTTTTCCCAGCTGCTGGGTACTGGTGCTTGGCTTAG

~~~~~~~ ~~~~ ~~ ~+ ~ ~ ~~~~~~~~~~~~ ~ ~~ ~

XtKCNA615 --------------------------------TCA-ACACCTCATTGATGCTTTGCAGGG

GgKCNA3210 AGCGTGTGGc

GaKCNA3210a AGCCTCCAGACGCACCCATGTCAGCATCAGCATCA-GCA

OlKCNA3210a CGCCTGCAGACACACTCATGTCAACATCAGCAGCTTACATCTTATGAATGCCTTGCAGGG

TrKCNA3210a AGCCTGCGGACACAATCATGTTCCCCTCAGCAGCTTAAT--TTAAGAATACCT

TnKCNA3210a AGCCTGTGGACACAAGCATGTTTCTCTCAGCAGCTTAAT--Tcc-GAATACC

~~ ~ ~ ~ ~~ ~~~~~ ~~~~~~ + ~ ~+ ++ + ++++++++

XtKCNA615 a

GgKCNA3210

GaKCNA3210a

OlKCNA3210a c

TrKCNA3210a

TnKCNA3210a

No position present in all sequences.

14 of 121 positions (0.116) conserved in aligned fragments.

**Clique 444**:

| Tetraodon nigroviridis | KCNA1b-5b | 15777 | (31) |
| --- | --- | --- | --- |
| Gallus gallus | KCNA2-10 | 32677 | (29) |
| Takifugu rubripes | KCNA2b-10b | 20347 | (22) |

TnKCNA615b CTGTTGTTGTTCCATAAATCTGCATCCTGAt

GgKCNA3210 GTTGAAGTCCTATAAATCTGGATCCTGAg

TrKCNA3210b CTGCTGTTGTGCCATAAATCTc

++* ** ** * ******** +++++++

14 of 20 positions (0.700) totally conserved.

23 of 31 positions (0.742) conserved in aligned fragments.

**Clique 456**:

| Xenopus tropicalis | KCNA1-5 | 123715 | (33) |
| --- | --- | --- | --- |
| Gallus gallus | KCNA2-10 | 32381 | (26) |
| Takifugu rubripes | KCNA2b-10b | 13058 | (42) |
| Gasterosteus aculeatus | KCNA2b-10b | 17018 | (65) |

XtKCNA615 ACTCGTTCAACTAATCTGCATGAGGCTACcaga

GgKCNA3210 TCTCCGCTCTGTGTCTGCTCc

TrKCNA3210b CCTTCCAACTTCCTGAAATGAGGCTACATGCG----------------TGTCTCCTta

GaKCNA3210b ACCCGTCCAACTGCCCGGCGTGAGGCTTCTGGCGtcgtcTCTCTGTTCTGTGTCTGCTCt

++ + + +++++ +++++++ + + + ~~~~ ~ ~~~~+++++ ++

XtKCNA615

GgKCNA3210 tatgg

TrKCNA3210b

GaKCNA3210b cgtgt

++

No position present in all sequences.

28 of 65 positions (0.431) conserved in aligned fragments.

**Clique 459**:

| Xenopus tropicalis | KCNA1-5 | 126867 | (25) |
| --- | --- | --- | --- |
| Takifugu rubripes | KCNA2b-10b | 17949 | (33) |
| Gasterosteus aculeatus | KCNA2b-10b | 21376 | (15) |

XtKCNA615 TTATTTAGATATTTGGACTTGTTCc

TrKCNA3210b TTATTTAGATATTGGGACTTGATCACTTagcat

GaKCNA3210b ACTCCATCACTTgat

+++++++++++++ ++*** ** +++

5 of 9 positions (0.556) totally conserved.

23 of 33 positions (0.697) conserved in aligned fragments.

**Clique 467**:

| Xenopus tropicalis | KCNA1-5 | 130023 | (54) |
| --- | --- | --- | --- |
| Tetraodon nigroviridis | KCNA2b-10b | 23066 | (109) |
| Gasterosteus aculeatus | KCNA2b-10b | 28729 | (113) |

XtKCNA615 gt------------------GAGAGTGGGA------GGGGTTGGGACACACCTTTTAGCA

TnKCNA3210b ACTCCCGCCTGCACCAGGATCAGAGTGGGATGa---GGGAATGGATTCCAACTTTTGGCA

GaKCNA3210b ACTCCGGGCTGCGGCGGGAGGAGAGCGAGATGacggGGGGACGACTTGTGACTTCAGGCA

~~~ ~ ~~~~ ~ ~~~ **** * **~~~ *** * *** ***

XtKCNA615 ATT-AGGTATTTATTAGGA

TnKCNA3210b ACT-GGTTGTTTATCAGGTAACATAATACCCATCCTtcaggtCCAAATTGGCc

GaKCNA3210b ACacGGTTCTCTATCAGGAAACGCAATAGTCAACCTgtggtgCCAAGTTGGCt

* * * * *** *** +++ ++++ ++ +++ + ++++ +++++

27 of 54 positions (0.500) totally conserved.

49 of 113 positions (0.434) conserved in aligned fragments.

**Clique 473**:

| Xenopus tropicalis | KCNA1-5 | 131341 | (59) |
| --- | --- | --- | --- |
| Gallus gallus | KCNA2-10 | 55157 | (67) |
| Xenopus tropicalis | KCNA2-10 | 111596 | (21) |

XtKCNA615 CTTTTTTAGACTGGTAAATAC--AACACtagtg------CAGGGGTGTGCAAGGGAACGT

GgKCNA3210 CTCCTTTCTGCTGGTAGATACccAATGCAggggggtctcCAGGGGTGCAGAAGGGGATGT

XtKCNA3210 TAGATTAGTAAATAC--AACACA

++ ++* * *** **** ** * + + ++++++++ +++++ + ++

XtKCNA615 GGGATGg

GgKCNA3210 GGAATGt

XtKCNA3210

++ +++

12 of 21 positions (0.571) totally conserved.

39 of 67 positions (0.582) conserved in aligned fragments.

**Clique 478**:

| Xenopus tropicalis | KCNA1-5 | 136904 | (74) |
| --- | --- | --- | --- |
| Tetraodon nigroviridis | KCNA1b-5b | 20506 | (28) |
| Gasterosteus aculeatus | KCNA2a-10a | 18437 | (109) |
| Takifugu rubripes | KCNA2a-10a | 16443 | (102) |
| Tetraodon nigroviridis | KCNA2a-10a | 18927 | (118) |
| Danio rerio | KCNA2b-10b | 58015 | (32) |

XtKCNA615 accCATAGGTACCTGCTGATTAATTGCCTGTGcccccCTCTTCACTGATCAAATCTACAG

TnKCNA615b

GaKCNA3210a tcgca---------------------------TCCTTGca--CTCTTATCCCTGCTGCGT

TrKCNA3210a TCTG-----TTCTCTGCTCGCGTCACATTTGCTC

TnKCNA3210a GTGGCTGCTGTTTTACTGTCTGTGTTCTTGTCTGCTCTCATCACATTTGCTG

DrKCNA3210b agtCATAAAAACCTGCTCATCGATTTCCTGTt

++~~ ~~~~~ ~ ~ ~ ~~~~ ~ ~ + + ++ + +

XtKCNA615 GtcAGTCTATGCTt

TnKCNA615b TTTCC

GaKCNA3210a G--AGTTTGATCTCA---CTTACCCTCTcttTTATTTGAGCCTCTCATGTTTCCTTTCCC

TrKCNA3210a T--AGTCTGGCCTCgttgCATTGCCTCTTGATTGTTTACCCCTCTCAGATTTCATCTCTC

TnKCNA3210a G--AGTCCATCCTCA---CATTCCCACTCGATTGTTTACTCCTCTCATATTTTAATTCTC

DrKCNA3210b

+++ ++ + + ++ ++ ++ +++ +++++++ +++ + +

XtKCNA615

TnKCNA615b ATGAATTCCGGCCTGTGCTCATa

GaKCNA3210a ACACATTTTGTCCTGTGCTCATg

TrKCNA3210a ACACATTTTCTttgg

TnKCNA3210a ACACACATTGT

DrKCNA3210b

+ + ++++++++

No position present in all sequences.

46 of 143 positions (0.322) conserved in aligned fragments.

**Clique 479**:

| Takifugu rubripes | KCNA2b-10b | 22366 | (45) |
| --- | --- | --- | --- |
| Tetraodon nigroviridis | KCNA2b-10b | 21732 | (39) |
| Gasterosteus aculeatus | KCNA2b-10b | 26866 | (46) |

TrKCNA3210b GCCCTTGTATGCTCCTGATCCAACCTTGCATGTTTTTTGcagtga

TnKCNA3210b TGCCCTTGTATGCTCCTGACCCAACCTTGAATGCTATTT

GaKCNA3210b TGCCCTTGTGTGCCGCTGACCCAACCTTGTACGCTATTTGaaatgg

+******** *** **** ********* * * * ***+ + ++

30 of 38 positions (0.789) totally conserved.

35 of 46 positions (0.761) conserved in aligned fragments.

**Clique 482**:

| Gallus gallus | KCNA1-5 | 115431 | (55) |
| --- | --- | --- | --- |
| Xenopus tropicalis | KCNA1-5 | 137076 | (63) |
| Homo sapiens | KCNA2-10 | 100058 | (65) |
| Takifugu rubripes | KCNA2b-10b | 22929 | (59) |
| Gasterosteus aculeatus | KCNA2b-10b | 28289 | (28) |

GgKCNA615 c------TTACATTCAACTAAGGAAGa----CAATTTTTAAAACTAAAgca-------TA

XtKCNA615 cAGCCATTTCCATGTAGATTTAAAAGGTA-TCATTTTCTTTGACTGAAATCAATTGGCAA

HsKCNA3210 tAGCTATTTACATGTAAATACAAAAGGTAaTTAATTTTTAAAACTGAA------TAGCAA

TrKCNA3210b ATTTTCACTGACAGAAATCATTTTGCTA

GaKCNA3210b AACc-----------------------------------------

~~~ ~~++ +++ + ~~~ ~~ ~ ~ ~~~ ~~ ~~ ~ ~~ ~~ ~

GgKCNA615 TGc-ATGGATTct

XtKCNA615 Tagt

HsKCNA3210 gc-----------------ACAAACTCA

TrKCNA3210b TGgaATGGATTGATTATTTACCCTCTCAgcc

GaKCNA3210b -------GATCGATTGTTTACCCTCTCggtc

~~~~~~ ~~ ~~~++ +++ + +

No position present in all sequences.

13 of 91 positions (0.143) conserved in aligned fragments.

**Clique 484**:

| Takifugu rubripes | KCNA1a-5a | 7139 | (51) |
| --- | --- | --- | --- |
| Tetraodon nigroviridis | KCNA1a-5a | 18270 | (33) |
| Oryzias latipes | KCNA1a-5a | 9132 | (59) |

TrKCNA615a GTCACACTAGCATGATT-ACGGACCTTCCATTTTCGTACCGGCGTGCAtgag

TnKCNA615a gGCGAGCGTCACACAAGCATGAAA-ACACACCTT

OlKCNA615a aGCGAGCTTCACACGAGCATGATTtAGAGACTTTCCATTTTGATATTGGCATGCAcaag

++++++ ****** ******* * ** **+++++++ ++ +++ ++++ ++

18 of 26 positions (0.692) totally conserved.

42 of 59 positions (0.712) conserved in aligned fragments.

**Clique 487**:

| Gallus gallus | KCNA1-5 | 115515 | (46) |
| --- | --- | --- | --- |
| Xenopus tropicalis | KCNA1-5 | 88757 | (34) |
| Takifugu rubripes | KCNA1a-5a | 7210 | (364) |
| Tetraodon nigroviridis | KCNA1a-5a | 18348 | (378) |
| Oryzias latipes | KCNA1a-5a | 9217 | (375) |
| Gallus gallus | KCNA2-10 | 50178 | (62) |
| Xenopus tropicalis | KCNA2-10 | 136649 | (41) |
| Oryzias latipes | KCNA2a-10a | 42087 | (45) |

GgKCNA615

XtKCNA615

TrKCNA615a AAGGACGTTCTTTTGGATACCTGCCTCCATCctGCCCCTCCCATCCGCGCTCACAGGGAC

TnKCNA615a GGACGTTCTTTTGGATACCCGCCTCCATCtgGCCCCGCTCATCTGCTCTCACGCTGAC

OlKCNA615a AAGGATGTTGTTTTGGATGTCTGTCTTCATtcccCCTGGCACATATGTTTACATTTTGAG

GgKCNA3210

XtKCNA3210

OlKCNA3210a

+++++ +++ ++++++++ + + ++ +++ ++ + +++ + ++ ++

GgKCNA615 TGCAAGAGagcg------------------------------------------

XtKCNA615

TrKCNA615a TGCAC-CGCAGGAGCATTTACTTTaaAAACGC--AAACATTTACAAGTATAAAAGTTGAA

TnKCNA615a TGCAaaCGCAGGAACGTTTACTTTC-AAATGC--AAACATTTAGAAATATAAAATTTGAA

OlKCNA615a TGCAC-TGGACGAGGTTTTAATTTG-AAAacgcaAATCATTTAGAAGTATAAGAAATGAA

GgKCNA3210

XtKCNA3210 GC--AAA-----------------------

OlKCNA3210a

++++ + + ++ ~~ ~~~ ~~~ ~~ ~~~~~~ ~~ ~~~~~ ~ ~~~~

GgKCNA615 ----------------------------AATCAGATAATAGTAAGAAAG-TATTAACATG

XtKCNA615

TrKCNA615a TGCTGAACCTTAAAAGA------ACATAAATGAGATCATAGCATGATAG-AACTAACCTG

TnKCNA615a TGCTGAACTTTAAAAGA------ACATAAAAGAGATCATAGCATGATAG-AAGTAAGCTG

OlKCNA615a TGTAAAATTTTAAGGGAaacaatACATAAATGAGATCATAGCATGAAAAAAAGTTACCTG

GgKCNA3210

XtKCNA3210 ------------------------------------------------------------

OlKCNA3210a ACCACAGCGTCAGAAAAAGCCATTTG

~~ ~~ ~~~~ ~~ ~~~~~~~ ~~~ ~ ~~ ~ ~ ~ ~ ~ ~~

GgKCNA615 CAG

XtKCNA615

TrKCNA615a CATTATGGACTATCGACTGAGGCACTTTTGTATCCTGGTATTTACTG-------CATGAC

TnKCNA615a CATTATGGACTATCGACTGAGGCACTTTTGTATCCTGGTATTTACTG-------CATGAC

OlKCNA615a CATTATGGCCTTTCAGCTAAGGtACTTTTGTATCCTGGTATattaaatgcatgaCATGAG

GgKCNA3210

XtKCNA3210 ------------------------------------------------------------

OlKCNA3210a CAGTGAAGACTTTGACCTG

~~ ~ ~ ~~ ~ ~~ ~~~ ~~~~~~~~~~~~~~~~~~ ~ ~~~~~

GgKCNA615

XtKCNA615

TrKCNA615a TATACAGCTTGTGGTGACGCAGACACTCTGCAGTGTCCTGTGCTCAAGGAAACTAA-GGA

TnKCNA615a TATACAGCTTGTGGTGACGCAGGCACTCTGCAGTGTCCTGTGTTCAAGGAAACTAAaGGA

OlKCNA615a TATACAGCCTGTTGCAATTCAAGCACTTTGCAGTGCCCTAACATCAGGGAAACTGA-GGA

GgKCNA3210 TGGTGACCAAGACACCATGCAGTAgtGT---TGGAAGAACCAG------

XtKCNA3210 -------------------------------------GT---CTCAAGGACCCG------

OlKCNA3210a

~~~~~~~~ ~~~ ~ ~ ~ ~~~ ~~~~~~ + + + + ~ ~~~

GgKCNA615

XtKCNA615

TrKCNA615a CAACAAATTCTGTGTGTTTCTGAATTGCAATCAAATATATCCAGACCCATCGGGAGGATG

TnKCNA615a AAACAAACTCTGTGTGTTTCTGAAATGCAATCAAATATATCCAGACCCATCATGAGGATG

OlKCNA615a AATCAAAa-----GTCTTTCTGAATTGTTATCAAATATATCGAAAGCCAcctTGAGGATG

GgKCNA3210 ----------TCTGAGCAGCTGAAGTCTCCCa

XtKCNA3210 ----------AATAGGCAGCTGAAATCTGCCc

OlKCNA3210a

~ ~~~~ ~~ ~ +++++ + +++++++++ + + +++ + +++++++

GgKCNA615

XtKCNA615 AGTTTTTAAGTTCTAAGGAATCAAAAAACctaac

TrKCNA615a CAGAGTCTCTAATTTATCActa

TnKCNA615a CAGAGTTTCTAATTTACAAGTAATCAAAGAACatgat

OlKCNA615a CAGAGTTTCTAATTTAAAAGTAg

GgKCNA3210

XtKCNA3210

OlKCNA3210a

++++++ + +++ ++ + + +++++ +++ + +

No position present in all sequences.

98 of 397 positions (0.247) conserved in aligned fragments.

**Clique 489**:

| Takifugu rubripes | KCNA1a-5a | 7593 | (80) |
| --- | --- | --- | --- |
| Tetraodon nigroviridis | KCNA1a-5a | 18730 | (81) |
| Oryzias latipes | KCNA1a-5a | 9614 | (102) |
| Gallus gallus | KCNA2-10 | 40311 | (23) |

TrKCNA615a TCAAAAAGGC---ATATAAGCATATTAAGCACCGATTACATTATTTAGTCAGTAGTGCAG

TnKCNA615a TCAAAAAGGC---ATATAAGCATATTAAGCACCAATTACATTATTTAGTCAGCAGTGCAG

OlKCNA615a TCAGAAATGCataATATAACCACGGTAAGCATCAGTTAttgta-TTAGTCAATAGTTCAG

GgKCNA3210

+++ +++ ++ ++++++ ++ ++++++ + +++ ++~+++++++ +++ +++

TrKCNA615a TGCATGGGCATTCTTTGAT-AAGG

TnKCNA615a TGCATGGGCATTCTTTGAT-AAGGa

OlKCNA615a TGCATGGACCTTTTTTGAacAAGATGTTATGCCATCTCCACCt

GgKCNA3210 AAGGTGCTATGTCCTCTCCACCa

+++++++ + ++ +++++ *** + ++++ + ++++++++

3 of 4 positions (0.750) totally conserved.

73 of 103 positions (0.709) conserved in aligned fragments.

**Clique 495**:

| Homo sapiens | KCNA1-5 | 136261 | (197) |
| --- | --- | --- | --- |
| Gallus gallus | KCNA1-5 | 116015 | (157) |
| Xenopus tropicalis | KCNA1-5 | 76255 | (205) |

HsKCNA615 cCTTTTTCATTTGCATTCACCAAAAGTGCACTCCTcc---ATTTATTAACTATTTTATTA

GgKCNA615 ATTCACCAGAAGTGCACTACTta---ACTTGCTCAATTGTTGACTA

XtKCNA615 tCTTTTTAATTTGCATTCACCTAAAGTGCATTCCTaatttATTGAAAACCTTTTTAATTA

++++++ ++++++******* ******* * ** * * * ** * **

HsKCNA615 GTAAa-TAAAGTACTGTATTTAAGTGCA------TATGTTAGTCAGATGGGAACAATAAC

GgKCNA615 GTACTTTAAAGTg---------------------TATGTTAGTCAAATGGGAACAATAAC

XtKCNA615 GTAATTTACAGTACTGTATGTAAATGCAtatgacTATGTTAGACGAACTGGAACAATAAC

*** ~** *** ~~~~~~ ~~~ ~~~~ ******** * * ***********

HsKCNA615 TTTTTGGAGCTCAAAGCATGTTCTCTTATTCAGCATTATGG--CCTATTTGACTAAGATG

GgKCNA615 TTc-TGGAGATCAAAGCATGTTCAAATATTCAGCATTATGG--CCTATTTGAATAACACA

XtKCNA615 TTTTTTGAagatcAAGCATGTTTAAATACCCAGCATTATGGggCCTATTCGACTAAGGTG

** ~* ** ********* ** *********** ****** ** ***

HsKCNA615 TACCTTGAATTAATTAATGcatgatttca

GgKCNA615 TAACTTAAATTAATTAAT

XtKCNA615 TAACCAGAAGTAATGCATGatatca

** * ** **** **+

103 of 156 positions (0.660) totally conserved.

116 of 209 positions (0.555) conserved in aligned fragments.

**Clique 496**:

| Homo sapiens | KCNA1-5 | 136554 | (100) |
| --- | --- | --- | --- |
| Gallus gallus | KCNA1-5 | 116260 | (97) |
| Xenopus tropicalis | KCNA1-5 | 76516 | (57) |

HsKCNA615 TTTCCTGCC--TTTGCTCAGGGAAATAcca-GGTTTTTGTGCAGGTATAGGCGGAGAGag

GgKCNA615 TTCCCTGC--ACTGCTCAGGAAAACATGTTGGCTTTTGTCCAGGCATTGTCAGAAAGgg

XtKCNA615 TTTCCCGCagtAATCCTCAGGGAAATATGTTATCCACTTTCCAGGCATAGT---------

+**** * ****** *** * ~ * * **** ** * ~ ~~ ~~ ~

HsKCNA615 gaccaATATgCCCATCCCTTAAAGGGAA-GCCATGTGAAAAACT

GgKCNA615 at---ATAT-CCCATCTCTTAAAGGGAAaGCTATATGGAAAACT

XtKCNA615 ----------------TCTAAA

~~~~ ~~~~~~ ** **++++++ ++ ++ ++ ++++++

28 of 53 positions (0.528) totally conserved.

47 of 104 positions (0.452) conserved in aligned fragments.

**Clique 499**:

| Homo sapiens | KCNA1-5 | 136771 | (272) |
| --- | --- | --- | --- |
| Gallus gallus | KCNA1-5 | 116416 | (259) |
| Xenopus tropicalis | KCNA1-5 | 76714 | (120) |
| Takifugu rubripes | KCNA1a-5a | 7730 | (84) |
| Tetraodon nigroviridis | KCNA1a-5a | 18900 | (63) |
| Oryzias latipes | KCNA1a-5a | 9725 | (136) |
| Homo sapiens | KCNA2-10 | 80211 | (38) |
| Xenopus tropicalis | KCNA2-10 | 138686 | (39) |
| Takifugu rubripes | KCNA2b-10b | 22737 | (74) |
| Gasterosteus aculeatus | KCNA2b-10b | 28086 | (20) |

HsKCNA615 GCATGTTTTTTGGTGCATTCTTAGGATGTAAATGAAAatgtTTCTCTATTATATGCATCC

GgKCNA615 GCATGCTTTTTGGTGCATTCTCAGAATGTAAATGAAAc---TTATCTATTATATGCATCC

XtKCNA615 GCATGATTAGGGTTGCAGTATCAGAATGTAATgatcccttaACA------ATATGCATCA

TrKCNA615a tt----------------------------------------------------------

TnKCNA615a

OlKCNA615a gt---------------------------------------ACA------AGTTG-----

HsKCNA3210

XtKCNA3210 GCACGTA----------------------TTG-----

TrKCNA3210b

GaKCNA3210b

~~~ ~~ ~ ~~~~ ~ ~ ~~ ~ ~~~~ ~ ~~~~~~~ ~~~~~~

HsKCNA615 GAA-----GCAGAgctgatttttttttcT-------------------------------

GgKCNA615 AAAttgcaacagctggtnnnnnnnnnnnnnnnnnnnnnnnnnnnnnnnnnnnnnnnnnnn

XtKCNA615 AAAaaactGCAGAccatctgt-------T-------------------------------

TrKCNA615a ------------------------------------------------------------

TnKCNA615a

OlKCNA615a ------------------------------------------------------------

HsKCNA3210

XtKCNA3210 ------------------------------------------------------------

TrKCNA3210b

GaKCNA3210b

~~ ~ ~~~

HsKCNA615 -TTGCAGTCATTCTTTGAAGTCTGTAGAGACTTCAGCCCTC-------------------

GgKCNA615 nTTGCAGTCATTATTTGAGAT------------------------------TTTTTTTGT

XtKCNA615 -TTGCATCCATTATTTGAAATCCATAGAG--TTTTGCACTG-------------------

TrKCNA615a ----------TCACTTCATTTCCAGAGAA--TTa------------------------CT

TnKCNA615a TTCT

OlKCNA615a ------------GTTTAAAATCCAGAGAAAATTTTGAATTcatactttagaTTTTTTTGT

HsKCNA3210

XtKCNA3210 ------------CTTTCAGATCGAGGGAAAACCTTGAATTG

TrKCNA3210b

GaKCNA3210b

~~~~~ ~~~ ++ + +~ ~~ ~ ~ ~ ~~~~~~~ ~

HsKCNA615 ---CCCTTGAGGCTCCCTGAAGAAACTAAACCAATTGATTTAA--TAGTTGCtT------

GgKCNA615 attCCCCTAAAACTCACTGAGGAGACTTAACTGATTGATTTAAATCAATTtaaatcaatt

XtKCNA615 ---CTCTAG

TrKCNA615a C--------------------------------------TGACATTAGTTGC-T------

TnKCNA615a C--------------------------------------TGACATTAGTTGC-T------

OlKCNA615a G--------------------------------------TGACATCAGTTGC-G------

HsKCNA3210

XtKCNA3210

TrKCNA3210b

GaKCNA3210b

~ ~ ~ ~~~ ~~~~ ~~ ~~~ ~~~ ~~~~~~+ + ~~ + ++

HsKCNA615 -----------AGTGCCTTTATCCtgt----ACCCACAGtgAACTGTAGAAAGTGCCTCC

GgKCNA615 taaatttgtgcAGTGCCTTTATCttaCATTCTCTA------AAATGCTGAAAGTGCCT

XtKCNA615

TrKCNA615a -----------AGTGCCTTTATTC--CTTTCGCTAACAGGACCAG--TGAAAGTGCCT

TnKCNA615a -----------AGTGCCTTTATTC--CTTTCGCTAACAGGACCAG--TGAAAGTGCCTC

OlKCNA615a -----------AGTGCCTTTATCC--CTTccACCAACAGGACCAG--TGAAAGTGCCTCA

HsKCNA3210 GGACCCT--TGGTAGCTATCCA

XtKCNA3210

TrKCNA3210b AAAGTGCTTTG

GaKCNA3210b

+++++++++++ ~ ~ ~ + ~~~~ ~ + ++

HsKCNA615 TTAACACAGCTGAGAA---GTTAGGTAGCAaaa------GTGGGGAAGGGTTGGGGCACA

GgKCNA615

XtKCNA615

TrKCNA615a

TnKCNA615a

OlKCNA615a GAG----------TACTCAGTCGAGTGA

HsKCNA3210 GAG----------GACTCAGTCTGGTGt

XtKCNA3210

TrKCNA3210b TCAACACGGCTGTTAATGAATAAGGTGACAgGGTtactgGGGGGGGAGGGAGGAGAAACA

GaKCNA3210b TAACGAGTAAGGTGAC--GGTg

~~~~ ~~~~ + ~ + ++ +~ ~ + ++++ ++++ + + +++

HsKCNA615 GAc

GgKCNA615

XtKCNA615

TrKCNA615a

TnKCNA615a

OlKCNA615a

HsKCNA3210

XtKCNA3210

TrKCNA3210b AAt

GaKCNA3210b

+

No position present in all sequences.

44 of 363 positions (0.121) conserved in aligned fragments.

**Clique 508**:

| Homo sapiens | KCNA1-5 | 138241 | (51) |
| --- | --- | --- | --- |
| Gallus gallus | KCNA1-5 | 118039 | (36) |
| Takifugu rubripes | KCNA2a-10a | 14349 | (29) |

HsKCNA615 ACGGCACCAAGACTGACTTTGGGTATTTGGTATATTTACCGTGGTTacact

GgKCNA615 tg----------------TTGtag-TTGGTTATACATACCTTAGTTtaatgat

TrKCNA3210a ACTGCACCCAGAGTGACTTTAGCCATCTc

~~~~~ ~~~ ~~~~~** ~* ++++ ++++ + +++ +

3 of 12 positions (0.250) totally conserved.

16 of 53 positions (0.302) conserved in aligned fragments.

**Clique 511**:

| Homo sapiens | KCNA1-5 | 138523 | (59) |
| --- | --- | --- | --- |
| Gallus gallus | KCNA1-5 | 118414 | (72) |
| Xenopus tropicalis | KCNA1-5 | 78426 | (81) |

HsKCNA615 tgTAATGTGTTGAAATGTCTTTGTAGACCTGAAGGTGCACTTAACaaAACTGCCTATTA

GgKCNA615 GCTGAAATTTTTTCATGGGCCTGAAAGTGCACTTAACc-AACTGCCTGTTCT

XtKCNA615 tcTACTGTGCTCAAATGTCTTTGTAGACCTGAAGGTGCACTTGAt--AAATGCCTGTTAT

+ ++ +++* * **** * ** * * ****** ******** * ** ***** ** +

HsKCNA615

GgKCNA615 CACTATTAA--GCATCTTTCTTt

XtKCNA615 TACAATAAAcgGAATATTTTTTa

++ ++ ++ + ++ +++ ++

35 of 49 positions (0.714) totally conserved.

56 of 83 positions (0.675) conserved in aligned fragments.

**Clique 515**:

| Gallus gallus | KCNA1-5 | 121527 | (65) |
| --- | --- | --- | --- |
| Takifugu rubripes | KCNA2b-10b | 12095 | (77) |
| Gasterosteus aculeatus | KCNA2b-10b | 15964 | (62) |

GgKCNA615 caTTTTTTTTGCAGTATGTTGGTTTGtAATTAATAAATAA--CAGCTTTTAATGGACTGT

TrKCNA3210b tgTATTTTTCACAGTTTTTTcaccc--AATAATCGAAAAA--CAGCTTCTAAGGAATCGT

GaKCNA3210b tc-------------ATGTCGTCATG------ATAAATAcagCAACTTTTAAGGATTTGT

~ ~~~~~ ~~~~ * * ~ ~~~ ~ ** * ** *** *** * **

GgKCNA615 GTATGGc

TrKCNA3210b GTATGGAATAATGCTTCCCCC

GaKCNA3210b GTATAGAACAATGCCCCCCCC

**** * + +++++ +++++

21 of 45 positions (0.467) totally conserved.

32 of 81 positions (0.395) conserved in aligned fragments.

**Clique 516**:

| Homo sapiens | KCNA1-5 | 140323 | (193) |
| --- | --- | --- | --- |
| Gallus gallus | KCNA1-5 | 121597 | (224) |
| Xenopus tropicalis | KCNA1-5 | 82204 | (160) |

HsKCNA615 tGACATGCCTCTAATAGTAAAGATAAAGTATTACtg-------AGGTTAAAAATAAAAAT

GgKCNA615 aGACATACCTCTAATTTTAAAGGACAAATATTGCaagtgctaaATGTATAAACTAAAAAA

XtKCNA615 AAGATACAAAT

+++++ ++++++++ +++++ ++ ++++ + + ++ ** ** ***

HsKCNA615 TGAGTAGTATTAAT----TTAAAGTGCACCATCAGGACAACAAAC-CATTTAAGCTGAAA

GgKCNA615 TCTcca----------TATGAAAGTTCACCATCAGGACAACAAAC-CATTTAAGCTGAAA

XtKCNA615 TCTGTAGTTGGAATcgTATTTAAGTGCACATTCAGGGCAACAAAaaCATTTAAGCTtcta

* *~~ ~~~ ~~* **** *** ***** ******* ********** *

HsKCNA615 aaacgc------------------------------------------------------

GgKCNA615 cagctgtacttatttcc---ACCATTTacaaattgtgtacaaatattgaatcaaatatgt

XtKCNA615 cccgcagcctacaataacttACCATTTgagc-----------------------------

~~ ~ ~~~~~~~

HsKCNA615 -----------------------------------------TATTTTATTTCTtga----

GgKCNA615 atttcattgtctcaatacagTTCATTTTACTTCCATTAGTAAGGTTTGTTTATAATtt

XtKCNA615 --------------------TTATTTGTACATCAATCACCATATTTTATTTACAATacat

~~ ~~ ~~~ ~~ ~~ ~ ~ *** ***

HsKCNA615 -------GTTTGCCAGTTgcttccaccttgagttaaggacgtgtctcatcttcacctact

GgKCNA615

XtKCNA615 gaacttgGTTTGTCAGTT

+++++ +++++

HsKCNA615 gcgcattctcc

GgKCNA615

XtKCNA615

46 of 79 positions (0.582) totally conserved.

84 of 311 positions (0.270) conserved in aligned fragments.

**Clique 517**:

| Homo sapiens | KCNA1-5 | 140549 | (152) |
| --- | --- | --- | --- |
| Gallus gallus | KCNA1-5 | 121826 | (161) |
| Xenopus tropicalis | KCNA1-5 | 82387 | (131) |

HsKCNA615 CATTGAATGTTATGAATTGAGAACCTAATTGAtgcGCATAGTTTTCATCTATGCAATTTT

GgKCNA615 CATCAAATATTATAAGTTCTTGATCTATTTGAAATGCATAGTTTTCACCTATGCAATGCT

XtKCNA615 TTAAAATGACTACTTTTATCCagttct-----

+++ +++ ++++ + ++ + +++ ** * * ** **** * * * ~~ ~

HsKCNA615 ACTTGCTTCTGTCACTTTAcgATCTGTTCATATTTGGCATCAATTAAAGATACTTTTTAA

GgKCNA615 CCCTACTTCTGTCCTTGT-TCATCTGTTCATATTTGACATAAATTAAAGATAATTTTTAA

XtKCNA615 -CCAGCTTGTGTTATTTTATCATCTGTTCATATTTTGCATCTATTAAAGGTAATTTTTAA

* *** *** * *~ ************** *** ******* ** *******

HsKCNA615 --GGATCTTATCAAGGAATATCTTGACTGGTTAT

GgKCNA615 --GGATCCTACACAAAATTATTCTGTCTGTTTATTTTGTGGaat

XtKCNA615 aaGGATCTGATTCAGACCTATCTTGAGTGGTTATTGTCTTGgagt

***** * * *** ** ** ****+ + + + +

73 of 117 positions (0.624) totally conserved.

95 of 165 positions (0.576) conserved in aligned fragments.

**Clique 521**:

| Homo sapiens | KCNA1-5 | 145731 | (40) |
| --- | --- | --- | --- |
| Xenopus tropicalis | KCNA1-5 | 86507 | (84) |
| Xenopus tropicalis | KCNA2-10 | 74414 | (74) |

HsKCNA615 ATAGAGTAGGGATGATTGTTTTTCATTTAACTGCATTTTc

XtKCNA615 AATATATTGCAGCCTAGGAACATGAGGGATTTACTTt--TATTAGTTctaaggtggagaA

XtKCNA3210 AATTCATTACAGTATATAGATGTTTGGTTTTTATTTAACTGTTTGTT------------A

+++ +++ ** ** * * ** * ** ~~* **~ +

HsKCNA615

XtKCNA615 ACTGTTTTGGACTGGTTCAGGTCCAt

XtKCNA3210 ACTGTTAGGGAATGGTTCAGGTCCAg

++++++ +++ +++++++++++++

14 of 37 positions (0.378) totally conserved.

43 of 86 positions (0.500) conserved in aligned fragments.

**Clique 526**:

| Takifugu rubripes | KCNA2b-10b | 24125 | (38) |
| --- | --- | --- | --- |
| Tetraodon nigroviridis | KCNA2b-10b | 23711 | (32) |
| Gasterosteus aculeatus | KCNA2b-10b | 29592 | (38) |

TrKCNA3210b CCAGCTGACAGAGGGGGGAGCGGGGAGAATAAAagcca

TnKCNA3210b CCAGCTGACAGAGGAGGGAGCGGGGAGAATAA

GaKCNA3210b ctcaCTGGCGGAAGAGGGAGCAGGGAGAAGGAAgcgca

* *** * ** * ****** ******* *+ ++

22 of 32 positions (0.688) totally conserved.

25 of 38 positions (0.658) conserved in aligned fragments.

**Clique 544**:

| Gallus gallus | KCNA1-5 | 129853 | (34) |
| --- | --- | --- | --- |
| Takifugu rubripes | KCNA2b-10b | 12109 | (63) |
| Gasterosteus aculeatus | KCNA2b-10b | 15964 | (62) |

GgKCNA615 CTCAGtGATTAGTGTTTAAAAT

TrKCNA3210b TTTTTTCAcccaataatcGAAAAA-----CAGCTTCTAAG-GAATCGTGTATGGAAT

GaKCNA3210b tcaTGTCGTCAt---------GATAAAtacagCAACTTTTAAG-GATTTGTGTATAGAAC

+ + +++ ++ +++ ++ +++ * ** ** * **** * **

GgKCNA615 AAAGCCCCCTCt

TrKCNA3210b AATGCTTCCCCC

GaKCNA3210b AATGCCCCCCCC

** ** ** *

20 of 33 positions (0.606) totally conserved.

35 of 72 positions (0.486) conserved in aligned fragments.

**Clique 546**:

| Gallus gallus | KCNA1-5 | 130292 | (58) |
| --- | --- | --- | --- |
| Xenopus tropicalis | KCNA1-5 | 101881 | (56) |
| Oryzias latipes | KCNA2b-10b | 35892 | (24) |
| Takifugu rubripes | KCNA2b-10b | 14110 | (445) |
| Tetraodon nigroviridis | KCNA2b-10b | 15828 | (451) |
| Gasterosteus aculeatus | KCNA2b-10b | 18201 | (496) |

GgKCNA615 tgcac--ATATGCAGTC-------------------------------------------

XtKCNA615 TCC-------------------------------------TCTTTTTGGA

OlKCNA3210a AAAATGTATGTCCAACCTGTGTGG

TrKCNA3210b TGCAATATGCATGCATGTGTGGGTTGGacagcagttaTTTTCTTTTCTGA

TnKCNA3210b ATATGCAATTTGTATGCATGTGTGGGTTGAAGACCAActgTTTTCTGTTTTAT

GaKCNA3210b AAAATGAATATGCAATCTGTGTGCATGCGTGTGTTTGAGGACAAacattgt-TTTTTGGt

+ ~ ++ * *~ ~~ ~~ ~~~ ~~~ ~~~ ~ ~~ ~~ ~~~ ~~

GgKCNA615 --------------------------------------------AGAATATGCGCAGGTT

XtKCNA615 AA--CC-------CCATGGAATTGTATTGTTGAT-CCGAAAGAAACT---------GCTG

OlKCNA3210a

TrKCNA3210b TAGTCC-------CCAGAGCCTGTTATTGTGGCC-TCAAATGAAACT---------ATTG

TnKCNA3210b TTGTCC-------CCAGAACCTGTTAG-GTGGCC-TCCACTGAAACAGTATGTCCAAGTG

GaKCNA3210b tcgacccccccttCCAGAGACTGTTAGTGTGtgtaTCCGCTGAAACAATATGTACAGTTG

~ ~~ ~~~ ~ ~~ ~~~ ~ ~~~+ ~~~~ ~~ +

GgKCNA615 TCCCCCACTCTTCCTTCTCTTCCTCTg

XtKCNA615 Tg

OlKCNA3210a

TrKCNA3210b TTCTCCCGCATCCT------------------TCCCCCc--TCATAGAGGCCTGAAAACA

TnKCNA3210b TTCTCCCCTCTCTT------------------TCCCCCATATC--AGAGGCCTGAAAACA

GaKCNA3210b CACCCCCCCTCCCCCTCCCATCCACTtcatccTCCCTCATTTCATAAAGGCCTGAAAGCC

+ ++ ~~ ~ ~~~ ~~ ++++ + ~ ++~~+ ++++++++++ +

GgKCNA615

XtKCNA615

OlKCNA3210a

TrKCNA3210b TCCATCAATGTTGattagGTGCAGTGGATTGTAGATTTTGTTTCTATGAAATGAAATGTG

TnKCNA3210b TCCATCAATGTTG-----GTGCAGTGGATTGTGGATTTACTTTCTATGAAATGAAATGTG

GaKCNA3210b TCCATCAACGCTG-----GTACAGTGGATAACGGATTTTCTTTCTATGAAATGAAATGTG

++++++++ + ++ ++ ++++++++ +++++ ++++++++++++++++++++

GgKCNA615

XtKCNA615

OlKCNA3210a

TrKCNA3210b ATAGAATAATGTAAATATTAACCATTGGATGATGGAg--AAAAACTTGTACTATAAACTG

TnKCNA3210b ACAGAATAATGTAAATATTAACCATTGGATAATGGAACAAAAACCGTGTACTATAAAtga

GaKCNA3210b AGAGAATAATGTAAATAATAACCACTGGATGATGGATCAGAAACCACGTACTCTATACTG

+ +++++++++++++++ ++++++ +++++ +++++ ~~ +++ + +++++ ++ +

GgKCNA615

XtKCNA615

OlKCNA3210a

TrKCNA3210b TATGA--AGTCTGGATTTAAAACCTAGTATTATCTGCACTTTACAAGA-AAAAAGGACAA

TnKCNA3210b ta---------TGGATTTAAAACCTAGTATTATCTGCACTTTACAAGACAAcAAGGACAA

GaKCNA3210b TATGAgaAGTCTGGATTTAAAACGTAGTATTATCTGCACTTTACAAGACAATAAGGACAA

++~~~ ~~~~++++++++++++ ++++++++++++++++++++++++~++ ++++++++

GgKCNA615

XtKCNA615

OlKCNA3210a

TrKCNA3210b TCTATGGGCAAGAAAGA-CATGTGAAAatgAA--ATGCATATAATCCTTCCAAATGATCA

TnKCNA3210b TCTATGGGCAAGAAAGAtCATGTGAAATT-AA--ATGCATATAATCCTTCCAAATGATCA

GaKCNA3210b TCTGTGGACAAGAAATA-CATGTGAAATT-AAagATGCATATACTCCTTCCAAACGATCA

+++ +++ +++++++ + +++++++++ + ++ +++++++++ ++++++++++ +++++

GgKCNA615

XtKCNA615

OlKCNA3210a

TrKCNA3210b TAACCCTGAtAGAATATTATTAATAATGAAGCTAATAACAACTGGActggga--TTGTGT

TnKCNA3210b TAACCCTGAcAGAATATTATTAATAATGAAGCTAATAATAACTGGAa-AAGACGATGTGT

GaKCNA3210b TAACCCTGAaaaagTAATATCAATAATGAAGCCAATAATAAttcaaacAAAATGTTGTGT

+++++++++ + + ++ +++ +++++++++++ +++++ ++ + + + ~ +++++

GgKCNA615

XtKCNA615

OlKCNA3210a

TrKCNA3210b TTGGTGTCTTTATTGTGTGgta

TnKCNA3210b TGGGTGTTTTTATTGTGTtatttct

GaKCNA3210b TCAGTGTGTTTATTGTGTGtcact

+ ++++ ++++++++++

2 of 3 positions (0.667) totally conserved.

274 of 505 positions (0.543) conserved in aligned fragments.

**Clique 558**:

| Homo sapiens | KCNA1-5 | 169747 | (29) |
| --- | --- | --- | --- |
| Takifugu rubripes | KCNA2b-10b | 24687 | (98) |
| Tetraodon nigroviridis | KCNA2b-10b | 24274 | (47) |
| Gasterosteus aculeatus | KCNA2b-10b | 30277 | (82) |

HsKCNA615 TTGGACTGCAGAGTCGTCTTTTTTATTAg

TrKCNA3210b TTCAACTGCAGATTCATCCATTTTATTATCT-----AAAGCAAGTGAAAATAGAACCAAA

TnKCNA3210b aca------------------TTAATTAataa----AAAGTAAGTGAAAATAAAACCAAA

GaKCNA3210b agg----------------------TTATCTgccacAATGCAAGTGGAAATAGAACCAGA

~~~~~~~~ ~~ ~~ ~~~ ~*** ++ + +++++ +++++ +++++ +

HsKCNA615

TrKCNA3210b TGAGACTCttcacatgt-CAAGGCCTTTTGAATCCTGTGTACAg

TnKCNA3210b CGAGACTCc

GaKCNA3210b CGAGTCTCatggagtgcgCAGGGCCCTCGGTGTCAAGTGGACAc

+++ +++ + ++ ++ ++++ + + ++ +++ +++

3 of 7 positions (0.429) totally conserved.

47 of 104 positions (0.452) conserved in aligned fragments.

**Clique 560**:

| Homo sapiens | KCNA1-5 | 171301 | (47) |
| --- | --- | --- | --- |
| Xenopus tropicalis | KCNA1-5 | 112701 | (39) |
| Gasterosteus aculeatus | KCNA2a-10a | 23569 | (162) |
| Tetraodon nigroviridis | KCNA2a-10a | 23091 | (82) |
| Takifugu rubripes | KCNA2b-10b | 23959 | (157) |
| Tetraodon nigroviridis | KCNA2b-10b | 23556 | (148) |
| Gasterosteus aculeatus | KCNA2b-10b | 29412 | (161) |

HsKCNA615 ccca-------------C------TGTT-----T-----TCAGAGTAAAAAAGTCA----

XtKCNA615 t---------------------TCAGGActtagTTTGGTAC-------------------

GaKCNA3210a CCAGATGGCCATATTTTCctagTCTGGA-----TTTGGCAT-------------------

TnKCNA3210a CCAGATGGACATATTTTC------TGTA-----TTTGGaTCAGACACAAAAAGGCActaa

TrKCNA3210b ga----------------------------------------------------------

TnKCNA3210b

GaKCNA3210b

~~~~ ~~~~~~~~~ ~~ ~ ~~~~~ ~~~ ~~~~~~ ~~

HsKCNA615 ------------------GATGgt------------------------------------

XtKCNA615 ------ATTTCCGTGAATGATGTAt

GaKCNA3210a ------ATTTCTGTGAATGA--TACAGCTTAATCCCCAATGTGGAGGAGCAAAGGCAGGC

TnKCNA3210a aaaatgATTCCTGTAAATGAgccgga-CTTAATt

TrKCNA3210b ------------------GCTGTAAAATATAATCCCCCAGGTGAAGGTGGAATGGATGAT

TnKCNA3210b ATAATCCCCCAGGTGAAGGTGGAATGGATGAT

GaKCNA3210b GAAGAA--TACACCATAATCCCCAAGGTGAGGGCGGATTGGATGAT

~~~ ~ ~~ ~~ ~ ~~~~ ~~~ ~ ~~~ ~~ ~ ~ ~~ ~

HsKCNA615 ------------------------------------------------------------

XtKCNA615

GaKCNA3210a TGG---AGTGatgacggcccgggtGTGTGTTTCACTGCttttgAGGTAAAATGATATGAC

TnKCNA3210a

TrKCNA3210b ATG---TGTGG-------------ATGT-TTATACTGC-----AGGAAGGAATAGAAAAT

TnKCNA3210b AGG---TGTGG-------------GTGTTTTGTACTGC-----AGGAAAGAGTAGAAAAC

GaKCNA3210b cccaaaTGTGG-------------GTGC-CTACTCTGC-----AGGAAAGAGGggg-AAA

~~~ ~~ ~ ~~~~ ~~~ ~ ~ ~

HsKCNA615 ---ATT---------------------------------TTGGTT---------------

XtKCNA615

GaKCNA3210a AAcATT---------------------------------TTGGTT---------------

TnKCNA3210a

TrKCNA3210b GAGTGGGACCCGCTCTtctgtccACACATCAGGCAGCGATTGGTTCCCATGGAaCAAATC

TnKCNA3210b GAGTGGGACCCGCTCTCCCATCGACACATCAGGCAGCGATTGGTTCCCATGGAGCAAATC

GaKCNA3210b GAGTGGGTCCCACTCTGCCTCCGTCACGTCAAACAGTGATTGGCTCCTGCAGGGTAAATC

~ ~ ~~~ ~~~~ ~ ~ ~~~ ~~~ ~~~ ~~++++ +~~ ~ ~~~~~

HsKCNA615 -------AGAGA

XtKCNA615

GaKCNA3210a -------AAAGc

TnKCNA3210a

TrKCNA3210b TCCTATCAGAGAGCc

TnKCNA3210b TCCTATCAGAGAGCTac

GaKCNA3210b TCCTATCAGGGAGCTtc

~~~~~~~+ + ++ +

No position present in all sequences.

10 of 257 positions (0.039) conserved in aligned fragments.

**Clique 561**:

| Homo sapiens | KCNA1-5 | 171471 | (38) |
| --- | --- | --- | --- |
| Gasterosteus aculeatus | KCNA2a-10a | 21151 | (28) |
| Oryzias latipes | KCNA2a-10a | 36785 | (38) |

HsKCNA615m, TCTGCTTGTGTGTGGAATGACACACACCCATGGTTCTa

GaKCNA3210am, GCCTGCAGAGGGAAGCCTATCCATACTg

OlKCNA3210am, TCTGCTAGCCTGCAGAGGGACACCTCCCCATGTTTTTc

++++++ * ** ** ** * **** * +

13 of 28 positions (0.464) totally conserved.

20 of 38 positions (0.526) conserved in aligned fragments.

**Clique 563**:

| Homo sapiens | KCNA1-5 | 179476 | (22) |
| --- | --- | --- | --- |
| Takifugu rubripes | KCNA1a-5a | 6935 | (119) |
| Tetraodon nigroviridis | KCNA1a-5a | 18095 | (87) |
| Oryzias latipes | KCNA1a-5a | 8933 | (119) |

HsKCNA615 CATCTTCTAGAGGAAGCATGGg

TrKCNA615a CTCCCCCACCCCGCAAAGGATCtactgttcattAGCGCACCTTATAGAGGGAAGACCGGA

TnKCNA615a CGACGCACCTTCTAGAGGGAACACGGTA

OlKCNA615a CTCCCCACCCCCATAAACGATCagccactcagCAAAGCACCTTACAGGAGGAAGACTGAT

++++++ ++++ +++ ++++ + +++ +** *** ** * * * *

HsKCNA615

TrKCNA615a CCCTCCTGCTGGGATGTAAACAGATGGTGATCTACATGCTTCACGGACATCCGCATTGC

TnKCNA615a ACCTCCTGCTGGGATGTAAACAGATGGCGATCTACATGCTTCACGGACATCCGCgtcgc

OlKCNA615a CCTTCTTctttGGATGTAAACAGATGGTGGTCTGCTTGCTTCACAGACATCtaCACTGC

+ ++ + + ++++++++++++++++ + +++ + ++++++++ ++++++ + ++

11 of 22 positions (0.500) totally conserved.

76 of 119 positions (0.639) conserved in aligned fragments.

**Clique 575**:

| Homo sapiens | KCNA1-5 | 195169 | (76) |
| --- | --- | --- | --- |
| Xenopus tropicalise | KCNA2-10 | 133568 | (36) |
| Takifugu rubripes | KCNA2b-10b | 24256 | (225) |
| Tetraodon nigroviridis | KCNA2b-10b | 23847 | (216) |
| Gasterosteus aculeatus | KCNA2b-10b | 29766 | (227) |

HsKCNA615

XtKCNA3210 c-----------------------------------------------------------

TrKCNA3210b TGGCATCTCTGGTTTCGTgtcaGGTATTAAATTACCCAATGGGAGCTTTAATACAGCCAG

TnKCNA3210b TGGCATCTCTGGTTTCCTATAGGGTGTTAAATTACCAGATGGTAGCTTTATTACCACCAG

GaKCNA3210b TGGCATCTCTGGTTTCGTATAGGGTGTTGAATTACCCAATGGGAGCTTTAATAGAGCCAG

~~~~~~~~~~~~~~~ ~ ~ ~~~ ~~ ~~~~~~~ ~~~~ ~~~~~~~ ~~ ~~~~

HsKCNA615 GGATAAAGGTGAGTTTTAAGGGAAGAAGGcatgtgACG

XtKCNA3210 -------CCTAATTTTGTGTCTGAATGAGGCATGAATTGAAC

TrKCNA3210b ACACATTCTTGATTGTGAGTCTGGATGAGGGAGAGATTGAAGAGAAGAAGGGGGAGCACG

TnKCNA3210b ACACGTTCTTGATTGTGAGTCTGGATGAAGGAGAGATTGAAGAGAAGAAAGGGGAGCGCG

GaKCNA3210b AGGCACTCTTGATTGTAAGTCTGGATGAGGGAGAGATTGAAGGGAGGAAAGGGGGGAACA

~ ~ ~+ + +++ + ++++* ** * * ** ** ++ +++ + +

HsKCNA615 GCAc-----------------------------------------------------CAG

XtKCNA3210

TrKCNA3210b GCAGAGCCGCTGGGAAGGGTAA---AAAGGGCACAACAACTTCTGCAGAGCCGGG-ACAT

TnKCNA3210b GCAGAGGCGCTGAGAAGGGTAA---AGAGGGCACGGCAACTTCTGCAGAGCCAGCAACCT

GaKCNA3210b G-AGTGCAGCTGTGAGGcaagaaagAGAGGGCACAACAACTCCTGCAGAGCCGGGGACC-

+~+ ~ ~~~~ ~~ ~ ~ ~ ~~~~~~~ ~~~~~ ~~~~~~~~~~ ~ ~+

HsKCNA615 GGGTGAAAGAGGAGGAGATGAGGTTGCAGCa

XtKCNA3210

TrKCNA3210b GGGTGAGTGGTGCGAACATCTGCACACACCTGGATCTCAGCTTTACACT

TnKCNA3210b CGGTGAGTGtc----------GCACACATCTGGATTTCAACTTTACTCT

GaKCNA3210b GGGTGAGTGGTGCGGACATCTGCACACAGCTGGATATCAACTTTGCATT

+++++ + ~ ~ ~ ~~ + ++ + ++++ +++ ++++ + +

9 of 20 positions (0.450) totally conserved.

52 of 229 positions (0.227) conserved in aligned fragments.

**Clique 577**:

| Gasterosteus aculeatus | KCNA2a-10a | 25213 | (98) |
| --- | --- | --- | --- |
| Oryzias latipes | KCNA2a-10a | 45397 | (77) |
| Takifugu rubripes | KCNA2a-10a | 22785 | (98) |

GaKCNA3210a tgGCTTGCTGGTATCTGTGTTGACCTGACAGAAGAcATCC-CTCGGCTTATAGCACTGAG

OlKCNA3210a TATCTGTGTTGATCCGTTGGAAAG-ATCC-TTCGGCTTATGGCACTGAG

TrKCNA3210a taGCTTGCTGGTATCTGTGCTGATCTGACAGAAGA-ATCCaCTCTGTTCACAATGCCAAG

+ +++++++++******** *** * * *** **** ** * * * * **

GaKCNA3210a CTagCCAGCCCGCATTAATCTGCTTAATAGGGTAAAAAa

OlKCNA3210a CTGCCCAGCCCGCATTAATCTGCTTAATAG

TrKCNA3210a CCGCCTCCTCTGTATTAATGTACTCGATAGGTCAAAAAc

* * * * ****** * ** ****+ +++++

45 of 77 positions (0.584) totally conserved.

61 of 99 positions (0.616) conserved in aligned fragments.

**Clique 578**:

| Gasterosteus aculeatus | KCNA2a-10a | 25399 | (60) |
| --- | --- | --- | --- |
| Takifugu rubripes | KCNA2b-10b | 21628 | (62) |
| Tetraodon nigroviridis | KCNA2b-10b | 21085 | (41) |
| Gasterosteus aculeatus | KCNA2b-10b | 26246 | (41) |

GaKCNA3210a CAAAAGGTCCAACATacaatggcgACCACAATGTGTTTACTAGTACC-CAG

TrKCNA3210b caccagttcCAAGTCATTCAAGATGA-------ACCTCCGTCTGTTGATTAGTGTG-CAG

TnKCNA3210b GTCATCCAAGATTA-------GCAGTTGTCTGTTGATTAGTGTGGCAG

GaKCNA3210b GTCATCCAAGATTA-------GCACTTGTCTTTTGATTAGTGAGGCcg

+++ * *** ** * * * ** * **** ~* *

GaKCNA3210a AGGAGAGAAg

TrKCNA3210b AGGAAAGAAa

TnKCNA3210b

GaKCNA3210b

++++ ++++

18 of 40 positions (0.450) totally conserved.

29 of 70 positions (0.414) conserved in aligned fragments.

**Clique 591**:

| Gallus gallus | KCNA1-5 | 147515 | (42) |
| --- | --- | --- | --- |
| Gasterosteus aculeatus | KCNA2a-10a | 26629 | (47) |
| Takifugu rubripes | KCNA2a-10a | 23487 | (72) |

GgKCNA615 CACACAGGTATAGTGTTTTCCCATCTCTGTGTCTGc---------TGTTAa

GaKCNA3210a TTTCTt--TGTGTTGCAAGAGTTGATGTCCAATGGGT

TrKCNA3210a CACACATACAAAGTGTTGACCCGTTTATGTGTTTGTTATCAGGAATGTTATCTTACAGAT

++++++ + ++++++ +++ * * * ~~* ** ~ ~~ ** * + + + +

GgKCNA615

GaKCNA3210a TACTCCCACTAc

TrKCNA3210a TTCTCCCACTAt

+ +++++++++

9 of 17 positions (0.529) totally conserved.

39 of 72 positions (0.542) conserved in aligned fragments.

**Clique 607**:

| Homo sapiens | KCNA2-10 | 122930 | (71) |
| --- | --- | --- | --- |
| Gallus gallus | KCNA2-10 | 56945 | (88) |
| Danio rerio | KCNA2b-10b | 74998 | (41) |

HsKCNA3210 ATGGGGCCCTGTTCCTGCTTGagg----------GTGACTTAGGGACCTTCTGACA

GgKCNA3210 aagcATGTGGCTCCGTTCCTGCTTGGAGAGGTTGCTTTGTGCCTTTGGGAAGTTTTAATT

DrKCNA3210b gagtgTGTGTTTATGTGTGTGCGTGGAGATGTTGCTCTGTt

++ ** * ** *** ** *~ ~~~~~~ ~** +++ ++++ ++ + +

HsKCNA3210 CTCTgg---TTTGGAAGGACAGAGAGGt

GgKCNA3210 CTGTctgcaTTTAAAAGGAGACAGAAGa

DrKCNA3210b

++ + +++ +++++ + +++ +

13 of 27 positions (0.481) totally conserved.

42 of 88 positions (0.477) conserved in aligned fragments.

**Clique 616**:

| Gallus gallus | KCNA1-5 | 165980 | (48) |
| --- | --- | --- | --- |
| Xenopus tropicalis | KCNA1-5 | 122825 | (45) |
| Oryzias latipes | KCNA2a-10a | 47080 | (21) |

GgKCNA615 TTTCTTAACAGCTGGACAAAACAGttccgatgGAACTACAAATGCATa

XtKCNA615 gTTTCTGAGCAGATGAGCAAAACTGgcct----GATCTGCAAATCTATt

OlKCNA3210a aTTTCTGAACAAATGAGCAAc

***** * ** ** *** ++ + + ++ ++ +++++ ++

13 of 20 positions (0.650) totally conserved.

28 of 49 positions (0.571) conserved in aligned fragments.

**Clique 618**:

| Gallus gallus | KCNA1-5 | 170480 | (28) |
| --- | --- | --- | --- |
| Homo sapiens | KCNA2-10 | 72658 | (72) |
| Gallus gallus | KCNA2-10 | 29834 | (70) |

GgKCNA615 AGTCTGGTGGCACCTGACT

HsKCNA3210 CTGCAGTGTCGATGTGAGGGaaGCCCGGGGGAGGGAcAGTTAGTATGACCGAATATGAGT

GgKCNA3210 CTGCAGTATGCCAGTGAGAG--GCCCAGGGGAGGGAtAATTAGTCTGGCGGCACATTATT

+++++++ + +++++ + ++++ +++++++++ + ++*** ** * * * * *

GgKCNA615 TGTCAAATT

HsKCNA3210 TGTCAAGTTTCA

GgKCNA3210 TATTAAGTCTTA

* * ** * + +

15 of 28 positions (0.536) totally conserved.

47 of 72 positions (0.653) conserved in aligned fragments.

**Clique 621**:

| Gasterosteus aculeatus | KCNA3a-10a | 27584 | (83) |
| --- | --- | --- | --- |
| Oryzias latipes | KCNA3a-10a | 49350 | (62) |
| Takifugu rubripes | KCNA3a-10a | 24678 | (75) |
| Tetraodon nigroviridis | KCNA3a-10a | 24803 | (70) |

GaKCNA3210a CCACAGCTCTAGTTTCTACTCTAGCATAAGGTTACCCAATGTCG-TCAGTTATAGGTCAG

OlKCNA3210a CCACGGCTTCAGTTTCTACTC-AGTGTAAGCTTATCCAGTTTCA-GCCATGAAAGACAAG

TrKCNA3210a AGGTCTGGTTTCTAGTTCAGCATTGGGTTACTCCATGTCctCTGGTGACCAATGAG

TnKCNA3210a AGGTCTTGTTTCTAGTTCAGCATAAGGTTACTCTGTGTCG-CTTGTGACAAATGAG

++++ * * ******* * ** * * *** * * ** * * **

GaKCNA3210a TCGTTCTTATATAAATTGAGGAAG

OlKCNA3210a TCAT

TrKCNA3210a TCATTCTGATGCCAATTGg

TnKCNA3210a TCATTGTGATGCCAATCGAGGACG

** *+ + ++ +++ + +++ +

28 of 58 positions (0.483) totally conserved.

44 of 84 positions (0.524) conserved in aligned fragments.

**Clique 623**:

| Gasterosteus aculeatus | KCNA3a-10a | 27753 | (103) |
| --- | --- | --- | --- |
| Oryzias latipes | KCNA3a-10a | 49515 | (99) |
| Takifugu rubripes | KCNA3a-10a | 24845 | (24) |
| Tetraodon nigroviridis | KCNA3a-10a | 24955 | (40) |

GaKCNA3210a tagacttctTGCTAACGAAGGTAAGGTGTGTGCTGCTTGCTGGTTTTTCTGtctatgagc

OlKCNA3210a ttaatatct--CGAATGAAGGTAAGTTAACTGTAGCCTGGATATTTTTCTGc--------

TrKCNA3210a TGCTAAACAAGGTAAGGTGTGTGT

TnKCNA3210a cctgc----TGCTAAACAAGGTAGAGTGCATGTTTGTTTCTTGg

~~~~~* ** ****** * ** + +++++++

GaKCNA3210a ttgTCTGTGCAGTTTAGTTCCCATTTTTCCAGggtaatgggat

OlKCNA3210a ---TATGGGCATTTTTGCACCCTTTTGTGCTGtttctctagttcattatgat

TrKCNA3210a

TnKCNA3210a

+ ++ +++ +++ + +++ +++ + + + + + +

12 of 22 positions (0.545) totally conserved.

42 of 112 positions (0.375) conserved in aligned fragments.

**Clique 628**:

| Gallus gallus | KCNA3-10 | 57133 | (23) |
| --- | --- | --- | --- |
| Gasterosteus aculeatus | KCNA3a-10a | 28534 | (100) |
| Oryzias latipes | KCNA3a-10a | 50330 | (109) |
| Tetraodon nigroviridis | KCNA3a-10a | 25377 | (65) |

GgKCNA3210

GaKCNA3210a tAAATGTGACCTCAGGTGTCTGGgg----CAAGCttagCCCATGCTATCACTGTACggac

OlKCNA3210a cAAACGGGAATCCTGGTCTGTGGacggatCAAGC----CTCACGCTATCACTGTACAGTC

TnKCNA3210a CTGTCGCTGACCAACT

+++ + ++ + +++ + +++ +++++ + ++ +++ ++ +++ +

GgKCNA3210 CACAGCCCTGCTTTGCCTGTGCc

GaKCNA3210a acTTAATAATGCACTAATGTATGCGCAGCCCCAGTTACCCTGAG

OlKCNA3210a TGTTAATTATGCACTGATGTATGCACAGTCCCAGTTACCCTTCACTATCCTCc

TnKCNA3210a GGTTAATTATGC----ATGTATTCACAGCCCTAGTTTCCCTGTGCTGTCCTCa

+++++ ++++~~~ ++++++ * *** ** ** *** + +++++

11 of 21 positions (0.524) totally conserved.

63 of 113 positions (0.558) conserved in aligned fragments.

**Clique 630**:

| Homo sapiens | KCNA3-10 | 140571 | (37) |
| --- | --- | --- | --- |
| Xenopus tropicalis | KCNA3-10 | 119147 | (30) |
| Tetraodon nigroviridis | KCNA3a-10a | 17037 | (51) |

HsKCNA3210 CTGGCCTGCCATTGCACTCAAAAATCCTCCTCTCTTa

XtKCNA3210 TACCTTTCACAACTCCGGTCTGTTCAAAAt

TnKCNA3210a TACATTTCACAAATTTGGTCTGCTACAAAGCTCAAGAATCTTTCTTTCTTt

+++ ++++++++ + ** *** +++++ ++++ + ++ ++++

5 of 16 positions (0.312) totally conserved.

33 of 51 positions (0.647) conserved in aligned fragments.

**Clique 635**:

| Homo sapiens | KCNA3-10 | 144844 | (37) |
| --- | --- | --- | --- |
| Gallus gallus | KCNA3-10 | 30562 | (23) |
| Takifugu rubripes | KCNA3b-10b | 19815 | (39) |

HsKCNA3210 CATTGTCTCTCCTCCAGGCCGCAGCCCTC--CCAGGGAa

GgKCNA3210 TTG-----------GTCAGCAGTTTTCAGCCATt

TrKCNA3210b CACCGTCTCTGCTCTAGTCAGCAGCTCTCAGCCATGGAt

++ *~~~~~ ~~~ ~* * **** **~~*** ++

12 of 21 positions (0.571) totally conserved.

16 of 39 positions (0.410) conserved in aligned fragments.

**Clique 643**:

| Gallus gallus | KCNA1-5 | 176580 | (30) |
| --- | --- | --- | --- |
| Takifugu rubripes | KCNA2b-10b | 25059 | (89) |
| Tetraodon nigroviridis | KCNA2b-10b | 24648 | (74) |
| Gasterosteus aculeatus | KCNA2b-10b | 30623 | (139) |

GgKCNA615

TrKCNA3210b tg------------------C-AAGCAGAGTATCAGATGACAAGTTTGCTGCTGATGCAA

TnKCNA3210b GAGGAGGGGAAAAGACCAGTC-AAGCAGAGTATCAGATGACAAGTTTGCCGCTGACGCAA

GaKCNA3210b GAGAAAAAAAAAATAACTGTagAGGCACAGTATCGGATGACAACGTCTATGCTGATGCAA

~ ~ ~~~~ ~ ~ ~~ + +++ ++++++ ++++++++ + +++++ ++++

GgKCNA615 ACATTCT

TrKCNA3210b ACAGAAgt-GACAGAAAAATcag--------CAAAACTGTAGCGGGCTGTTAAACAa

TnKCNA3210b ACAGAAaccGACAGA

GaKCNA3210b ACgccagct----GAAAAATgcctctgcccaCAAAACTGTATCCTACTATAGAACATATT

++ + ~~~~+++++++ ++++++++++ + ++ + ++++ +

GgKCNA615 GTAACGTAGATATTTCCTCCCCt

TrKCNA3210b

TnKCNA3210b

GaKCNA3210b ATAACATAAATGCTCCCCCCCCc

++++ ++ ++ + ++ ++++

No position present in all sequences.

72 of 143 positions (0.503) conserved in aligned fragments.

**Clique 648**:

| Homo sapiens | KCNA1-5 | 197687 | (30) |
| --- | --- | --- | --- |
| Gallus gallus | KCNA1-5 | 185409 | (33) |
| Gallus gallus | KCNA2-10 | 46128 | (48) |

HsKCNA615 AGCATTTTAAGTTTGAGCTAAGAGGATgaa

GgKCNA615 ctaattCTGATCCCTGACAGCTCCTTGGGccct

GgKCNA3210 ctgctgCTGATCCATGGCAGCTCTTTAAACTCGAGTTTAGAGGATaag

++ + +++++++ ++ +*** ** ++ + +++++++ +

5 of 15 positions (0.333) totally conserved.

29 of 48 positions (0.604) conserved in aligned fragments.

**Clique 652**:

| Homo sapiens | KCNA1-5 | 199852 | (42) |
| --- | --- | --- | --- |
| Gallus gallus | KCNA2-10 | 57213 | (38) |
| Gasterosteus aculeatus | KCNA2b-10b | 22087 | (63) |

HsKCNA615 TTTATa--------TCGTGCCTGAAACGAAGGAGGAAAATGAGAGAA

GgKCNA3210 attCAACTTCCTGCTCTTTGCCTCCTTTTTGCCCTGAG

GaKCNA3210b accCCACTACTTGTTTATTGCCTCATTTCTTCCCTGAAAAGAAGGAAACAAATAGGTGGA

+ + +++ + ++ * * ~~~~~ ~~* ***** + ++++++ ++++ + + +

HsKCNA615 AAt

GgKCNA3210

GaKCNA3210b AAa

++

8 of 17 positions (0.471) totally conserved.

32 of 63 positions (0.508) conserved in aligned fragments.

**Clique 659**:

| Homo sapiens | KCNA1-5 | 211948 | (50) |
| --- | --- | --- | --- |
| Gasterosteus aculeatus | KCNA2a-10a | 18162 | (220) |
| Oryzias latipes | KCNA2a-10a | 33778 | (146) |
| Takifugu rubripes | KCNA2a-10a | 16146 | (197) |
| Tetraodon nigroviridis | KCNA2a-10a | 18658 | (250) |

HsKCNA615 tga---------------------------------------------------------

GaKCNA3210a gTGTCCATTAAAGATGGAATATTTGAATTGAATATGAACCCttacctcaatgTT---TTT

OlKCNA3210a c---------------------------------------------------TT---GTG

TrKCNA3210a gcaTCCATTACTGAGGATATATTTGTATCAAGTATCGACCCca--TCTCCTGGATGGTTT

TnKCNA3210a aTGTCCATTACTGAGGGTGTATTTGAATCAAGTATCGACCtcctgTCTCCTGGATGGTTT

~~~~~~~ ~~ ~ ~~~~~~ ~~ ~ ~~~ ~~~ ~~ ~~~ ~

HsKCNA615 ------------------------------------------------------------

GaKCNA3210a TATGTCCTGCTCGTCTCTTTAAGTGAATC---TTGTGCCTGTGtcacct-----------

OlKCNA3210a TCTCTCTCTCAGCTCCTCTTGAGTGACTgttgTGTTCTCTGTGGATAACTGGGAAAGCAA

TrKCNA3210a GGTGTCATGCTTCTCTCCTTAAGTGAAGC---TGCTCTCTGTGGTCTACTGAGAAAGCAA

TnKCNA3210a GGTGTCGCACTTCTCTCCTTAAGTGAAGC---TGCTCTCCGTGGTCTGCTGCAAAAGCAA

~ ~~ ~ ~~ ~~ ~~~~~ ~ ~ ~ ~~~ ~~ ~~~~~~~

HsKCNA615 ----------------------------------TACAGCAGatg-------------AA

GaKCNA3210a -----TTTGTTTGTTAAACCAGCAA----AGGGCAA--------AGTG----------TG

OlKCNA3210a AGTGATCTATTTATAAAACCAGCTAgaaaTGGAGTAAAGCAGtgAGTC----------AA

TrKCNA3210a AGCGGTCTATTTATACAGCGAGCGG----TGGAGAA--------AGTGAGGCAGCACAAG

TnKCNA3210a AGTGGTCTATTTATACAACTAGCTG----TGGAGAA--------AGTGAGACAGCCCAAG

~~ ~ ~ ~ ~~~ ~ ~ ~ ~~~ ~~ * ~~~~~ ~~ ~~ ~~~~ ~~

HsKCNA615 GGAGAGAAAAAGccagggtc--TTCTTCATTTTCAt

GaKCNA3210a GAAGAGAAACCAGAACAAAACGCTCTTGACTC-CTGGTTCAAagtAGAGTGGGAGACGTA

OlKCNA3210a GGGGAGAAAAAGGAGCAAAAGATTTCTGAC

TrKCNA3210a GAAGACAGcg------AAAACGTTCTCGACTC--AGGTTCAA

TnKCNA3210a GAAGAGAGAACAGAGCAAAACGCTCTTCACTCTCAGGTTTAAcacAGAGTGGAGCAAGAG

* ** * * * + ~~ +++ ++ +++++++ + +

HsKCNA615

GaKCNA3210a CAAACAGACTCTGTATCATCACCAT

OlKCNA3210a

TrKCNA3210a

TnKCNA3210a CAAACATTCTCTCCATCAATGCCAT

++++++ ++++ ++++ ++++

7 of 28 positions (0.250) totally conserved.

40 of 265 positions (0.151) conserved in aligned fragments.

**Clique 662**:

| Gallus gallus | KCNA1-5 | 186959 | (32) |
| --- | --- | --- | --- |
| Xenopus tropicalis | KCNA2-10 | 109963 | (86) |
| Gallus gallus | KCNA2-10 | 47234 | (98) |
| Tetraodon nigroviridis | KCNA2a-10a | 22312 | (42) |

GgKCNA615 TGACTGTTAAAGACTGAAGGTGATCACTGCTT

XtKCNA615 GAATGa--------GAAGTTGG---TTGCTGTAACATAGCATGTGGTTTTCTCTGATCT

GgKCNA3210 TGACTTTTGGGGTCTGAAGGTGGGCTTTGCTGATTCAATGAAGCTGGAGTTCCTTGCTTT

TnKCNA3210a cct-----------------------------------------------------CTTT

~ ~ ~ ~~~~~~ ~~ ~ ~~~~ ~~ ~ ~ ~~~ ~~~ ~~ + +

GgKCNA615

XtKCNA615 cttacaT--GCAAACCACATATGGAT--------GTTTTTccacattc

GgKCNA3210 TCCCCTT--GCAAACCAGATATAGATATGTGTGTGTATTT

TnKCNA3210a CCCCCTctcGCCAAACAGATACAGTGACGCGCGTt

+ ++ ++ ++ +++ + ~ ~ ~ ~~ + +++

0 of 2 positions (0.000) totally conserved.

17 of 108 positions (0.157) conserved in aligned fragments.

**Clique 663**:

| Gallus gallus | KCNA1-5 | 188168 | (20) |
| --- | --- | --- | --- |
| Takifugu rubripes | KCNA1a-5a | 7072 | (59) |
| Tetraodon nigroviridis | KCNA1a-5a | 18217 | (43) |
| Oryzias latipes | KCNA1a-5a | 9062 | (59) |

GgKCNA615 CCATGTCCCTTAGATGCAAg

TrKCNA615a g------CTGTGTTctgtcagatGGCCAAGTGATTCAACGCCGTGCCCTTTAGAGTCAAC

TnKCNA615a GGCCAAGTGATTCAACGCCGTGCCCTTTAGAGTCAAC

OlKCNA615a gctgtaaCTGTGTTatca-------CCAAGTGATTCAATGCCATGCCCTTTAGATGCAAT

+ +++++++ + ~~+++++++++++++ +** ** ** ***** ***

GgKCNA615

TrKCNA615a TCAAC

TnKCNA615a TCGACG

OlKCNA615a GCAGCG

+ ++

14 of 20 positions (0.700) totally conserved.

40 of 66 positions (0.606) conserved in aligned fragments.

**Clique 670**:

| Gallus gallus | KCNA1-5 | 194639 | (57) |
| --- | --- | --- | --- |
| Xenopus tropicalis | KCNA1-5 | 135322 | (67) |
| Oryzias latipes | KCNA2a-10a | 49940 | (86) |

GgKCNA615 cACC-----------------AacgAACTTC-ACTTTc----------------------

XtKCNA615 aacatgatccttactcttctgAT--AATATTTTCTTTTTtgg------------------

OlKCNA3210a ACC-----------------AT--AACTTCTACTTTTTcaaaatgagtcaccatttatc

** * ** * ~ **** ~

GgKCNA615 ---------ATCAGCAAGAACAGCAGTTTTTTGTTAGTCCCCTGCc

XtKCNA615 AGTATTTCTGAGGACAAAAACAATAAC

OlKCNA3210a AGTATATCTAACAATAAAAACAATAATTTATTGTTACTTTCCTGCa

~~~~~ ~~~ ** **** * ++ ++++++ + +++++

17 of 35 positions (0.486) totally conserved.

31 of 106 positions (0.292) conserved in aligned fragments.

**Clique 677**:

| Homo sapiens | KCNA1-5 | 214483 | (40) |
| --- | --- | --- | --- |
| Gallus gallus | KCNA1-5 | 198396 | (30) |
| Xenopus tropicalis | KCNA1-5 | 143484 | (71) |
| Homo sapiens | KCNA2-10 | 124272 | (31) |
| Takifugu rubripes | KCNA2b-10b | 21541 | (39) |
| Gasterosteus aculeatus | KCNA2b-10b | 26142 | (49) |

HsKCNA615 ACTAATGCACTTTCCTGAATGTCTG-

GgKCNA615 GAGAAGTTGCTAAGGCACTGTTGGGAATGc

XtKCNA615 CGTGTTTTTGCCAGAGAGAGAGAGAGAGGAGACTAATGTACTGTTGTGAATGTGTG-

HsKCNA3210 GAACATGTGTTTGCTAGAGAAAGAAAGg------------------------------AA

TrKCNA3210b AAAAAAGGTGTCATT------TAAT----------------CAAA

GaKCNA3210b GAAtGTGTGTTTGCAAAAGAAAGTGTGATT------TAAT----------------CAG-

+++ +++ +++++ + + + + ~ ~~~~ ~ ~~~ ~ ~~~~~ ~

HsKCNA615 ----AAAGTTAACCAGAAc

GgKCNA615

XtKCNA615 ----TGAGTTCAAGAGAAt

HsKCNA3210 C

TrKCNA3210b CcacTGAGTGTTCCAG

GaKCNA3210b ----TGAGTTTTGCAt

~ +++ + ++

No position present in all sequences.

21 of 79 positions (0.266) conserved in aligned fragments.

**Clique 680**:

| Homo sapiens | KCNA1-5 | 220046 | (56) |
| --- | --- | --- | --- |
| Homo sapiens | KCNA2-10 | 113525 | (59) |
| Gallus gallus | KCNA2-10 | 43142 | (41) |

HsKCNA615 a-------GCATCTTTCACGGATGCTCTCTTTTTCTGccctGCCCAGGCTCCTGGCCCCA

HsKCNA3210 tggaGCATGAATTATTGACAGCTTCACTTTCATCCTG----GCCCAGGCTGCTGGGCCGA

GgKCNA3210 tgagGCATGAATTATTCACGGCCTCGCTTTTATCTTG----GCCa

~ ~~~~* ** ** ** * * ** * * ** *** +++++ ++++ ++ +

HsKCNA615 GCa

HsKCNA3210 GCc

GgKCNA3210

++

18 of 34 positions (0.529) totally conserved.

32 of 63 positions (0.508) conserved in aligned fragments.

**Clique 682**:

| Homo sapiens | KCNA1-5 | 220972 | (39) |
| --- | --- | --- | --- |
| Xenopus tropicalis | KCNA1-5 | 117685 | (52) |
| Xenopus tropicalis | KCNA2-10 | 89864 | (68) |

HsKCNA615 GTATGTGATTGAATTGATGGGTTTCAGGTAT

XtKCNA615 TAGTACTAGTGATTGTATTTAtagTTTATGTATGTGAGTGTATAGATTGGTa

XtKCNA3210 TAGTATTGATGTTGGGATATAggtTTTATGTATGTGAGTGGATAGATAGGTGTCAGGCCT

+++++ + ++ + + ++ ++ +++++******** ** ** *** *** +++++ +

HsKCNA615 GGGCCCTa

XtKCNA615

XtKCNA3210 GGCCTCTg

++ + ++

18 of 23 positions (0.783) totally conserved.

48 of 68 positions (0.706) conserved in aligned fragments.

**Clique 683**:

| Homo sapiens | KCNA1-5 | 223818 | (62) |
| --- | --- | --- | --- |
| Xenopus tropicalis | KCNA2-10 | 96245 | (43) |
| Oryzias latipes | KCNA2a-10a | 42469 | (44) |

HsKCNA615 TGATGGCTtgagCCCAGTTAAGAGCAGACACCTCAGATAAAGAAATGGGATGCTGTCTTC

XtKCNA3210 TGATAGTT----CCCCTTTAAGAGCAGAAAACTCAAATATAAAAATt

OlKCNA3210a TAACGACTGGTACCTCAGTGAAAAAGATAGGATTGTATTTTT

++++ + + +++ +*** * * * **** * * * ** ++++ + + ++

HsKCNA615 Cc

XtKCNA3210

OlKCNA3210a Ct

+

15 of 29 positions (0.517) totally conserved.

34 of 62 positions (0.548) conserved in aligned fragments.

**Clique 684**:

| Homo sapiens | KCNA1-5 | 225394 | (21) |
| --- | --- | --- | --- |
| Gallus gallus | KCNA1-5 | 161945 | (33) |
| Homo sapiens | KCNA2-10 | 135905 | (23) |

HsKCNA615 ACTGCAGGGACAGCCAAGGAa

GgKCNA615 ccTCACCCAATCACTGCAGGGACAGACAAGGAc

HsKCNA3210 caTCAACCAACCACTGCAGGGAg

+ +++ ++++ +********** ++ ++++++

10 of 11 positions (0.909) totally conserved.

27 of 33 positions (0.818) conserved in aligned fragments.

**Clique 695**:

| Homo sapiens | KCNA1-5 | 241362 | (25) |
| --- | --- | --- | --- |
| Gallus gallus | KCNA1-5 | 151143 | (28) |
| Xenopus tropicalis | KCNA1-5 | 75672 | (55) |
| Takifugu rubripes | KCNA1a-5a | 6522 | (398) |
| Tetraodon nigroviridis | KCNA1a-5a | 17683 | (333) |
| Oryzias latipes | KCNA1a-5a | 8525 | (381) |
| Gasterosteus aculeatus | KCNA1a-5a | 7704 | (291) |
| Danio rerio | KCNA1a-5a | 10845 | (81) |
| Danio rerio | KCNA2b-10b | 85455 | (40) |

HsKCNA615

GgKCNA615 GAGCTGTCA-----TAC------------------

XtKCNA615

TrKCNA615a AGATAGAAACATAATTATCTTTACGGGACTGTCA-----TATATGGAGTAGACCATCA-A

TnKCNA615a GGGACCGTCA-----TACATGGAGTAGACCtgtcgA

OlKCNA615a ACATGAGGTGAACCATCA-G

GaKCNA615a AGTTAGGAACTTTGGGATCTCTGTGGAACTGTCAtactgTATGTGGAGTAGACCATCA-G

DrKCNA615a c-----------------------------------------------------------

DrKCNA3210b A-----TTC------------------

~ ~~~ ~~~ ~ ~~~~ ~ ~~ ~ ~~~~ ~ ~~ ~~ ~~~

HsKCNA615 CTGGTGGCGTCAGGCCT-------TGCT

GgKCNA615 ------TT----------------------------------------------------

XtKCNA615

TrKCNA615a GAAGGAACGCTTCATTTAGATCCCCAAAGTACTCAGTGGCGTTACGGCTACGACTCTGCT

TnKCNA615a GAAGGAACGCTTCATTTACCTCCCCAAAGTACTCTGTGGCAGTACGGCCACGGCTATGCT

OlKCNA615a TTAGTAATGCTTGATTTACTCCTCCAAAGCACTCTTAAGCACTAAGCCTCCAACCATGCT

GaKCNA615a TTAGGAATGCTTCATTTACCTCCCCAAAGCGCCCGATGGCATTAGGCCT-------TGCT

DrKCNA615a ------------------------------------------------------------

DrKCNA3210b ------ATACAGGATTAATTCCTTCAAAGTACTTATAAa---------------------

~~ ~ ~ ~~~ ~ ~ ~~~~~ ~ ~ ~ ~ ~ ~ ~ ~~~~

HsKCNA615 TCGt

GgKCNA615 ------------------------------------------------------------

XtKCNA615

TrKCNA615a CAGCTACGTA-GA--------------------TCAAAGCTCATAGAGTATATGACAGCT

TnKCNA615a CAGCTTGGTA-GG--------------------ACAAAGCTCATGTAGTACACGACAGCT

OlKCNA615a CAGCTAtacttGAAAGGAAGCTGAAAAAatt-------GCTTCTGAAGTACATGCCATGT

GaKCNA615a CCGCTACATG-GAAAGAAATCAGAAAAAtccaaACAAAGCTCTCAGAGTATGTGACAAGT

DrKCNA615a ------------------------------------------------------------

DrKCNA3210b ------------------------------------------------------------

~ ~ ~ ~~~ ~~ ~ ~~~~~~ ~~~~~~~ ~~~~ ~ ~~ ~

HsKCNA615

GgKCNA615 ------------------------------------------------------------

XtKCNA615

TrKCNA615a AGATAGAATAATAAATTCCTCCCATCCTAAAAACATATAAGAGC--ATCAACAAGA-AAA

TnKCNA615a AGATAGAATAACAAATTCTTCatcatCTTAAAACGTACAAGAttttATCAACAttagAAA

OlKCNA615a AGAAAGAACAACTTATTCTACCAATCCTAGAAATTTATAGTCAT--TTCTAAAAGA-AAA

GaKCNA615a AGATAGAATAATTAATTCTTCGGATCCTACAAACATATAGTTAC--ATCAAAAACT-Att

DrKCNA615a ------------------------------------------------------------

DrKCNA3210b ------------------------------------------------------------

~~~ ~~~~ ~~ ~~~~ ~ ~~ ~~~ ~~ ~ ~~ ~ ~ ~

HsKCNA615

GgKCNA615 ------------------------------------------------------------

XtKCNA615

TrKCNA615a CACCAATTATTCCACACGT--AAGGACCCCTGGAGGACATGGGACGCATACTGTCTTATC

TnKCNA615a CATCAATTATTCCACATGT--AAGGATCCCTGGAGGACACGGAACGCATCCTGTCCTATC

OlKCNA615a CATTGATCATAACAGATATACACGGCTCCCTAGAGGACACTGAGTGCATCTAGTCTTATC

GaKCNA615a ccgcg-----------TACACACGGCTCCCCGGAAGACGCAGAGCAAACACTGTCTCATC

DrKCNA615a ------------------------------------------------------------

DrKCNA3210b ------------------------------------------------------------

~ ~~ ~~ ~~ ~ ~~~ ~~ ~~~ ~~ ~~~ ~ ~ ~~~ ~~~

HsKCNA615

GgKCNA615 ----------------------------TGCATGGAG-----------------------

XtKCNA615 TGCATGGAGTt-CTATTAGATGTCAGCCAACC

TrKCNA615a ATTTGACAGTGTGATAATTAAAATCATATGCATGGAGTACCAATTACATTTCAGCAAGCT

TnKCNA615a GTTTGACAGCGTGATAATTAAAATCATATGCATGGAGTACCAATAACATTTCAGCAAGCT

OlKCNA615a AGAgacaGGCGTAATAATTAAAATCTTATGCATGGAGTACAGATAACATTTCAGCAAGTT

GaKCNA615a GGAcg--------------------------ATGGAGTga

DrKCNA615a ----GACGGCGGGATTACTAAAACATCACGCATGGACTACAGAcTACATTTCAGCAAGCT

DrKCNA3210b --------------------------------------------TAG

~ ~ ~~ ~ ~~~~~ ~ ~~~~~~~ ~ ~ ~ ~~ ~~~~~ ~

HsKCNA615

GgKCNA615 -----CTGag

XtKCNA615 ACGCACTGCATTCAAG-------------------------------------------G

TrKCNA615a GCACAACGCATCCAAACGGTCTGCAgagacatgc--CAACATCCGCAGTCGAGTGTTAAA

TnKCNA615a GCACAATGCATCCAAACGGTCTGCA

OlKCNA615a GCACAATGCAccagaaacgactacacacgcaatcctCAGCAACCACGGTTAACCGCCAAG

GaKCNA615a

DrKCNA615a GCACACTGCATCTGGG-------------------------------------------A

DrKCNA3210b

~ ~~ + ~ ~~ ~~ ~ ~~ ~ ~~ ~~ ~~ ~ ~~ ~ ~ ~~

HsKCNA615

GgKCNA615

XtKCNA615 AAGTact

TrKCNA615a AACTTCGATGAC

TnKCNA615a

OlKCNA615a AAGTCCGATGAC

GaKCNA615a

DrKCNA615a AAGTgca

DrKCNA3210b

++ + + +++++

No position present in all sequences.

10 of 432 positions (0.023) conserved in aligned fragments.

**Clique 702**:

| Homo sapiens | KCNA1-5 | 255046 | (30) |
| --- | --- | --- | --- |
| Gasterosteus aculeatus | KCNA2b-10b | 27670 | (39) |
| Danio rerio | KCNA2b-10b | 79669 | (47) |

HsKCNA615 CAACAGGTCGTTGATGTTACCGTTGAgcat

GaKCNA3210b ctc--------TGCTGTTGCTGTTGAATAAAATTAGAAACAGATGAt

DrKCNA3210b CAAAAGGTCGTTGCTGTTACAGTTGAATAATGTAGGAAATAAATAAa

* ~~~~~~~** **** * ***** * + ++++ + ++ +

14 of 22 positions (0.636) totally conserved.

23 of 47 positions (0.489) conserved in aligned fragments.

**Clique 705**:

| Homo sapiens | KCNA1-5 | 260764 | (53) |
| --- | --- | --- | --- |
| Gasterosteus aculeatus | KCNA2a-10a | 24408 | (78) |
| Oryzias latipes | KCNA2a-10a | 43226 | (85) |
| Takifugu rubripes | KCNA2a-10a | 21904 | (33) |
| Tetraodon nigroviridis | KCNA2a-10a | 23595 | (45) |

HsKCNA615 t------------------------------------------ATATTCAGTtga---AT

GaKCNA3210a ttacgaa---TGCACGTAGATCACAAATATGCCCCAGACAGACATACTgg----------

OlKCNA3210a ACAa-----ACCATTCTGCTCCAGCAAGACATAGTAAGTcagaagAC

TrKCNA3210a CGGCTGCACAGAGACCCCAAATCTGCCCCAACc

TnKCNA3210a tcagagCGGCTGCACAGAGACCCCAAATCTGCCTCCAACAAACAC

+ ~ ~~~~~~~~ ~~~ ~ ~ ~ ~ ~~~ ~ ~ ~~+ + + ~~ ~

HsKCNA615 ATACttTATATTCAAAAGTAGTTAATGAATGCCTTCCt

GaKCNA3210a ------------CAAAAATATTTTA--AATGTTTTCTAGCATTTt

OlKCNA3210a ATAC--TATTTACAAAAATAGTTAGTTGATGTCTTCCAGCAGTTc

TrKCNA3210a

TnKCNA3210a

~~~~ ~~~ ~ +++++ ++ ++ ~ +++ +++ +++ ++

No position present in all sequences.

24 of 105 positions (0.229) conserved in aligned fragments.

**Clique 708**:

| Homo sapiens | KCNA1-5 | 261973 | (29) |
| --- | --- | --- | --- |
| Gasterosteus aculeatus | KCNA2a-10a | 27290 | (113) |
| Oryzias latipes | KCNA2a-10a | 49049 | (114) |

HsKCNA615 tg----------------------------------------------------------

GaKCNA3210a ATTTAT--TTATAAGTCAGATATGGATAAAGCCTTtttGTGTGTAGGGTGACTAAGCCAA

OlKCNA3210a ATCTATtgTAATACCTTAAATAAGGATAAAACATTcccGGGTGTAGGGTAAGTGAGTCAA

~~~ ~ ~~~ ~ ~ ~~~ ~~~~~~~ ~ ~~ ~ ~~~~~~~~~ ~ ~ ~~ ~~~

HsKCNA615 ---------------------------CTG--GTCCAGCCTAAGAAGTCCTGAGCC

GaKCNA3210a GCAT-GAGAGATGTGGAGATCTAATCGCTccaTTGCACCCTAGAAAGCCTTGAACA

OlKCNA3210a GCATgGAGAAATCTGAGGATCTAATCACTG--TTCCAGTCTACAAAGCCTTGAGCA

~~~~ ~~~~ ~~ ~~ ~~~~~~~~~ ** * ** *** *** * *** *

16 of 29 positions (0.552) totally conserved.

16 of 116 positions (0.138) conserved in aligned fragments.

**Clique 711**:

| Homo sapiens | KCNA1-5 | 264391 | (31) |
| --- | --- | --- | --- |
| Gallus gallus | KCNA1-5 | 169230 | (25) |
| Homo sapiens | KCNA2-10 | 144915 | (31) |

HsKCNA615 TGTCTGCAAGCTCAGTAAGTCCCAGGCTCCc

GgKCNA615 GTGAGTCAGCTCAGACAAGCCAAGc

HsKCNA3210 TGTATaTCAGCTCAGTCAGGCCAAGGTTCCa

+** ******* * ** ** +++

14 of 25 positions (0.560) totally conserved.

18 of 31 positions (0.581) conserved in aligned fragments.

**Clique 713**:

| Takifugu rubripes | KCNA1a-5a | 9576 | (56) |
| --- | --- | --- | --- |
| Takifugu rubripes | KCNA2a-10a | 22431 | (54) |
| Danio rerio | KCNA2b-10b | 64269 | (57) |

TrKCNA615a AACCACTGTTTAACGATATATATACA-----TATATATACAACACGC--CCTGTCACGGT

TrKCNA3210a AAATAATAGTTATTAATATGTTCACAcatgtTATACATTCAACACGC--TCTGTCT

DrKCNA3210b AACCGATATTTg-CTATATATACACA-----CATTTATATAAAACagtaTCTATCTCTAT

** * ** **** * *** ** ** ** ** ** ** + +

TrKCNA615a TTt

TrKCNA3210a

DrKCNA3210b TTa

++

25 of 48 positions (0.521) totally conserved.

29 of 63 positions (0.460) conserved in aligned fragments.

**Clique 718**:

| Gallus gallus | KCNA1-5 | 198924 | (54) |
| --- | --- | --- | --- |
| Homo sapiens | KCNA2-10 | 132315 | (74) |
| Danio rerio | KCNA2b-10b | 81745 | (71) |

GgKCNA615 TCTCAGAggCAAA---TTACAGATCATAGAc----------AAATCATCATTTATAAT

HsKCNA3210 ATTTTTGGAC-CTAAgtcTTACAAGTTGTCCAAAAATTCAATGGCATCccaTTCTGTGAC

DrKCNA3210b ACTCTTAGAC-CGAA---TTACATGTTATTGAATGTCTCAAGTAAATCATCTTTTGTAAC

+ * * ** * ** ***** * * * ~~~~ *** * * * *

GgKCNA615 AATCATTCa

HsKCNA3210 ACTGATTCTTCTTTC

DrKCNA3210b AGTCATTCTTCTTTG

* * **** +++++

**28 of 53 positions (0.528) totally conserved.**

**34 of 75 positions (0.453) conserved in aligned fragments.**

**Clique 719**:

| Tetraodon nigroviridis | KCNA1a-5a | 19165 | (51) |
| --- | --- | --- | --- |
| Homo sapiens | KCNA2-10 | 81532 | (97) |
| Gallus gallus | KCNA2-10 | 34118 | (122) |

TnKCNA615a GGAACATCCATAGGTAGGAAAC--------------------------------------

HsKCNA3210 GTCAGTAGGGtctGATATTTGTACATAGGTtATA

GgKCNA3210 GGCACATCCATGGGTTGGAAACccacGTCAATAGGGcc-GATCATTGTATATAGGT-ATA

++ ++++++++ +++ ++++++ ~~~~ ~~~~~ ~ ~~~ ~~~~~ ~~~~~~ ~~~

TnKCNA615a ---------GTACAgTATGTCATTC--Agg-------------CTGGTTTttt

HsKCNA3210 CATATATGTGTACA-TATGTGCCTC--AATGAACATTGCCTGTCCACCTGATCATGGGTG

GgKCNA3210 CAGATCTGTGTACA-TAcat-ATTCcaAATGAGCATTTGCTGTCTGGTTTATCATGAGTG

~~ ~~ ~~~***** ** * ** * ~~ ~~~~ ~~~~* * * +++ +++

TnKCNA615a

HsKCNA3210 TACATA

GgKCNA3210 TATATA

++ +++

14 of 27 positions (0.519) totally conserved.

44 of 126 positions (0.349) conserved in aligned fragments.

**Clique 724**:

| Xenopus tropicalis | KCNA2-10 | 153564 | (31) |
| --- | --- | --- | --- |
| Oryzias latipes | KCNA2a-10a | 44176 | (58) |
| Danio rerio | KCNA2b-10b | 72186 | (35) |

XtKCNA3210 ca-----------------------GACAATgt----AATTGCTGTGAAATTCAGCAt

OlKCNA3210a ggatCTCTGTGTGTATGCATAGTGTGCCAATcaacacAATTGCTGTCAAATGTAGCAc

DrKCNA3210b gcgcCTTTGTGTTTATGCATTGTGTTTCAGT----------------AAAT

~~ ~~~~~ ~~~~~~~ ~~~~ ** * ~~~~~~~~~ **** ++++

7 of 12 positions (0.583) totally conserved.

11 of 58 positions (0.190) conserved in aligned fragments.
